# Neuronal cell fate specification by the molecular convergence of different spatio-temporal cues on a common initiator terminal selector gene

**DOI:** 10.1371/journal.pgen.1006729

**Published:** 2017-04-17

**Authors:** Johannes Stratmann, Stefan Thor

**Affiliations:** Department of Clinical and Experimental Medicine, Linköping University, Linköping, Sweden; New York University, UNITED STATES

## Abstract

The extensive genetic regulatory flows underlying specification of different neuronal subtypes are not well understood at the molecular level. The Nplp1 neuropeptide neurons in the developing *Drosophila* nerve cord belong to two sub-classes; Tv1 and dAp neurons, generated by two distinct progenitors. Nplp1 neurons are specified by spatial cues; the Hox homeotic network and GATA factor *grn*, and temporal cues; the *hb* -> *Kr* -> *Pdm* -> *cas* -> *grh* temporal cascade. These spatio-temporal cues combine into two distinct codes; one for Tv1 and one for dAp neurons that activate a common terminal selector feedforward cascade of *col* -> *ap*/*eya* -> *dimm* -> *Nplp1*. Here, we molecularly decode the specification of Nplp1 neurons, and find that the cis-regulatory organization of *col* functions as an integratory node for the different spatio-temporal combinatorial codes. These findings may provide a logical framework for addressing spatio-temporal control of neuronal sub-type specification in other systems.

## Author summary

The nervous system contains a myriad of different cell types. These are specified by elaborate transcription factor cascades, starting with early factors that provide spatial and temporal information, to late factors that dictate final cell identity. The molecular nature of such cascades is poorly understood in any system. We focus on two related neuropeptide neurons in the *Drosophila* central nervous system, for which an extensive genetic pathway has been identified. We identify the enhancers for the different genes in the cascade, and conduct an extensive molecular analysis of these. Our findings reveal that different spatial and temporal cues converge on different enhancers of a key initiator terminal selector gene, which then triggers a feedforward cascade of sequential enhancer activation, ultimately landing on the enhancer of the neuropeptide gene. These findings may point to general mechanisms underlying specification of unique neuronal cell fate in many systems.

## Introduction

The nervous system contains a myriad of different neuronal sub-types, and understanding cell fate specification remains a major challenge. Studies in a number of systems have revealed that neuronal subtype specification relies upon complex cascades of regulatory information, involving spatial and temporal selector genes [1], onwards to terminal selector genes [2, 3], often acting in combinatorial codes [4–6]. With respect to spatial information, the Hox homeotic selector genes, expressed in distinct but partly overlapping domains along the antero-posterior axis of the central nervous system, have been extensively studied for their role in cell fate specification [reviewed in [7, 8]]. With regard to temporal information, seminal studies in the *Drosophila* embryonic central nervous system (CNS) has identified a temporal cascade, where the sequential expression of the transcription factors Hunchback (Hb), Kruppel (Kr), Pdm2 and Nubbin (collectively referred to as Pdm), Castor (Cas) and Grainy head (Grh) play out in most, if not all neuroblasts (NBs) [reviewed in [9]]. The temporal factors dictate the identity of neurons and glia being specified at different stages of NB lineage progression. Although not conserved in its entirety, research in mammals has pointed to similar temporal progressions, and begun identifying some of the factors involved [reviewed in [10]]. In addition, studies have revealed that the Hox spatial information can converge with temporal cues to thereby specify neuronal subtypes [11]. While these functional genetic studies have provided insight into the genetic mechanisms underlying neuronal subtype specification, it is largely unclear how the broader spatio-temporal cues are molecularly integrated to cause discrete terminal selector gene expression, and how terminal selectors feed forward to final cell identity.

**Fig 1:**
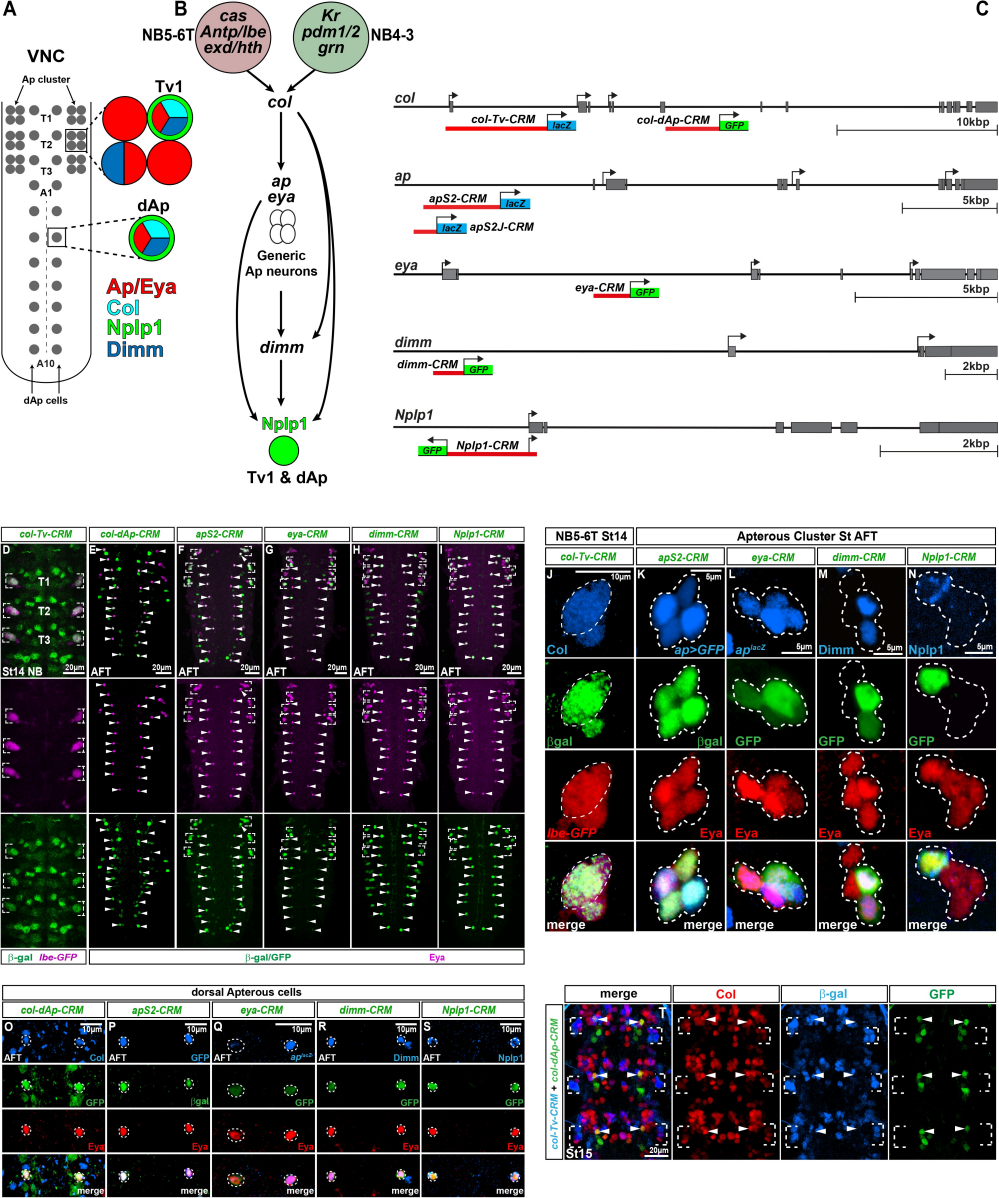
Identification of enhancers for the Nplp1 specification cascade. (A) Model of the *Drosophila* VNC at stage AFT, with focus on the thoracic Ap clusters and the dorsal Ap cells (dAp), together with corresponding markers expressed at stage AFT i.e. Col, Ap/Eya, Dimm and Nplp1. (B) Showing the feedforward regulatory cascade critical for terminal specification of both the Tv1/Nplp1 and dAp/Nplp1. In Tv1 specification *col* is activated by spatial input via *Antp*, *lbe*, *hth*/*exd* together with temporal input from *cas*. In dAp specification *col* is activated via the temporal factors *Kr*, *pdm1/2* together with spatial input from *grn*. Once *col* is activated in both cell subtypes, the same Nplp1 terminal selector cascade is triggered in both dAp and Tv1 to specify the Nplp1 cell fate. (C) Enhancer-reporter constructs used to study cell type specific expression of the factors critical during Nplp1 cell fate specification in both dAp and Tv1 cells. *col* expression is under the control of two different enhancers. Expression of *col* in the NB5-6T is controlled by the *col-Tv-CRM* while *col* expression in the dAp cells is controlled by the *col-dAp-CRM*. For *ap* enhancer studies we used two different enhancer fragments. To test the enhancer in mutant background we used the *apS2-lacZ* and for mutation of transcription factor binding sites the shorter *apSJ2-lacZ* was used. The *Nplp1-CRM* contains the promoter for *Nplp1*, and in order to avoid ectopic expression of GFP from the Nplp1 promoter, the DNA sequence of the *Nplp1-CRM* was placed in reverse orientation in front of the GFP reporter. (D) Staining of the *col-Tv-CRM* reporter construct in T1-T3 for β-gal and *lbe(K)-GFP* (NB5-6T) (false colored) shows that the *col-Tv-CRM* drives reporter expression broadly in the thoracic region but also specifically in the NB5-6T. (E-I) Staining for β-gal or GFP as a readout of enhancer activity and Eya as a location marker on whole VNCs shows overlap between the reporter expression and the Ap (both Ap cluster and dAp) cells. (J) Zoom in on the NB5-6T shows a robust overlap between the *col-Tv-CRM* and endogenous Col expression at stage14. (K-N) Detailed analysis of the enhancer expression in the Ap cluster at stage AFT shows a precise overlap between the *apS*, *eya*, *dimm* and *Nplp1* enhancer-reporter constructs and endogenous gene expression (compare to A). (O-S) Detailed analysis of the enhancer expression in the dAp cells at stage AFT shows a precise overlap between the *col-dAp*, *apS*, *eya*, *dimm* and *Nplp1* enhancer-reporter constructs and endogenous gene expression. (T) Triple stain at St15 for β-gal, GFP and Col, for the *col-Tv-CRM* and *col-dAp-CRM*, reveals that the separate *col* enhancer fragments do not overlap and show cell type specific reporter expression. Both fragments show overlapping activity with endogenous *col* expression. Genotypes: (D, J) *col-CRM-lacZ/lbe(K)-GFP*. (E) *col-dAp-CRM-GFP*. (F) *apS-CRM-lacZ*. (G) *eya-CRM-GFP*. (H, M, R) *dimm-CRM-GFP*. (I, N, S) *Nplp1-CRM-GFP*. (K, P) *ap-Gal4>UAS-eGFP; apS2-CRM/+*. (L, Q) *ap*^*rK568*^*/+; eya-CRM/+*. (T) *col-CRM-lacZ/+; col-dAp-CRM-GFP/+*.

The *Drosophila* ventral nerve cord (VNC; defined here as thoracic segments T1-T3 and abdominal A1-A10) contains ~10,000 cells at the end of embryogenesis, which are generated by a defined set of ~800 neuroblasts (NBs) [12–16]. The Apterous neurons constitute a small sub-group of interneurons, identifiable by the selective expression of the Apterous (Ap) LIM-homeodomain factor, as well as the Eyes absent (Eya) transcriptional co-factor and nuclear phosphatase (Fig 1A) [17, 18]. A subset of Ap neurons express the Nplp1 neuropeptide, but can be sub-divided into the lateral thoracic Tv1 neurons, part of the thoracic Ap cluster of four cells, and the dorsal medial row of dAp neurons (Fig 1A) [6, 19]. In line with the distinct location of the Tv1 and dAp neurons, studies have revealed that they are generated by distinct NBs; NB5-6T and NB4-3, respectively [20, 21]. A number of studies have addressed the genetic mechanisms underlying the specification of the Tv1 and dAp neurons, and the regulation of the Nplp1 neuropeptide. These have revealed that two distinct spatio-temporal combinatorial transcription factor codes, one acting in NB5-6T and the other in NB4-3, converge on a common initiator terminal selector gene; *collier* (*col*; Flybase *knot*), encoding a COE/EBF transcription factor (Fig 1B) [20–22]. Col in turn is necessary and sufficient to trigger a feed forward loop (FFL) consisting of Ap, Eya and the Dimmed (Dimm) bHLH transcription factor, which ultimately activates the Nplp1 gene [6]. Strikingly, the combinatorial coding selectivity of the spatio-temporal cues combined with the information-coding capacity of the FFL results in the selective activation of Nplp1 in only 28 out of the ~10,000 cells within the VNC. While these genetic studies have helped resolve the regulatory logic of this cell specification event, they have not addressed the molecular mechanisms by which the two different spatio-temporal combinatorial codes intersect upon the *col* initiator terminal selector, to trigger a common terminal FFL, or the molecular nature of the FFL.

To address this issue, we have identified enhancers for Tv and dAp neuron expression for the genes in the common Tv1/dAp FFL: *col*, *ap*, *eya*, *dimm* and *Nplp1*. We generated transgenic reporters for these enhancers, both wildtype and mutant for specific transcription factor binding sites, to test their regulation in mutant and misexpression backgrounds. We also used CRISPR/Cas9 technology to delete these enhancers in their normal genomic location to test their necessity for gene regulation. Strikingly, we find that the distinct upstream spatio-temporal combinatorial codes, which trigger *col* expression in Tv1 versus dAp neurons, converge onto different enhancer elements in the *col* gene. Hence, the *col* Tv1 neuron enhancer is triggered by *Antp*, *hth*, *exd*, *lbe* and *cas*, while the dAp enhancer is triggered by *Kr*, *pdm* and *grn*. In contrast to this subset-specific enhancer set-up for *col* activation, the subsequent, *col*-driven Nplp1 FFL feeds onto common enhancers in each downstream gene. These findings reveal that distinct spatio-temporal cues, acting in different neural progenitors, can trigger the same FFL by converging on discrete enhancer elements in an initiator terminal selector, to thereby dictate the same ultimate neuronal subtype cell fate.

## Results

### Identification of enhancers for the terminal selectors

The Ap neurons constitute a set of interneurons in the *Drosophila* VNC, out of which the thoracic lateral Tv1 neurons and the dorso-medial dAp neurons express the Nplp1 neuropeptide (Fig 1A) [6, 17, 19]. Tv1 neurons are generated by NB5-6T, while dAp neurons arise from NB4-3 [6, 21]. Activation of Nplp1 in Tv1 and dAp neurons is controlled by a shared coherent FFL, consisting of *col*, *ap*, *eya* and *dimm*, where *col* is both necessary and sufficient to trigger the FFL [6, 21]. In contrast, this common FFL is triggered by two different upstream spatio-temporal combinatorial codes, acting in the two different NBs. In NB5-6T this includes the temporal gene *castor* (*cas*), the Hox homeotic gene *Antennapedia* (*Antp*), the two Hox co-factor genes *homothorax* (*hth*) and *extradenticle* (*exd*), as well as the homeobox gene *ladybird early* (*lbe*). In NB4-3, this includes the temporal genes *Kruppel* (*Kr*) and *pdm2/nub* (*pdm*), as well as the GATA gene *grain* (*grn*), (Fig 1B) [20–22].

To identify the cell-specific *cis*-regulatory modules (CRMs) that act as enhancers for the five genes in the dAp/Tv1 FFL, we analyzed expression of a number of transgenic lines generated in previous studies [17, 23, 24], as well as an *eya-CRM-Gal4* transgene (provided by T. Lian and D.W. Allan; S1 Fig). This resulted in identification of fragments capable of driving reporter gene expression in the Tv1 and dAp neurons. To facilitate mutagenesis of CRMs, we attempted to identify smaller genomic fragments that retained appropriate activity. This resulted in the identification of smaller (1–2 kilobases) CRMs for all genes with the exception of *col*, where larger fragments were required for proper expression (Fig 1C–1S, S1 Fig, S1 Data). Strikingly, we found that while one enhancer region was sufficient to recapitulate Tv1 and dAp expression of *ap*, *eya*, *dimm* and *eya*, for *col* we identified two distinct enhancers, one each for expression in dAp or Tv1 neurons (Fig 1C–1E and 1T).

### CRISPR/Cas9 deletion of enhancers affects cell type specific expression of terminal selectors

**Fig 2:**
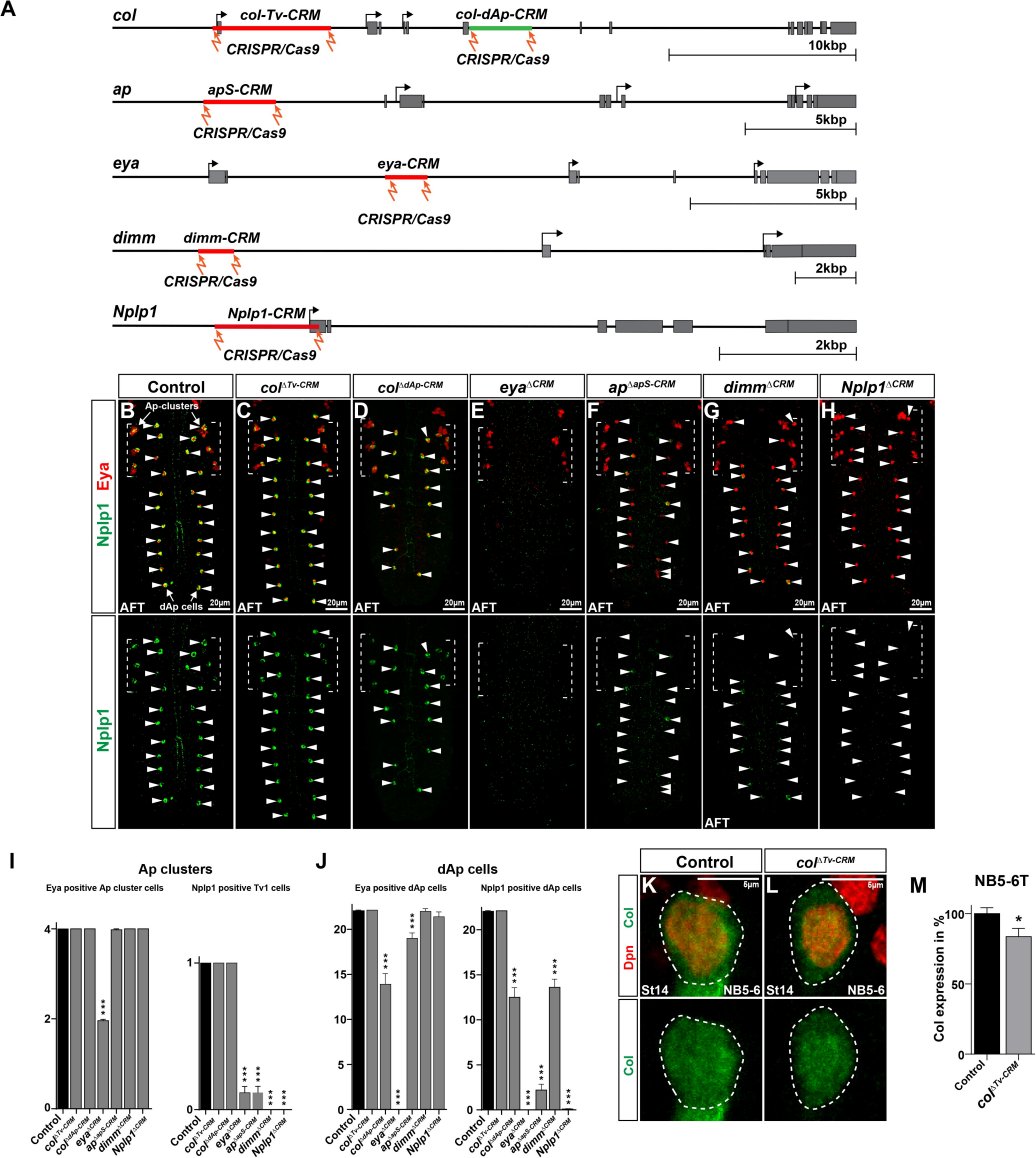
CRISPR/Cas9 deletion of enhancers affects Tv1 and dAp specification. (A) Schematic representation of the CRISPR/Cas9 mutant experiments, showing the gene loci of the genes which are part of the terminal selector cascade of Nplp1 cell fate specification in Tv1 and dAp cells. Red bars represent the location of the identified enhancers. In the *col* gene, two independent enhancers were identified, in red the *col-Tv-CRM*, active in the NB5-6T and the Ap cluster (compare to Fig 1T) and in green the *col-dAp-CRM* specifically active in NB4-3 and the dAp cells. “Lightning bolts” represent the location to which the Cas9 protein is guided by gRNAs in order to induce enhancer deletions. (B-H) Whole VNC scans showing Eya and Nplp1 expression in control and CRM mutants for the identified enhancers. (I) Quantification of Eya and Nplp1 expressing cells in the Ap cluster, in control and CRM mutants. *eya*^*ΔCRM*^ mutants show a significant decrease in the number of Eya positive Ap cluster cells, while the other mutants have no impact on Eya cell numbers in the Ap clusters. *eya*^*ΔCRM*^, *ap*^*ΔapS-CRM*^, *dimm*^*ΔCRM*^ and *Nplp1*^*ΔCRM*^ mutants all affect Nplp1 expression in Tv1 (_***_ p≤0.0001, n≥44 clusters, Students t-test, +/- SEM). (J) Quantification of Eya and Nplp1 positive dAp cells in control and CRM mutants. *col*^*ΔdAp-CRM*^, *eya*^*ΔCRM*^, and *ap*^*ΔapS-CRM*^ show significant decrease in Eya positive dAp cells, while *col*^*ΔTv-CRM*^, *dimm*^*ΔCRM*^ and *Nplp1*^*ΔCRM*^ mutants show no effect. In contrast, all CRM mutants shows significantly reduced numbers of Nplp1 positive dAp cells (_***_ p≤0.0001, n = 10 embryos, Students t-test, +/- SEM). (K-L) Staining for Col and Dpn in the NB5-6 at St14, in control and *col*^*ΔTv-CRM*^ mutants. (M) Quantification of Col expression levels in the NB shows that Col levels are significantly reduced by 17% in *col*^*ΔTv-CRM*^ mutants (_*_ p = 0.013, n = 36 NBs, Students t-test, +/- SEM). Genotypes: (B, K) *OregonR*. (C, L) *col*^*ΔTv-CRM*^. (D) *col*^*ΔdAp-CRM*^. (E) *eya*^*ΔCRM*^. (F) *ap*^*ΔapS-CRM*^. (G) *dimm*^*ΔCRM*^. (H) *Nplp1*^*ΔCRM*^.

Enhancer studies have revealed that some genes may be controlled by several enhancers with partially redundant function, such as ‘shadow enhancers’, which act to ensure high-fidelity in gene expression [25]. These shadow enhancers have been identified in a growing number of genes, in particular early developmental regulators [26, 27]. We wanted to address the importance of the identified enhancers within the context of their normal genomic location. To this end, we used CRISPR/Cas9 technology, with two spaced gRNAs, to delete each of the identified enhancers in the FFL (Fig 2A; Materials and Methods; S2 Data) [28].

Focusing on *col* first, we analyzed the *col-dAp-CRM* (generating the *col*^*ΔdAp-CRM*^ deletion mutant) and observed that deletion of this enhancer resulted in significant loss of Col, Eya and Nplp1 expression, but as anticipated only in dAp and not in Tv1 neurons (Figs 2D, 2I, 2J, S2C and S2F–S2H). In contrast, we found that deletion of the *col-Tv-CRM* (*col*^*ΔTv-CRM*^) did not result in any effect upon Eya or Nplp1, in either dAp or Tv1 neurons (Fig 2B, 2C, 2I and 2J). We furthermore did not observe any effects on Col expression itself, either within the Ap cluster at AFT or globally (S2A, S2B, S2D, S2E and S2G Fig). As anticipated, we also did not observe any effects on Eya, Nplp1 or Col expression in dAp neurons (Figs 2B, 2C, 2I, 2J, S2E and S2H). Given the specificity of this element when placed in a promoter-*lacZ* transgenic construct (Fig 1D and 1J), we found this lack of effect surprising. This prompted us to analyze the NB5-6T neuroblast at St14, right after the onset of endogenous Col expression in this lineage. Measuring Col expression levels in the *col*^*ΔTv-CRM*^ mutants we did indeed observe a minor but significant reduction in expression (Fig 2K–2M).

Next, we analyzed *eya*, *ap*, *dimm* and *Nplp1* enhancer deletions (*eya*^*ΔCRM*^, *ap*^*ΔapS-CRM*^, *dimm*^*ΔCRM*^, *Nplp1*^*ΔCRM*^), and observed that all exhibited strong effects. Specifically, as anticipated, all enhancer deletions resulted in significant reduction or loss of expression of the targeted gene, in both Tv1 and dAp neurons (Figs 2E, 2H and S2I–S2X). Moreover, in line with the previous genetic analysis that identified an *eya*/*ap* ->*dimm* ->*Nplp1* FFL, deletion of the *eya*, *ap* or *dimm* enhancers all significantly reduced Nplp1 expression (Figs 2E–2J, S2I–S2N and S2Q–S2U). Also in line with this FFL, deletion of *ap* or *eya* enhancers did not affect one another’s expression (Figs 2F and S2J–S2P). Within the Ap cluster, while deletion of the *eya* enhancer affected Nplp1 expression in Tv1, we did observe Eya expression in two cells in the cluster (S2J Fig). However, analysis at AFT, using Col as a specific Tv1 marker at this stage, revealed that Eya expression was lost from the Tv1 neuron, hence explaining the strong effect on Nplp1 in *eya*^*ΔCRM*^ mutants (S3A and S3B Fig). In line with the previous genetic analysis, *dimm* enhancer deletion did not affect either Eya or Ap expression (Figs 2H–2J, S2R and S2U). Finally, deletion of the *Nplp1-CRM* did not affect expression of Eya, Ap or Dimm (Figs 2H–2J, S2S and S2V–S2X).

We conclude that activation of *col* in dAp neurons strongly depends upon the *col-dAp-CRM* element, while in contrast, expression of *col* in Tv1 neurons may operate via several enhancers, some of which presumably must reside outside of the *col-Tv-CRM*. In contrast, for the postmitotically expressed terminal selectors *ap*, *eya* and *dimm*, as well as the *Nplp1* neuropeptide gene, their expression in Ap neurons appears to be critically dependent upon one discrete enhancer element.

### Molecular analysis of the FFL enhancers

Having identified necessary and sufficient enhancers for the genes in the FFL, we proceeded to address the putative molecular connections between the upstream spatio-temporal cues and *col*, as well as between the FFL genes. This was approached by testing the enhancer transgenes in the pertinent mutant backgrounds, as well as mutating relevant candidate binding sites within each enhancer.

**Fig 3:**
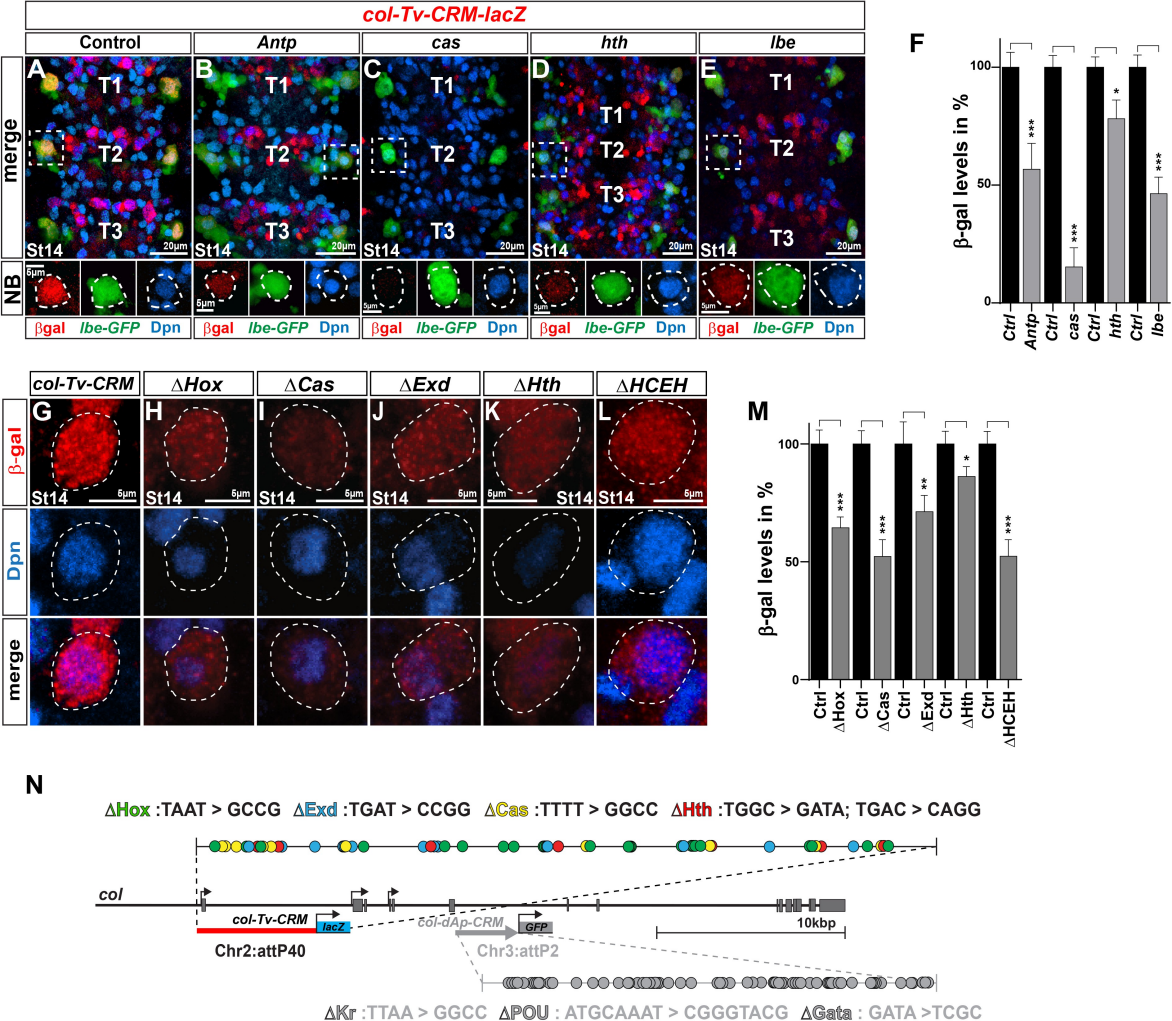
*col-Tv* enhancer analysis. (A-E) T1-T3 region of the *Drosophila* VNC and a zoom-in on the NB5-6 at stage 14, stained for Dpn, *lbe(K)-GFP* and β-gal to show the activity of the *col-Tv-CRM-lacZ* fragment under (A) control conditions and (B-E) in different mutant backgrounds for *col* upstream activators in NB5-6 i.e., *Antp*, *hth*, *cas* and *lbe*. Reporter expression under control of the *col-Tv-CRM* shows reduced expression in all mutant backgrounds. (F) Quantification of β-gal levels from the *col-Tv-CRM* in NB5-6 at stage 14 in different mutant backgrounds, shows that all mutant backgrounds affect the *col-Tv-CRM* activity and β-gal levels are reduced significantly compared to each control (_***_ p≤0.0001, _*_ p≤0.01, n≥30 NBs, Students t-test, +/- SEM). (G-L) Staining for β-gal and Dpn of the NB5-6 at stage14, to assess the *col-Tv-CRM* activity of (G) the *col-Tv-CRM* wild type fragment and (H-L) the *col-Tv-CRM* with mutated potential binding sites for the *col* upstream activators (indicated by Δ). (L) ΔHCEH denotes a *col-Tv-CRM* fragment with all potential binding sites mutated (Δ Hox, Cas, Exd, Hth). (M) Quantification of the β-gal levels in the NB5-6 shows that all *col-Tv-CRM* fragments with mutated binding sites for *col* upstream activators, show a significant decrease in activity compared to control levels. (_***_ p≤0.0001, _**_ p≤0.001, _*_ p≤0.05, n≥30 NBs, Students t-test, +/- SEM). (N) Schematic representation of the *col* genetic locus together with the position of the *col-Tv-CRM* fragment. Color coded dots represent potential transcription factor binding sites (TFBS) found in the *col-Tv-CRM* fragment, together with the conversion pattern, which was used to mutated the potential binding sites. Chr2:attP40 denotes the landing sites at which all, *col-CRM* as well as *col-Tv-CRM* ΔTFBS constructs are landed in the fly genome. Genotypes: (A, G) *col-Tv-CRM/lbe(K)-GFP*. (B) *col-Tv-CRM/lbe(K)-GFP; Antp*^*12*^*/Antp*^*25*^. (C) *col-Tv-CRM/lbe(K)-GFP; cas*^*Δ1*^*/cas*^*Δ3*^. (D) *col-Tv-CRM/lbe(K)-GFP; hth*^*Df7637*^*/hth*^*5E04*^. (E) *col-Tv-CRM/lbe(K)-GFP; lbe*^*12C5*^*/lbe*^*Df*^. (H) *col-Tv-CRM-*Δ*Hox*/*lbe(K)-GFP*. (I) *col-Tv-CRM-*Δ*cas*/*lbe(K)-GFP*. (J) *col-Tv-CRM-*Δ*exd*/*lbe(K)-GFP*. (K) *col-Tv-CRM-*Δ*hth*/*lbe(K)-GFP*. (L) *col-Tv-CRM-ΔHCEH*/*lbe(K)-GFP*.

Focusing on the *col* Tv enhancer first, we introduced the *col-Tv-CRM-lacZ* transgene into the *Antp*, *cas*, *hth* and *lbe* mutant backgrounds. This resulted in significant reduction of expression in all four cases, when compared to the enhancer transgene in a wild type background (control) stained on the same slide, with the strongest effect in *cas* mutants, which displayed a near-complete loss of expression (Fig 3A–3F). Next, we mutated conserved DNA-binding sequences for Antp, Cas, Hth and Exd within the *col-Tv-CRM-lacZ*, and integrated these into the same genomic location as the wild type transgenic construct (Fig 3M; Materials and Methods; S3 Data and S4 Data). We assayed β-gal expression in NB5-6T at St14, and found that all of the four mutated enhancer transgenes displayed reduced expression, when compared to the enhancer transgene in a wild type background (control) stained on the same slide (Fig 3G–3M).

**Fig 4:**
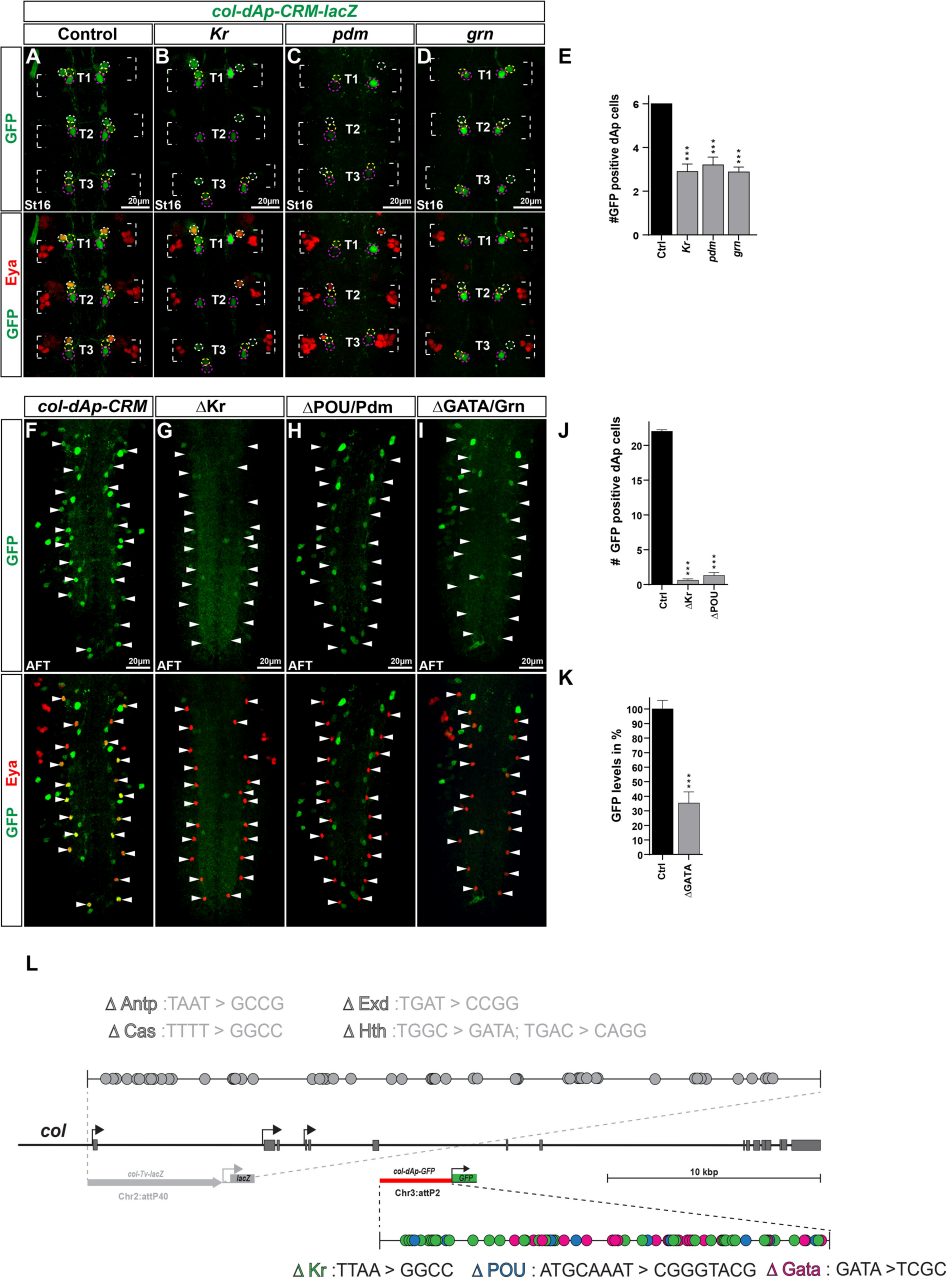
*col-dAp* enhancer analysis. (A-D) T1-T3 region of the *Drosophila* VNC at St16, stained for Eya and GFP to show the activity of the *col-dAp-CRM-GFP* fragment under (A) control conditions and (B-D) in different mutant backgrounds for *col* upstream activators in NB4-3 i.e., *Kr*, *pdm* and *grn*. GFP reporter expression under control of the *col-dAp-CRM* shows reduced expression in all mutant backgrounds. The *col-dAp-CRM* reports in the dAp cells (white dashed circles), in the sibling cells to the dAp cells (yellow dashed circles) and in one additional cell posterior to dAp sibling cells (magenta dashed circles) are all located in the same plane. (E) Quantification of GFP positive dAp cells in control and mutant backgrounds shows a significant reduction of GFP positive dAp cell numbers in all mutants when compared to control (_***_ p≤0.0001, n = 10 embryos, Students t-test, +/- SEM). (F-I) Stain for Eya and GFP in whole VNCs at stage AFT to determine the activity of (F) wild type *col-dAp-CRM* and (G-I) *col-dAp-CRMs* mutated for potential TFBS for *col-dAp* upstream activators (indicated by Δ), in the dAp cells. *col-dAp-CRMs* mutated for potential (G) Kr and (H) Pdm/POU sites, show a loss of GFP signal in the dAp cells. (I) Mutation of potential Grn/GATA binding sites, significantly reduces the GFP signal in the dAp cell. (J) Quantification of GFP positive dAp cells in embryos carrying the *col-dAp-CRM-ΔKr* or *col-dAp-CRM-ΔPOU/Pdm* constructs, shows a significant loss of GFP positive dAp cells when compared to control (_***_ p≤0.0001, n = 10 embryos, Students t-test, +/- SEM). (K) Quantification of GFP levels in dAp cells in flies carrying the *col-dAp-CRM-ΔGATA/Grn* constructs shows that the levels are significantly reduced when compared to the control (_***_ p≤0.0001, n≥30 cells, Students t-test, +/- SEM). Schematic representation of the *col* locus together with the position of the *col-dAp-CRM* fragment. Color coded dots represent potential transcription factor binding sites (TFBS) found in the *col-dAp-CRM* fragment, together with the conversion pattern, which was used to mutated the potential binding sites. Chr3:attP3 denotes the landing sites at which all, *col-dAp-CRM* as well as *col-dAp-CRM-*ΔTFBS constructs are inserted in the fly genome. Genotypes: (A) *col-dAp-CRM*. (B) *Kr*^*1*^, *Kr*^*CD*^*/Kr*^*1*^, *Kr*^*CD*^; *col-dAp-CRM/col-dAp-CRM*. (C) *Df(2L)ED773/ Df(2L)ED773; col-dAp-CRM/col-dAp-CRM (Df(2L)ED773* (removes both *nub* and *Pdm2*). (D) *col-dAp-CRM/col-dAp-CRM; grn*^*7L12*^/*grn*^*SPJ9*^. (F) *col-dAp-CRM*. (G) *col-dAp-CRM-*Δ*Kr*. *col-dAp-CRM-*Δ*POU/Pdm*. *Col-dAp-CRM-*Δ*Grn/GATA*.

Next we turned to the *col-dAp-CRM-GFP* enhancer and introduced it into the *Kr*, *pdm* and *grn* mutant backgrounds. We observed significant reduction in GFP expression in all three mutants, when compared to the enhancer transgene in a wild type background (control) stained on the same slide (Fig 4A–4E). The loss of GFP expression in the dAp cells, was accompanied by the loss of Eya expression. Next, we mutated all possible binding sites, conserved and non-conserved, for Kr, Pdm (POU-HD) and Grn (GATA) (Fig 4L; Materials and Methods; S3 Data and S4 Data). We integrated these mutant transgenes into the same genomic location as the wild type transgenic construct and assayed the expression of GFP expression in dAp neurons at stage AFT. We found that the enhancers mutated for Kr or Pdm displayed reduced number of dAp cells expressing GFP, when compared to the enhancer transgene in a wild type background (control) (Fig 4F–4H and 4J). The enhancer transgene mutated for Grn sites did not show a numerical loss of GFP expressing dAp cells, but did however show a significantly reduced level of expression in these cells (Fig 4I and 4K).

**Fig 5:**
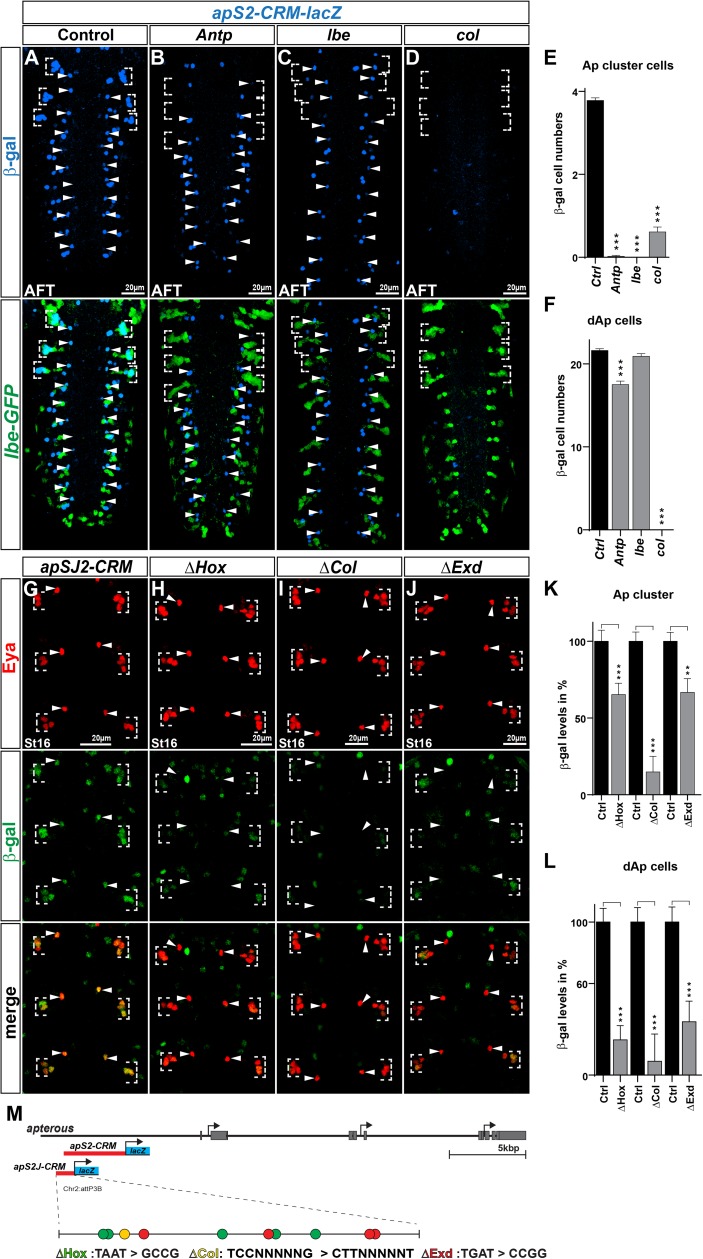
*apterous* enhancer analysis. (A-D) Whole *Drosophila* VNCs at stage AFT, stained for β-gal (reporter) and GFP (location) to show the activity of the *apS2-CRM* fragment under (A) control conditions and (B-D) in mutant backgrounds for transcription factors critical for *ap* activation i.e., *Antp*, *lbe* and *col*. *Antp*, *lbe* and *col* mutants result in loss of markers for the Ap cluster, therefore all experiments were performed with *lbe(K)-GFP* in the background, which allowed us to identify the NB5-6 lineage in T1-T3. (E) Quantification of β-gal positive Ap cluster cells in mutant background shows that all mutants reduce the enhancer activity significantly when compared to the control enhancer (_***_ p≤0.0001, n≥40 clusters, Students t-test, +/- SEM). (F) Quantification of β-gal positive dAp cells in the same mutants shows that *Antp* and *col* mutants significantly reduce the β-gal positive dAp cell numbers (_***_ p≤0.0001, n = 10 embryos, Students t-test, +/- SEM), whereas *lbe* mutants do not significantly reduce the β-gal positive dAp cell numbers. (G-J) Eya and β-gal staining of the shorter and Ap cell specific *apSJ2-CRM*-lacZ reporter construct and *apSJ2-CRMs* with mutated potential binding sites for (H) Hox (*Antp*, *lbe*), (I) Col and (J) Exd (indicated by Δ). (K) Quantification of β-gal levels in the Ap cluster cells, shows that mutation of potential TFBS, leads to a significant decrease of the *apSJ2-CRM* activity, when compared to control levels (_***_ p≤0.0001, _**_ p = 0.0002, n = 48 cells, Students t-test, +/- SEM). (L) Quantification of β-gal levels in the dAp cells, shows that the same mutations of potential TFBS, lead to a significant decrease of the *apSJ2-CRM* activity in dAp cells, when compared to control levels (_***_ p≤0.0001, n = 16 cells, Students t-test, +/- SEM). (N) Schematic representation of the *ap* locus together with the two enhancer fragments used. The longer *apS2-CRM*, was used for mutant background analysis and the shorter *apSJ2-CRM*, was used to generate mutated versions of the enhancer. Colored dots represent the potential TFBS in the *apSJ2-CRM* fragment together with the conversion pattern, used to mutate the indicated TBFS motifs. Genotypes: (A) *apS2-CRM-lacZ*. (B) *apS2-CRM-lacZ/lbe(K)-GFP*; *Antp*^*12*^*/Antp*^*25*^. (C) *apS2-CRM-lacZ/lbe(K)-GFP*; *lbe*^*12C5*^*/lbe*^*Df*^. (D) *col*^1^*/col*^3^; *apS2-CRM-lacZ/lbe(K)-GFP*. (G) *apSJ2-CRM-lacZ*. (H) *apSJ2-CRM-ΔHox*. (I) *apSJ2-CRM-*Δ*col*. (J) *apSJ2-CRM-Δexd*.

For analyzing the *ap* enhancer, we focused on the smaller *apS2J-CRM-lacZ* transgene, and placed this in the mutant background for *Antp*, *lbe* and *col*. Focusing on the Tv1 neurons, we observed significant reduction in β-gal expression in all three mutants (*Antp*, *lbe* and *col*), when compared to the enhancer transgene in a wild type background (control) stained on the same slide (Fig 5A–5E). As anticipated from the selective role of *Antp*, acting in the thorax, and *lbe*, in NB5-6T, the dAp neurons were only reduced in the thorax in *Antp*, and unaffected in *lbe* (Fig 5A–5D and 5F). In contrast, *col* mutants affected β-gal expression in both Tv1 and dAp neurons (Fig 5D–5F). Next, we mutated all conserved binding sites for Q50-Homeodomain proteins (TAAT; affecting both Antp and Lbe), Col and Exd (Fig 5M; Materials and Methods; S3 Data and S4 Data). We integrated these mutant transgenes into the same genomic location as the wild type transgenic construct and assayed the expression of β-gal expression in Tv1 and dAp neurons at stage 16. We found that all three mutated enhancers displayed reduced number of Tv1 and dAp cells expressing β-gal, when compared to the enhancer transgene in a wild type background (control) (Fig 5G–5L). We did not analyze the involvement of *hth* on the *ap-CRM* (or *eya-CRM*) because previous studies revealed that *hth* mutants could be fully rescued by re-expression of *col* [22]. *exd* mutants must be analyzed both as maternal and zygotic mutants, and we did not attempt to introduce the *ap-* and *eya-CRM* transgenes into such backgrounds.

Similar to the *ap* enhancer analysis, we placed the *eya-CRM-GFP* enhancer in the mutant background for *Antp*, *lbe* and *col*. Focusing on the Tv1 neurons, we observed significant reduction in GFP expression in all three mutants, when compared to the enhancer transgene in a wild type background (control) stained on the same slide (S4A–S4E Fig). As anticipated from the selective role of *Antp*, acting in the thorax, and *lbe* in NB5-6T, the dAp neurons were only reduced in the thorax in *Antp*, and unaffected in *lbe* (S4A–S4D and S4F Fig). In contrast, *col* mutants affected GFP expression in both Tv1 and dAp neurons (S4D–S4F Fig). Next, we mutated all conserved and non-conserved binding sites for Q50-Homeodomain proteins (TAAT; affecting both Antp and Lbe), Col and Exd (S4N Fig; Materials and Methods; S3 Data and S4 Data). We integrated these mutant transgenes into the same genomic location as the wild type transgenic construct and assayed the expression of GFP expression in Tv1 and dAp neurons at stage AFT. We found that the enhancers mutated for Hox and Col sites displayed reduced expression in both Tv1 and dAp cells, when compared to the enhancer transgene in a wild type background (control) (S4G–S4I, S4K and S4L Fig). In contrast, the mutation of Exd sites had no effect in Tv1 neurons, and surprisingly showed up-regulation in dAp neurons (S4J–S4L Fig).

For analyzing the *dimm* enhancer, we placed the *dimm-CRM-GFP* transgene in the mutant backgrounds for *Antp*, *ap*, *col* and *eya*. We observed significant reduction of GFP expression in both Tv1 and dAp neurons in all four mutants, when compared to the enhancer transgene in a wild type background (control) stained on the same slide (S4A–S4G Fig). Next, we mutated all conserved and non-conserved binding sites for Q50-Homeodomain proteins (TAAT; affecting both Ap and Antp), Col and Exd (S5O Fig; Materials and Methods; S3 Data and S4 Data). We integrated these mutant transgenes into the same genomic location as the wild type transgenic construct and assayed the expression of GFP expression in Tv1 and dAp neurons at stage AFT. We found that enhancer mutants for Hox and Exd displayed reduced GFP expression in both Tv1 and dAp cells, when compared to the enhancer transgene in a wild type background (control) (S5H–S5N Fig). In contrast, the Col mutant enhancer showed slightly elevated expression in Tv1 neurons while expression was reduced in dAp neurons (S5J and S5L–S5N Fig).

**Fig 6:**
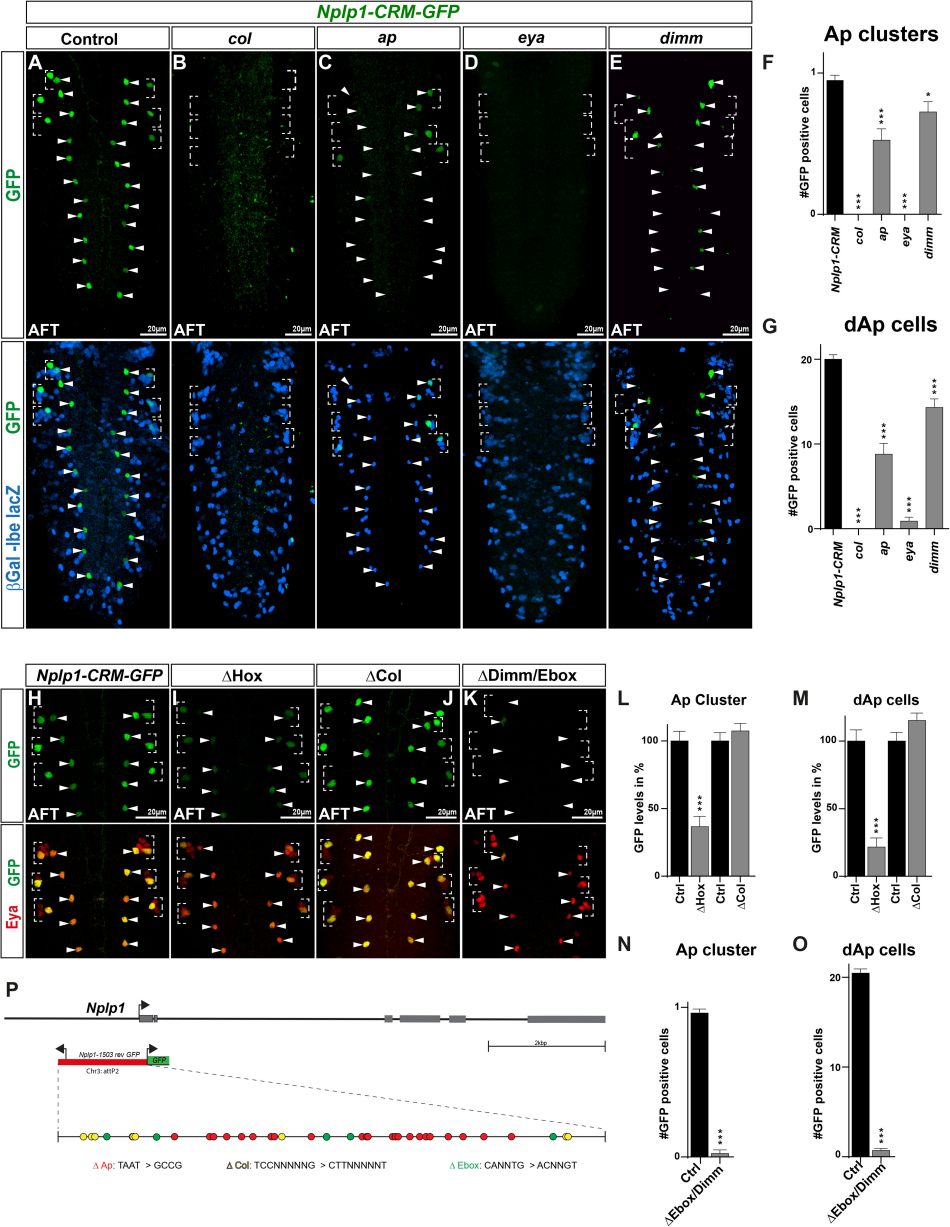
*Nplp1* enhancer analysis. (A-E) *Drosophila* VNCs at stage AFT showing the *Nplp1-CRM-GFP* reporter expression, stained for *lbe(K)-lacZ*/β-gal (location) and GFP (reporter) to show the activity of the *Nplp1-CRM-GFP* construct under (A) control conditions and (B-E) in different mutant backgrounds for transcription factors critical for *Nplp1* activation i.e., *col*, *ap*, *eya* and *dimm*. Because the different upstream mutants result in loss of markers for the Ap cluster, all experiments were performed with the *lbe(K)-lacZ* construct in the background, which allows to identify the NB5-6 lineage in T1-T3. (F) Quantification of GFP positive Ap cluster cells in different mutant backgrounds shows that all mutants reduce the enhancer activity significantly when compared to the control enhancer (_***_ p≤0.0001, _*_ p = 0.031; n≥40 clusters, Students t-test, +/- SEM). (G) Quantification of GFP positive dAp cells in different mutant backgrounds shows that all mutants reduce the GFP positive dAp cell numbers significantly (_***_ p≤0.0001, n = 10 embryos, Students t-test, +/- SEM). (H-K) Eya and GFP staining of the *Nplp1-CRM* construct with mutated potential binding sites for Hox (*ap*), Col and Dimm/E-box (indicated by Δ) at stage AFT. (L) Quantification of GFP levels in the Ap cluster cells, shows that single factor binding site mutation for Hox binding sites leads to a significant decrease of the *Nplp1-CRM* activity, when compared to control levels. Mutation of potential Col binding sites results in a slight but not significant increase in *Nplp1-CRM* activity (_***_ p≤0.0001, n≥36 cells, Students t-test, +/- SEM). (M) Quantification of GFP levels in the dAp cells, shows that single factor binding site mutation of potential Hox binding sites leads to a significant decrease of the *Nplp1-CRM* activity in dAp cells, when compared to control levels, while mutation of potential Col bindings sites resulted in a slight but non-significant increase in *Nplp1-CRM* activity (_***_ p≤0.0001, n≥ 30 cells, Students t-test, +/- SEM). (N) Quantification of GFP positive Ap cluster cell numbers shows that mutation of potential Dimm/E-box binding sites in the *Nplp1-CRM*, leads to significant reduction of GFP positive Ap cluster cells compared to control numbers (_***_ p≤0.0001, n≥40 clusters, Chi-square test, +/- SEM). (O) Quantification of GFP positive dAp cell numbers shows that mutation of potential Dimm/E-box binding sites in the *Nplp1-CRM*, leads to significant reduction of GFP positive dAp cells when compared to control numbers (_***_ p≤0.0001, n = 10 embryos, Student´s t-test, +/- SEM). (P) Schematic representation of the *Nplp1* locus together with the enhancer-reporter construct. Colored dots represent the potential TFBS in the *Nplp1-CRM* fragment together with the conversion pattern, used to mutate the indicated TBFS motifs. Genotypes: (A) *Nplp1-CRM-GFP/lbe(K)-lacZ*. (B) *col*^*1*^*/col*^*3*^*; Nplp1-CRM-GFP/lbe(K)-lacZ*. (C) *ap*^*p44*^*/ap*^*p44*^*; Nplp1-CRM-GFP/lbe(K)-lacZ*. (D) *eya*^*Df*^*/eya*^*Cli*^*; Nplp1-CRM-GFP/lbe(K)-lacZ*. (E) *dimm*^*rev4*^*/dimm*^*P1*^*; Nplp1-CRM-GFP/lbe(K)-lacZ*. (H) *Nplp1-CRM-GFP*. (I) *Nplp1-CRM-ΔHox*. (J) *Nplp1-CRM-ΔCol*. (K) *Nplp1-CRM-ΔDimm/E-box*.

For analyzing the *Nplp1* enhancer, we placed the *Nplp1-CRM-GFP* transgene in the mutant backgrounds for *col*, *ap*, *eya* and *dimm*. We observed significant reduction of GFP expression in all four mutants, when compared to the enhancer transgene in a wild type background (control) stained on the same slide (Fig 6A–6G). Next, we mutated all possible conserved and non-conserved binding sites for Q50-Homeodomain proteins (TAAT; affecting Ap), Col and Dimm (E-boxes) (Fig 6P; Materials and Methods; S3 Data and S4 Data). We integrated these mutant transgenes into the same genomic location as the wild type transgenic construct and assayed the expression of GFP expression in Tv1 and dAp neurons at stage AFT. We observed that the enhancers mutated for Hox or Dimm sites displayed reduced GFP expression in both Tv1 and dAp cells, when compared to the enhancer transgene in a wild type background (control) (Fig 6H, 6I and 6K–6O). In contrast, the enhancer mutated for Col sites did not display any effect on GFP expression (Fig 6J, 6L and 6M).

### Misexpression of terminal selectors can activate the enhancers

**Fig 7:**
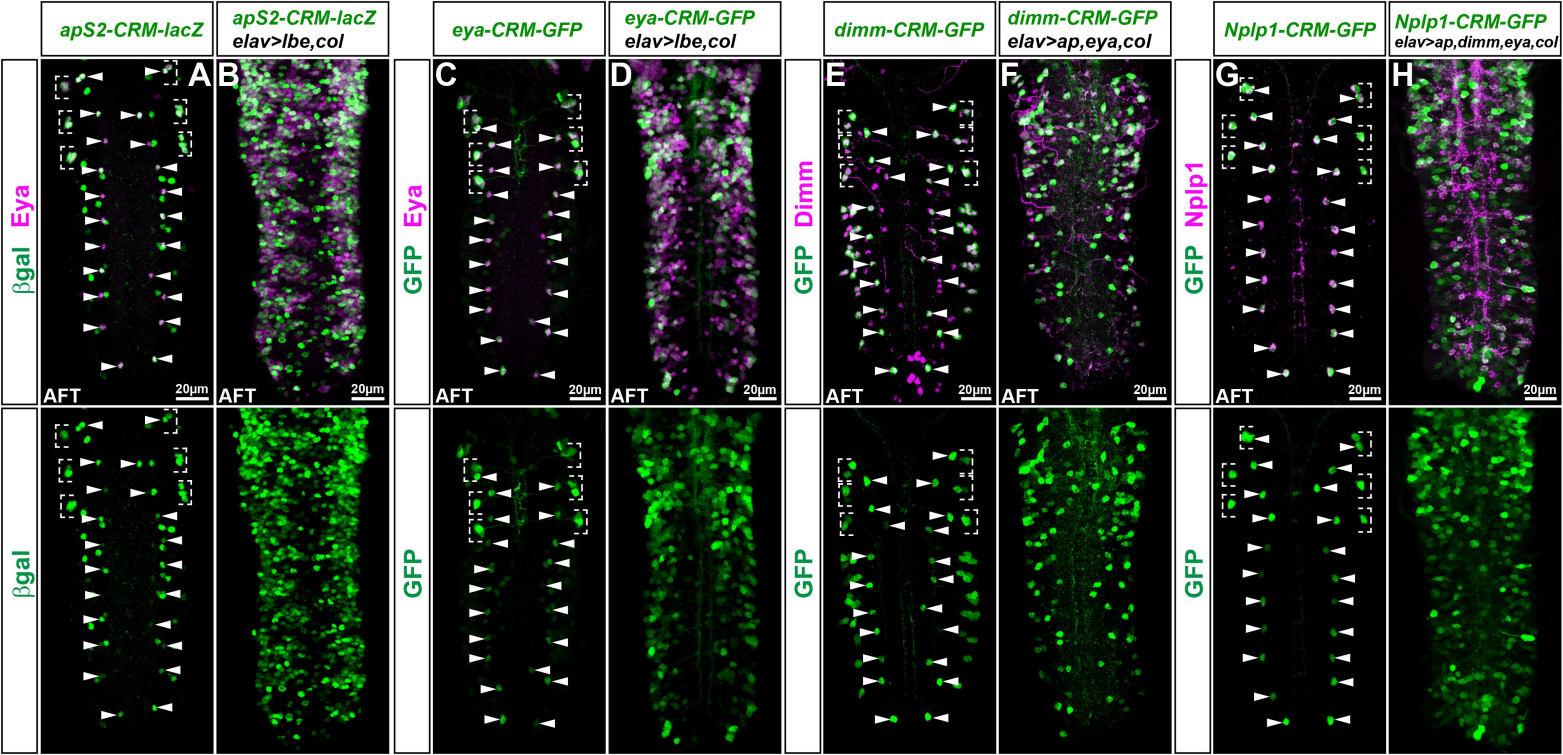
The FFL enhancers can be activated by misexpression. (A-H) Expression of the separate CRM-reporter constructs under control and misexpression conditions from *elav-Gal4* at stage AFT. (A-B) Staining for Eya and β-gal of the *apS2-CRM-lacZ* transgene in (A) control and (B) in a *UAS-col*, *UAS-lbe* co-misexpression background, which results in broad activation of the *apS2-CRM*-*reporter* construct. (C-D) Staining for Eya and GFP of the *eya-CRM-GFP* transgene under (C) control condition and (D) in *UAS-col*, *UAS-lbe* co-misexpression background, which results in broad activation of the *eya-CRM* reporter construct. (E-F) Staining for Dimm and GFP of the *dimm-CRM-GFP* transgene under (E) control condition and (F) in *UAS-col*, *UAS-ap*, *UAS-eya* “dimm-cocktail” misexpression background, which results in broad activation of the *dimm-CRM-GFP* reporter construct. (G-H) Staining for Nplp1 and GFP of the *Nplp1-CRM-GFP* transgene under (G) control condition and (H) in *UAS-col*, *UAS-ap*, *UAS-eya*, *UAS-dimm “*Nplp1-cocktail*”* misexpression background, which results in broad activation of the *Nplp1-CRM-GFP* reporter construct. Genotypes: (A) *apS2-CRM-lacZ*. (B) *elav-Gal4/+;; apS2-CRM-lacZ/UAS-lbe*, *UAS-col*. (C) *eya-CRM-GFP*. (D) *elav-Gal4/+;; eya-CRM-GFP/UAS-lbe*, *UAS-col*. (E) *dimm-CRM-GFP*. (F) *elav-Gal4/+;UAS-ap/+; dimm-CRM-GFP/UAS-eya*, *UAS-col*. (G) *Nplp1-CRM-GFP*. (H) *elav-Gal4/+;UAS-ap*, *UAS-dimm/+; Nplp1-CRM-GFP/UAS-eya*, *UAS-col*.

Previous studies have revealed that combinatorial misexpression of the transcription factors in the Tv1/dAp cascade is able to broadly activate the genes in the FFL [6, 18, 21, 29, 30]. To determine if such combinatorial ectopic effects could act upon the identified enhancers taken out of genomic context, we misexpressed various combinatorial codes of TFs and studied the effects on the pertinent transgenes. Focusing on the *apS2-CRM-lacZ* and *eya-CRM-GFP*, we find broad activation of both transgenes when *lbe* and *col* are co-misexpressed (Fig 7A–7D). Similarly, combinatorial misexpression of *ap*, *eya* and *col* could ectopically activate the *dimm-CRM-GFP* transgene (Fig 7E and 7F). Finally, the *Nplp1-CRM-GFP* transgene was broadly activated by combinatorial expression of *ap*, *dimm*, *eya* and *col* (Fig 7G and 7H). In all cases, as anticipated, we observed up-regulation of the endogenous Eya, Dimm and Nplp1 proteins (Fig 7A–7H). These results demonstrate that ectopic activation of the dAp/Tv1 transcriptional program can robustly act upon the identified enhancers even outside their normal genomic context.

## Discussion

**Fig 8:**
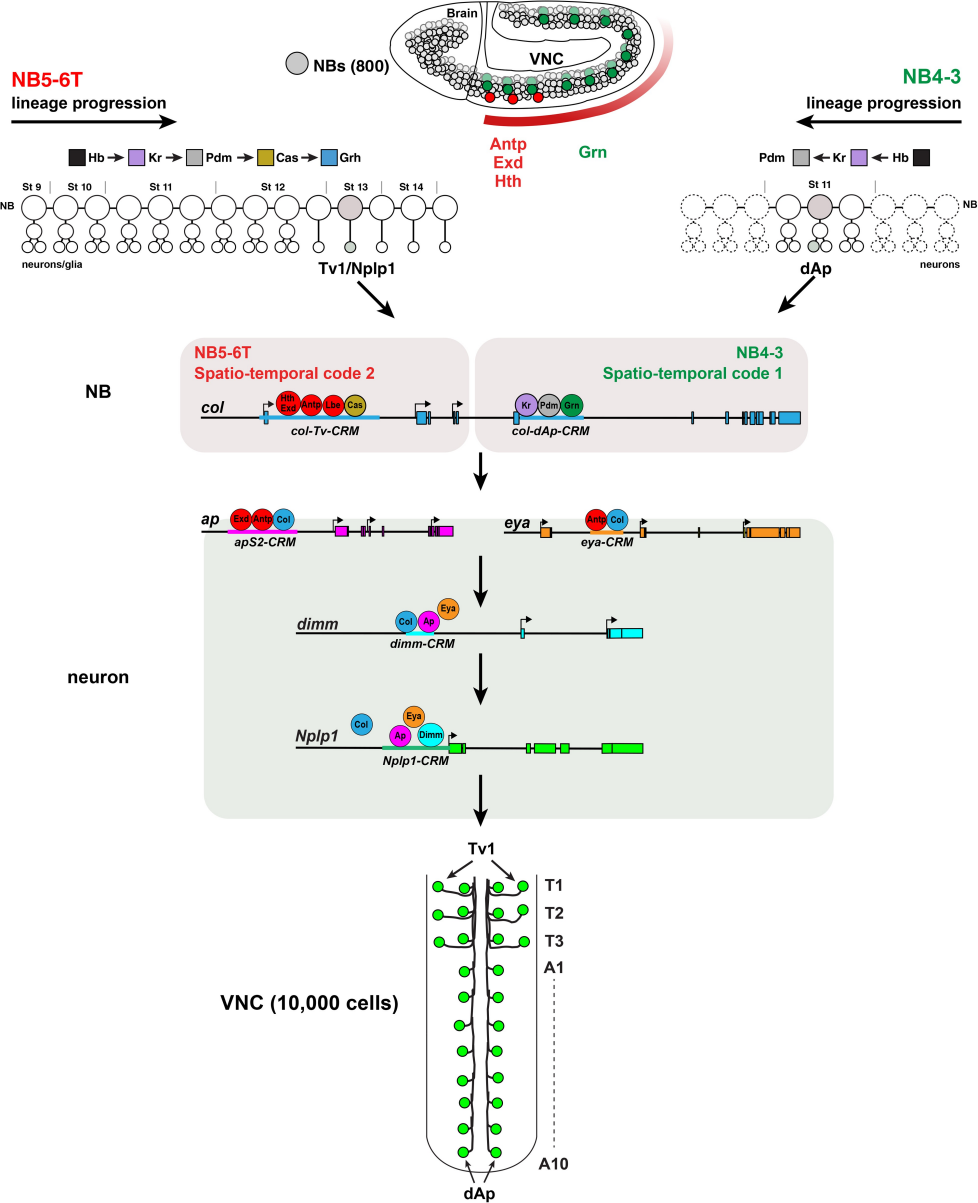
Model Cartoon. Summary of the identified genetic and molecular pathways controlling specification of the Tv1 and dAp neurons. See text for details.

Combining the findings presented in this study, we have been able to molecularly decode the Tv1/dAp genetic FFL cascades [6, 20, 21, 30], bolstering evidence for a complex molecular FFL, based upon sequential transcription factor binding to the downstream genes (Figs 8 and S6). The NB4-3 and NB5-6T neuroblasts are born in different regions of the VNC, and express different spatial determinants i.e., Antp, Lbe, Hth, Exd and Grn [20–22]. As lineage progression commences, they undergo a programmed cascade of transcription factor expression; the temporal cascade [9]. Early temporal factors Kr and Pdm integrate with Grn in NB4-3, while the late temporal factor Cas integrates with Antp, Lbe, Hth and Exd in NB5-6T, to create two distinct combinatorial spatio-temporal codes. These two codes converge on two different enhancers in the *col* gene, triggering Col expression, and hence the Nplp1 FFL. The FFL, in this case a so-called coherent FFL, where regulators act positively at one or several steps of a cascade, was first identified in *E*.*coli* and yeast regulatory networks [31], but have also been identified in *C*.*elegans* and *Drosophila* [6, 32, 33]. Coherent FFLs can act as regulatory timing devices, exemplified by the action of *col* in NB5-6T: The initial expression of *col* in Ap cluster cells triggers a generic Ap/Eya interneuron fate in all four cells, while its downregulation in Tv2-4 and maintenance in Tv1 helps propagate the FFL leading to Nplp1 expression [6, 20, 21, 30].

### Spatio-temporal convergence on *collier*, an initiator terminal selector gene

We find that the two different spatio-temporal programs converge on *col*, but on different enhancer elements. However, neither enhancer element gave complete null effects when deleted. Specifically, the 6.3kb *col*-*Tv-CRM* shows robust reporter expression, overlaps with endogenous *col* expression, responds to the upstream mutants, and is affected by TFBS mutations. However, when deleted (generating the *col*^*ΔTv-CRM*^ mutant), it had weak effects upon endogenous *col* expression in NB5-6T, and no effect upon Eya and Nplp1 expression. Deletion of the *col-dAp-CRM* (generating the *col*^*ΔdAp-CRM*^ mutant), gave more robust effects with reduction of Col, Eya and Nplp1 in dAp cells, although the expression was not lost completely.

Early developmental genes, which often are dynamically expressed, may be controlled by multiple enhancer modules, to thereby ensure robust onset of gene expression. This has been reported previously in studies of early mesodermal and neuro-ectodermal development, in which several genes i.e., *twist*, *sog*, *snail* are controlled by multiple distal enhancer fragments, so called “shadow enhancers”, in order to ensure reliable onset of gene expression [25]. The shadow enhancer principle is also supported by recent findings on the *Kr* gene [27]. Moreover, extensive CRM transgenic analysis, scoring thousands of fragments in transgenic flies, has also supported the shadow enhancer idea, revealing that a number of early regulators, several of which encode for transcription factors, indeed have shadow enhancers [26]. The dichotomy between the *col* transgenic reporter results and the partial impact on *col* expression upon deletion of its Tv1 and dAp enhancers, gives reason to speculate that *col* may be under control of additional enhancers, some of which may be referred to as shadow enhancers.

The results on the *eya*, *ap*, *dimm* and *Nplp1* enhancer mutants stand in stark contrast to the *col* CRMs findings. For these four genes, the enhancer deletion resulted in robust, near null effects, on expression. It is tempting to speculate that our findings, combined with previous studies, points to a different logic for early regulators, with highly dynamic patterns, requiring several functionally overlapping enhancers for fidelity, and late regulators and terminal differentiation genes, which may operate with one enhancer that is inactive until the pertinent combinatorial TF codes have been established.

### Action of *collier*

Analysis of the *ap* and *eya* enhancers indicates that Col directly interacts with these enhancers. Both of these enhancer-reporter transgenes are affected in *col* mutants, and can be activated by ectopic *col*. Moreover, mutation of one Col binding site in the *ap* enhancer and two sites in the *eya* enhancer, was enough to dramatically reduce enhancer activity. Direct action of Col on *ap* and *eya* is furthermore supported by recent data on Col genome-wide binding, using ChIP, which demonstrated direct binding of Col to these regions of *ap* and *eya* in the embryo [34]. The regulation of *ap* is an excellent example of the complexity of gene regulation, and studies have identified additional enhancers controlling *ap* expression in the wing, muscle and brain [35–38].

In contrast to regulation of *ap* and *eya*, a direct action of Col on *dimm* and *Nplp1* is less clear. Analysis of the *dimm* and *Nplp1* enhancers did not reveal perfectly conserved Col binding sites. Mutation of multiple non-perfect Col binding sites in the *dimm* enhancer did not affect reporter expression in the Ap cluster, but did however reduce levels in the dorsal Ap cells. Mutation of non-perfect Col binding sites in the *Nplp1* enhancer had no impact on enhancer activity, neither in Tv1 nor dAp. These findings support a model where Col is crucial for directly activating *ap* and *eya*, which in turn directly activate *dimm* and *Nplp1*, with some involvement of Col on *dimm* (Fig 8). However, support for a direct role for Col on *Nplp1* comes from RNAi studies in larvae or adult flies, showing that knockdown of *col* resulted in loss of Nplp1, while Ap, Eya and Dimm expression was unaffected [6, 39].

It is tempting to speculate that Col regulates *Nplp1* not via direct interaction with its enhancer, but rather as a chromatin state modulator, keeping the chromatin around the *Nplp1* locus in an accessible state, in order for Dimm, Ap and Eya to be able to access the *Nplp1* gene. Support for this notion comes from studies on the mammalian Col orthologue EBF, which is connected to the chromatin remodeling complex SWI/SNF during EBF-mediated gene regulation in lymphocytes [40]. Moreover, the central SWI/SNF component Brahma was recently identified in a genetic screen for Ap cluster neurons, and found to affect FMRFa neuropeptide expression in Tv4 without affecting Eya expression, indicating a late role in Ap cluster differentiation [41]. Alternatively, Col may activate Nplp1 via unidentified, low affinity sites, similar to the mechanism by which *Ubx* regulates some of its embryonic target genes [42].

### Downstream of *collier*

Col activates the *ap* and *eya* genes. *ap* encodes a LIM-HD protein, a family of transcription factors well known to control multiple aspects of terminal neuronal subtype fate, including neurotransmitter identity, axon pathfinding and ion channel expression [17, 43–45]. Our results indicate that Ap in turn acts upon *dimm*, and subsequently with Dimm on Nplp1. *eya* encodes an evolutionary well-conserved phosphatase and does not bind DNA directly, instead acting as a transcriptional co-factor [46]. Eya (and its orthologues) have been found to interact with several transcription factors in different systems [46], but whether it forms complexes with Col and Ap is not known.

The final transcription factor in the FFL is Dimm, a bHLH protein. Dimm is selectively expressed by the majority of neuropeptide neurons in *Drosophila*, and is important for expression of many neuropeptides [6, 19, 29, 47, 48]. Intriguingly, Dimm is also both necessary and sufficient to establish the dense-core secretory machinery, found in neuropeptide neurons [29, 48–52]. Based upon these findings Dimm has been viewed as a cell type selector gene [1] or a “scaling factor” [53], acting to up-regulate the secretory machinery. Here, we find evidence for that Dimm acts directly on the *Nplp1* enhancer, and this raises the possibility that Dimm is both a selector gene for the dense-core secretory machinery, and can act in some neuropeptide neurons to directly regulate specific neuropeptide gene expression.

## Materials and methods

### Fly stocks

Location marker lines: *lbe(K)-EGFP* [54]. *lbe(K)-lacZ* (provided by K. Jagla)[55].

Mutant lines: *lbe*^*12C005*^ (BL#59385). *Df(lbl-lbe)B44* (provided by K. Jagla). *Antp*^*12*^ [56](provided by F. Hirth). *Antp*^*25*^ (BL#3020). *cas*^*Δ1*^ and *cas*^*Δ3*^ [57] (provided by W. Odenwald). *col*^*1*^, *col*^*3*^ [58] [59] (provided by A. Vincent). *hth*^*5E04*^ (BL#4221). *Df(3R)Exel6158* (BL#7637; referred to as *hth*^*Df7637*^). *Df(2L)BSC354 (referred to as eya*^*Df*^*)* (BL#24378). *eya*^*cli-IID*^ (BL#3280). *dimm*^*rev4*^ and *dimm*^*P1*^ (provided by Douglas W. Allan). *ap*^*P44*^ [60].

Misexpression lines: *UAS-col* [59] (provided by A. Vincent). *UAS-col-HA* and *UAS-myc-lbe* [30]. *UAS-dimm* [47]. *UAS-eya* (BL#5675). *UAS-ap* [61].

Driver lines: *elav*^*C155*^ = *elav-Gal4* (BL#458).

CRM lines: *col-dAp-CRM* (chr.2:28E7; chr.3:68A4). *col-Tv-CRM* (chr2:25C7). *apS2-CRM (*chr2:22A3; chr.3:62E1). *apSJ2-CRM* (chr2:28E7). *eya-CRM* (chr.2:28E7; chr.3:68A4). *dimm-CRM* (chr.2:28E7; chr.3:68A4). *Nplp1-CRM* (chr.2:28E7; chr.3:68A4).

CRM mutant lines: *col*^*ΔTv-CRM*^, *col*^*ΔdAp-CRM*^, *eya*^*ΔCRM*^, *ap*^*ΔapS-CRM*^, *dimm*^*ΔCRM*^, *Nplp1*^*ΔCRM*^.

gRNA-lines: *vas-Cas9* (BL#51323). *ap*^*ΔapS-CRM*^ gRNAs (chr.3: 68A4). *col*^*ΔdAp-CRM*^ gRNAs (chr.2: 28E7). *col*^*ΔTv-CRM*^ gRNAs (chr.3 68A4). *eya*^*ΔCRM*^ gRNAs (chr.2:28E7). *dimm*^*ΔCRM*^ gRNAs (chr.2: 28E7). *Nplp1*^*ΔCRM*^ gRNAs (chr.2: 28E7).

Mutants were maintained over *GFP*- or *YFP*-marked balancer chromosomes. As wild-type control *OregonR* was used. Staging of embryos was performed according to Campos-Ortega and Hartenstein [62].

### Immunohistochemistry

Primary antibodies were: Guinea pig α-Deadpan (1:1,000) and rat α-Dpn (1:200) [54]. Guinea pig α-Col (1:1,000), guinea pig α-Dimm (1:1,000), chicken α-proNplp1 (1:1000) [6]. Rat α-Nab (1:500) [20]. mAb α-Eya 10H6 (1:250) (Developmental Studies Hybridoma Bank, Iowa City, IA, US). Rabbit α-Ap (1:1,000) [35] (provided by D. Bieli and M. Affolter). Chicken α-GFP 1:1,000 (Abcam, ab13970).

### Transgenic enhancer flies

In brief; all wild-type enhancers, were either PCR amplified (Expand High Fidelity^Plus^ PCR system, from Roche Diagnostics (Indianapolis, IN, USA) from the *OregonR* DNA, or de-novo synthetized at GenScript Inc. (Piscataway, NJ, USA) in the case of the *col-Tv-CRM* enhancer and all other mutant enhancer versions. PCR amplified DNA fragments were cloned into the pCR2.1-TOPO® TA vector according to the manufactures protocol (Invitrogen Life technologies, Carlsbad, CA, USA) for further cloning steps into the placZ.attB or pEGFP.attB landing site vectors [63] (provided by K. Basler and J. Bischof). Furthermore TOPO clones containing the wild type enhancer sequences were sent to GATC Biotech AG (Cologne, Germany) for Sanger sequencing. All synthesized enhancer constructs were delivered in a pUC57 vector and subsequently cloned either into the placZ.attB or pEGFP.attB landing site vectors, and integrated into the fly genome via site directed phiC31 mediated integration [64] at BestGene Inc (Chino Hills, CA, USA) or GenetiVision (Houston, TX, USA).

### CRISPR/Cas9

The online tool (http://tools.flycrispr.molbio.wisc.edu/targetFinder/) was used to design two protospacers with zero predicted off-targets for each CRM, flanking the 5`and 3`regions of the identified enhancer constructs. Sequences for all protospacers can be found in the supplemental information (CRISPR). Primer design and vector assembly was done according to the protocol found at http://www.crisprflydesign.org/wp-content/uploads/2014/06/Cloning-with-pCFD4.pdf. PCR was performed using the Expand High Fidelity^Plus^ PCR system (Roche Diagnostics, Indianapolis, IN, USA) according to the provided protocol with an annealing temperature of +61°C. In order to delete the CRMs identified in this study, the tandem gRNA vector (pCFD4-U6:1_U6:3) (Addgene # 49411; gift from Simon Bullock) was used to express two gRNAs simultaneously, which flank the 5´ and 3´ regions of the CRMs. The empty vector served as a template during PCR amplification to introduce the protospacers into the gRNA core sequence and U6-1 and U6-3 promoter regions. PCR products containing the protospacers were cloned into the tandem gRNA vector by ligation independent cloning using Gibson Assembly according to the manufacturers’ protocol (New England Biolabs Inc., Ipswich, MA, USA). All gRNA vector constructs were Sanger sequenced by use of the M13 for and M13 reverse primers to confirm for the correct insert (GATC Biotech AG, Cologne, Germany). Stable transgenic gRNA flies were generated at BestGene and tandem gRNA constructs containing attB landing sites were landed via phi31 mediated integration on the second or third chromosome on cytolocation 28E7 and 68A4. Fly stocks mutant for CRMs were created by crossing males of the transgenic tandem gRNA flies to virgins of *vas-Cas9* (BL#51323). Stable stocks mutant for CRMs were tested by PCR by using PCR primers flanking the deleted region. PCR fragments spanning the deleted region were sequenced to confirm deletion (Supplemental Information CRISPR).

### Confocal imaging and data acquisition

Zeiss LSM 700 Confocal microscopes were used for fluorescent images; confocal stacks were merged using LSM software or Adobe Photoshop. Statistic calculations were performed in Graphpad prism software (v4.03). Cell counts and reporter (GFP or β-gal) measurements were done with ImageJ FIJI and numbers transferred to Graphpad prism. To address statistical significance Student's *t-*test or in the case of invariant cell numbers contingency tables together with Chi-Square test were used. Images and graphs were compiled in Adobe Illustrator.

## Supporting information

S1 Fig Related to Fig 1. **Enhancer Identification.** Genomic outline of the genes in the Nplp1 FFL cascade, and the various DNA fragments tested in transgenic lines (under each gene).
(EPS)

S2 Fig Related to Fig 2 CRISPR/Cas9 deletion of enhancers affects Tv1 and dAp specification. (A-C) Staining for Eya, Col and Nplp1 in the Ap cluster at stage AFT in control, *col*^*ΔTv-CRM*^ and *col*^*ΔdAp-CRM*^, shows that Eya, Col and Nplp1 expression is not affected in the Ap cluster by mutating either of the *col* CRMs. (D-F) Staining for Eya and Col in whole VNCs at stage AFT, in control, *col*^*ΔTv-CRM*^ and *col*^*ΔdAp-CRM*^, shows that only the *col*^*ΔdAp-CRM*^ mutant affects Col and Eya expression in dAp cells. (G) Quantification of Col positive Ap cluster cells in control, *col*^*ΔTv-CRM*^ and *col*^*ΔdAp-CRM*^ mutants shows that neither mutant affects Col expression (p = n.s., n = 60 clusters, Students t-test, +/- SEM). (H) Quantification of Col positive dAp cells in control, *col*^*ΔTv-CRM*^ and *col*^*ΔdAp-CRM*^ mutants shows that the numbers of Col positive dAp cells is only significantly reduced in *col*^*ΔdAp-CRM*^ mutants (_***_ p≤0.0001, n = 10 embryos, Students t-test, +/- SEM). (I-K) Staining of Eya, Ap and Nplp1 in the Ap cluster at stage AFT in control, *eya*^*ΔCRM*^ and *ap*^*ΔapS-CRM*^ mutants. In *eya*^*ΔCRM*^ mutants, Eya expression is lost in some cells in the Ap cluster, while Ap expression is weakly affected. In *ap*^*ΔapS-CRM*^ mutants, Eya expression is unaffected while Ap expression is lost. In both mutants, *Nplp1* expression is lost from the Ap cluster. (L-N) Staining of Eya, Ap and Nplp1 in the dAp cells at stage AFT in control, *eya*^*ΔCRM*^ and *ap*^*ΔapS-CRM*^ mutants. In *eya*^*ΔCRM*^ mutants, Eya expression is lost from the dAp cells, while Ap is still expressed. In *ap*^*ΔapS-CRM*^ mutants, Eya is most often expressed, while *ap* is completely lost from the dAp cells. (O-P) Quantification of Ap positive cells in the Ap cluster and dAp cells, in *eya*^*ΔCRM*^ and *ap*^*ΔapS-CRM*^ mutants. (O) Numbers of Ap positive cells are weakly, but significantly, reduced in Ap clusters in *eya*^*ΔCRM*^ mutants and strongly reduced in *ap*^*ΔapS-CRM*^ mutants (_***_ p≤0.0001, n = 44 clusters, Students t-test, +/- SEM). (P) Numbers of Ap positive dAp cells are slightly but significantly reduced in *eya*^*ΔCRM*^ mutants and completely absent in *ap*^*ΔapS-CRM*^ mutants (_***_ p≤0.0001, n = 10 embryos, Students t-test, +/- SEM). (Q-S) Staining for Eya, Dimm and Nplp1 in the Ap cluster at stage AFT in control, *dimm*^*ΔCRM*^, and *Nplp1*^*ΔCRM*^ mutants. In *dimm*^*ΔCRM*^ mutants, Dimm and Nplp1 expression is absent from the Ap cluster. In *Nplp1*^*ΔCRM*^ mutants, Dimm expression is not affected, but *Nplp1* expression is lost. (T-V) Staining for Eya, Dimm and Nplp1 in the dAp cells at stage AFT in control, *dimm*^*ΔCRM*^, and *Nplp1*^*ΔCRM*^ mutants. In *dimm*^*ΔCRM*^, mutants Dimm and Nplp1 expression is absent in dAp cells. In *Nplp1*^*ΔCRM*^ mutants, Dimm expression is unaffected, while Nplp1 expression is lost. (W-X) Quantification of Dimm cell numbers in the Ap cluster and dAp cells in *dimm*^*ΔCRM*^, and *Nplp1*^*ΔCRM*^ mutants. In both Ap clusters and dAp cells, Dimm cell numbers are significantly reduced in *dimm*^*ΔCRM*^ mutants, while *Nplp1*^*ΔCRM*^ mutants have no effect (_***_ p≤0.0001, n≥56 clusters (W), n = 10 embryos (X); Students t-test, +/- SEM).
Genotypes: (A, D, I, L, Q, T) *OregonR*. (B, E) *col*^*ΔTv-CRM*^. (C, F) *col*^*ΔdAp-CRM*^. (J, M) *eya*^*ΔCRM*^. (K, N) *ap*^*ΔapS-CRM*^. (R, U) *dimm*^*ΔCRM*^. (S, V) *Nplp1*^*ΔCRM*^.
(EPS)

S3 Fig Related to Fig 2 CRISPR/Cas9 deletion of enhancers affects Tv1 and dAp specification. (A-B) Staining for Eya, Ap and Col in the Ap cluster at stage AFT, in (A) control and (B) *eya*^*ΔCRM*^ mutants. At AFT, Col specifically marks the Tv1 cells, in both (A) control and (B) *eya*^*ΔCRM*^. (B) In *eya*^*ΔCRM*^ mutant embryos, Eya expression is lost in two out of four Ap cluster cells, shown by co-staining with Apterous, and specifically lost from the Tv1 cell, shown by co-stain with Col.
Genotypes: (A) *OregonR*. (B) *eya*^*ΔCRM*^.
(EPS)

S4 Fig Related to Fig 5 *eya* enhancer analysis. (A-D) *Drosophila* VNC at stage AFT, stained for β-gal (location) and GFP (reporter) to show the activity of the *eya-CRM* fragment driving GFP under (A) control conditions and (B-D) in mutant background for transcription factors critical for *eya* activation i.e. *Antp*, *lbe* and *col*. Since *Antp*, *lbe* and *col* mutants result in loss of markers for the Ap cluster, all experiments were performed in with the *lbe(K)-lacZ* construct in the background, which allows to identify the NB5-6 lineage in T1-T3. (E) Quantification of GFP positive Ap cluster cell numbers in mutant backgrounds shows that all mutants reduce the enhancer activity significantly when compared to the control enhancer (_***_ p≤0.0001, n≥40 clusters, Students t-test, +/- SEM). (F) Quantification of GFP positive dAp cell numbers in mutant backgrounds shows that *Antp* and *col* mutants significantly reduce the GFP positive dAp cell numbers (_***_ p≤0.0001, _**_ p = 0.003 n = 10 embryos, Students t-test, +/- SEM) whereas *lbe* mutants do not reduce the GFP positive dAp cell numbers significantly. (G-J) Eya and GFP staining for the *eya-CRMs* with mutated potential binding sites for (H) Hox (*Antp*, *lbe*), (I) Col and (J) Exd (indicated by Δ). (K) Quantification of GFP levels in the Ap cluster cells, shows that mutations of potential Hox and Col TFBS leads to a significant decrease of the *eya-CRM-GFP* activity when compared to control levels (_***_ p≤0.0001, n≥ 32 cells, Students t-test, +/- SEM). Mutation of potential Exd binding sites, showed a slight, but not significant increase in GFP levels in the Ap cluster cells, when compared to control levels. (L) Quantification of GFP levels in the dAp cells, shows that mutation of potential Hox TFBS sites significantly decreases the *eya-CRM-GFP* activity in dAp cells, when compared to control levels. Mutation of potential Exd TFBS resulted in significantly increased GFP levels compared to control (_***_ p≤0.0001, n = 30 cells, Students t-test, +/- SEM). (M) Quantification of GFP positive dAp cell numbers, shows that mutation of potential Col TFBS, results in a complete loss of eya*-CRM* activity in dAp cells (_***_ p≤0.0001, n = 10 embryos, Students t-test, +/- SEM). (N) Schematic representation of the *eya* locus together with the *eya-CRM-GFP* reporter construct. Colored dots represent the potential TFBS in the *eya-CRM* fragment together with the conversion pattern, used to mutate the indicated TBFS motifs.
Genotypes: (A) *eya-CRM-GFP*. (B) *eya-CRM-GFP/+*; *Antp*^*12*^, *lbe(K)-lacZ/Antp*^*25*^. (C) *eya-CRM-GFP/lbe(K)-lacZ*; *lbe*^*12C5*^*/lbe*^*Df*^. (D) *col*^1^*/col*^3^; *eya-CRM-GFP/lbe(K)-lacZ*. (G) *eya-CRM-GFP*. (H) *eya-CRM-*Δ*Hox*. (I) *eya-CRM-*Δ*Col*. (J) *eya-CRM-*Δ*Exd*.
(EPS)

S5 Fig Related to Fig 5 *dimm* enhancer analysis. (A-E) *Drosophila* VNCs at stage AFT showing the *dimm-CRM* reporter expression, stained for *lbe(K)-lacZ*/β-gal (location) and GFP (reporter) to show the activity of the *dimm-CRM* fragment under (A) control conditions and (B-E) in different mutant backgrounds for transcription factors critical for *dimm* activation i.e., *Antp*, *ap*, *eya* and *col*. Because the different upstream mutants result in loss of markers for the Ap cluster, all experiments were performed in with the *lbe(K)-lacZ* construct in the background, which allowed us to identify the NB5-6 lineage in T1-T3. (F) Quantification of GFP positive Ap cluster cells in different mutants shows that all mutants significantly reduce the enhancer activity when compared to the control enhancer (_***_ p≤0.0001, n≥40 clusters, Students t-test, +/- SEM). (G) Quantification of GFP positive dAp cells in different mutants shows that all mutants significantly reduce the GFP positive dAp cell numbers (_***_ p≤0.0001, _**_ p = 0.0006, n = 10 embryos, Students t-test, +/- SEM). (H-K) Eya and GFP staining on the *dimm-CRM-GFP* with mutated potential binding sites for Hox (*Antp*, *ap*), Col and Exd (indicated by Δ) at stage AFT. (L) Quantification of GFP levels in the Ap cluster cells, shows that single factor binding site mutation for Hox and Exd leads to a significant decrease of the *dimm-CRM* activity, when compared to control levels. Mutation of potential Col binding sites result in a slight yet significant increase in *dimm-CRM* activity (_***_ p≤0.0001, _*_ p = 0.034, n≥30 cells, Students t-test, +/- SEM). (M) Quantification of GFP levels in the dAp cells, shows that single factor binding site mutation of potential Col and Exd binding sites, leads to a significant decrease of the *dimm-CRM* activity in dAp cells, when compared to control levels (_***_ p≤0.0001, n≥ 30 cells, Students t-test, +/- SEM). (N) Quantification of GFP positive dAp cell numbers shows that mutation of potential Hox binding sites in the *dimm-CRM*, leads to significant reduction of GFP positive dAp cells when compared to control numbers (_***_ p≤0.0001, n = 10 embryos, Students t-test, +/- SEM). (O) Schematic representation of the *dimm* locus together with the enhancer-reporter construct. Colored dots represent the potential TFBS in the *dimm-CRM* fragment together with the conversion pattern used to mutate the indicated TBFS motifs.
Genotypes: (A) *dimm-CRM-GFP/lbe(K)-lacZ*. (B) *Antp*^12^, *lbe(K)-lacZ/dimm-CRM-GFP*, *Antp*^25^. (C) *ap*^*p44*^*/ap*^*p44*^*;dimm-CRM-GFP/lbe(K)-lacZ*. (D) *col*^*1*^*/col*^*3*^*;dimm-CRM-GFP/lbe(K)-lacZ*. (E) *eya*^*Df*^*/eya*^*Cli*^*; dimm-CRM-GFP/lbe(K)-lacZ*. (H) *dimm-CRM-GFP*. (I) *dimm-ΔHox-CRM*. (J) *dimm-ΔCol-CRM*. (K) *dimm-ΔExd-CRM*.
(EPS)

S6 Fig Related to Fig 8 Summary of results. Summary of the experiments showing: The effect of CRM mutations on endogenous protein or Nplp1 expression; CRMs in mutant background; mutation of potential TFBS; and the effect on the *CRM-lacZ/GFP* expression compared to their respective controls. Blue labels = no significant differences, p≥0.05. Yellow labels = reduced gene expression or CRM activity, 0.05≥p≥0.001. Red labels = strongly reduced gene expression or CRM activity, 0.0006≥p≥0.0001. Green labels = increase of CRM activity, p≤0.034–0.0001. Grey labels = not determined.
(EPS)

S1 Data Transgeneic enhancer flies. Outline of the generation of the different enhancer-reporter transgenes.
(PDF)

S2 Data CRISPR/Cas9 enhancer deletions. Outline of the gRNAs, and the sequences of the deleted genomic regions.
(PDF)

S3 Data Wild type and mutated CRMs. DNA sequences of wild type and mutated CRMs.
(PDF)

S4 Data Bioinformatics of Transcription Factor Binding Sites (TFBS). Description of the identification of TFBSin the CRMS.
(PDF)

S1 Supplemental References (PDF)
