# Supplementary material for: Neuronal cell fate specification by the molecular convergence of different spatio-temporal cues on a common initiator terminal selector gene
Source: PLoS Genet. 2017 Apr 17;13(4):e1006729. doi: 10.1371/journal.pgen.1006729 (PMC5411104; doi:10.1371/journal.pgen.1006729)
Supplement: S1 Data — Outline of the generation of the different enhancer-reporter transgenes. (PDF) [file pgen.1006729.s007.pdf]

SUPPLEMENTAL DATA 1

Transgenic enhancer flies

col-Tv-CRM; length: 6389 bp

All col enhancer versions (wt and mutant) were synthesized and flanked with 5´NotI and 3`XbaI sites and cloned into the placZ.attB vector. All enhancer reporter construct were landed in the attP40 landing site on chromosome 2 (estimated cytolocation 25C7).

col-dAp-CRM length 3475 bp:

The col-dAp_CRM was based on the Janelia Farm Gal4 line (49248) and the enhancer fragment was generated via PCR, cloned into the TOPO vector for further sub-cloning via BamHI and NotI into the pEGFP.attB target vector. Mutated versions of the enhancer were synthesized at GenScript and delivered in a pUC57 vector. Cloning of the mutated versions was done via BamHI and NotI into the pEGFP.attB vector. Wildtype constructs were landed both on the second and third chromosome at cytolocation 28E7 (chr2) and cytolocation 68A4 (chr3). All mutated enhancer versions were landed on cytolocation 68A4.
Primer 1: CATTTTCGTGCGTCTCATCGATTCG
Primer 2: GGGACACGGGTTAACAATGTAATGC

apS2-CRM; length: 1968 bp

The apS2 enhancer fragment was generated via PCR, cloned into the TOPO vector and subcloned into the placZ.attB vector via EcoRI. Injection was done at GenetiVision and constructs were landed on second and third chromosome, in the landing sites VK31 and VK37.
Primer 1: TATCGCCGTAATCACAACAAAGT
Primer 2: CCCGCTGCCAAAACAACCCTAAG

apSJ2-CRM, length: 1262 bp

The apSJ2 WT construct was generated via PCR, cloned into the TOPO vector and subcloned into the placZ.attB vector via 3`XbaI and 5`BamHI/BglI. Mutant versions were synthesized at GeneScript based on the WT construct, flanked with 3`XbaI and 5`BamHI restriction sites for site directed integration into the reporter target vector. Injection was done at BestGene and landed on chromosome 2 cytosite 28E7.
Primer 1: ATGTGTTTTGGTTCCAGGTCCG
Primer 2: CCCACTCATAAGCCTAGAGACA

eya-CRM; length: 1307 bp

The eya enhancer fragment was created by PCR, 5`XhoI and 3`XbaI restriction sites were introduced via the primers. Fragments were cloned into the TOPO vector and subsequently cloned into the pEGFP.attB target vector. Mutated version of the enhancer fragment were synthesized at Genescript, delivered in a pUC57 vector and cloned into the pEGFP.attB vector via XhoI and XbaI. Injection was done at BestGene. Wildtype constructs were landed both on the second and third chromosome at cytolocation 28E7 (chr2) and cytolocation 68A4 (chr3). All mutated enhancer versions were landed on cytolocation 68A4.
Primer1: XhoI; **CTCGA**GGATCACAGAATCCAGCT
Primer2: XbaI; **TCTAGA**CTCGCCCAATCTCAACTC

dimm-CRM; length 1224 bp

The Dimm enhancer was generated via PCR, cloned into the TOPO vector and subsequently cloned into the pEGFP.attB target vector via 5`BamHI and 3`NotI. Mutant versions of the

enhancer were synthesized at GenScript, delivered in a pUC57 vector and cloned via BamHi and NotI into the pEGFP.attB target vector. Flies were injected at BestGene and wild type constructs were landed on both the second and third chromosome at cytolocation 28E7 (chr2) and cytolocation 68A4 (chr3). All mutated enhancer versions were landed on cytolocation 68A4.
Primer1: GTGCCAGAGCAACGCGAATTTAAG
Primer1: CTAGTGTCTTTGATTTGATGCCTTCC

Nplp1-CRM; length 1586 bp
The Nplp1 enhancer was generated via PCR, cloned into the TOPO vector and subsequently cloned into the pEGFP.attB target vector. Since the Nplp1-CRM fragment contains a promoter for Nplp1 (first exon), the fragment was inserted into the EGFP vector in reversed orientation. Cloning was done via 5`BamHI and 3`NotI. Mutant versions of the enhancer were synthesized at GenScript delivered in a pUC57 vector and subsequently cloned into the pEGFP.attB vector. Flies were injected at BestGene and wild type constructs were landed on both the second and third chromosome at cytolocation 28E7 (chr2) and cytolocation 68A4 (chr3). All mutated enhancer versions were landed on cytolocation 68A4.
Primer1: CTGCCCTTTTGCAGCACCCACTTCC
Primer2: GCATGGATTGGTTGAAGCAAAAGCC
