# Supplementary material for: Neuronal cell fate specification by the molecular convergence of different spatio-temporal cues on a common initiator terminal selector gene
Source: PLoS Genet. 2017 Apr 17;13(4):e1006729. doi: 10.1371/journal.pgen.1006729 (PMC5411104; doi:10.1371/journal.pgen.1006729)
Supplement: S2 Data — Outline of the gRNAs, and the sequences of the deleted genomic regions. (PDF) [file pgen.1006729.s008.pdf]

SUPPLEMENTAL DATA 2

CRISPR/Cas9 enhancer deletions

CRISPR/Cas9 protospacer primer design

Protospacer and PCR primer sequences used to build tandem gRNA constructs. DNA sequencing files of the CRM mutants used in this study.
Light blue = sequence complementary to the fly genome

primer 1: col_dap-CRM
5`tatataggaaagatatccgggtgaacttcggaattggtttttcgtagcc gttttagagctagaaatagcaag 3`
primer 2: col_dap-CRM
5`attttaacttgctatttctagctctaaaaccatagggcagtgcatttggcgacgttaaattgaaaataggtc3`

primer 1: col_tv-CRM
5`tatataggaaagatatccgggtgaacttcggattatttttcctagtagg gttttagagctagaaatagcaag 3`
primer 2: col_tv-CRM
5`attttaacttgctatttctagctctaaaacatggtgacttaggggattacgacgttaaattgaaaataggtc3`

primer 1: apS-CRM
5`tatataggaaagatatccgggtgaacttcggtagacagccggacggagagttttagagctagaaatagcaag 3`
primer 2: apS-CRM
5`attttaacttgctatttctagctctaaaacttttacattaatacttgtccgacgttaaattgaaaataggtc3`

primer 1: eya-CRM
5`tatataggaaagatatccgggtgaacttcgacgacgccgcccgacacgggttttagagctagaaatagcaag3`
primer 2: eya-CRM
5`attttaacttgctatttctagctctaaaac aaacgaagttttcattgaacgacgttaaattgaaaataggtc 3´

primer 1: dimm-CRM
5`tatataggaaagatatccgggtgaacttcgttggtggtaaaattgagtcgttttagagctagaaatagcaag 3`
primer 2: dimm-CRM
5`attttaacttgctatttctagctctaaaacaacctaaatctttggaaggcgacgttaaattgaaaataggtc3´

primer 1: Nplp1-CRM
5`tatataggaaagatatccgggtgaacttcgtatataaagtcgagggtccgttttagagctagaaatagcaag 3`
primer 2: Nplp1-CRM
5`attttaacttgctatttctagctctaaaacacagtaccgctggctccaac gacgttaaattgaaaataggtc 3´

Sequenced CRM deletions

col-dAp-CRM mutant 2.5

Green: primers to amplify the enhancer region and the deleted fragment.
Light blue: gRNA location
Red: deleted sequence

cattttcgtgcgtctcatcgattcggtgacca
agcaggtgagttcaaaaatatttcaattagttatggatgaactcaacataaattataagaaatggtgctataact
agcaacggaacatacatgtttagaaaattagaaaatcataaataatatacattaataacaactcaagttttaatc

ctttacacaacttgaaagaagtaatgcccaaacatctaatttgaataggttgagtaatttcctgcacagaaaact
cgaaacaacttcaaaaccacttaaatatatttcgaaaattagcaattcatcttgcgattgccacttacataaatt
aagaacttcctgattgccacgctgcttcactgcaaattaaggccaaagttaaccaggctacgaaaaaccaattcc
tttcacttggctgcttccgtgcattttccctctttcccggacacgatgtttgttttgctgattcgagcataagtt
gcattcgaaattgattgtcatggctaattaagtgtgtcctggtgtgtcctttggccaataaattgagtcgtcaaa
cgagcagctcgttcggccaggacttttccgacttttccgcgtgaaaaaaaaggggtgtgaccggggctaacgaga
gaagcgagcaaatcgagagaatcgcacggggatcgaacattgttaatgcgctttgttgtgggtgcgtgcaggtcc
tggggcattatactggtccttatagtcctgctgcaccatatgtccgcttttgtcggcctaatgccctgcgagtcc
tttttcttgcctctccttttcggccttttcaagggcaattaatttctcgagtgacaaatttttatagacattgg
acgactcctttcgccgtctcttccccattaacccccccccctctctctctcactccaatcggtggcactact
ttctcgatttctcttcgtttttgtgggcgttccttgcccatttagcccgttcgcatcagaaaacgagagcgcaat
aaatttgtccacaaattatcgacagcatgcgacatctcgccccctttttctttactttatggtgacaaatccattt
gtgcgaatgcaatttaatcgcaaaattatttgagcagccaccaattgaatttgctaaaaatatcacacacgaaaa
aaaaatggccgtaaaagaaaaggtctctgtgaagttttgtttaattttttttttaggctttgccgataagaagat
tatttatgggcattgttttaggcaattaaagcttatcgacaaattgaatagcgccatagagagttactttaatat
agcaaatgagtaattacaagcagtttaatgggtcttcaaggaaacatacgaattcccgtgctaaatgcgacagat
tcccaaatggatactcattgtacaactcactcaagtttcaatttcacaataatcgaaatacgaatcgggaagtaa
aacatacatataagtatggatgttatattttctaagaataactattacatcccggttagttccttcctccgattt
ccctaaattgcccattttcgcttgacttgtggcgtatttttgatagacctacccctaaacctgggaaaatcacac
agctctgcgagttttgtgcccatttgaaaagtgtcgcaagctgggaaaagaacaatcatttgatcggattaggtg
ggttcggctgcctctttgtagaccctttgttttcgactggatgtttttgaattttacatttcgcttgtcgcgtgt
cgcttttctttatcggattgcgtaacaggtaaggcggggataagcgagtgttatctttagcaagtgggattgcaa
ctcgaaactcgaaactgggaactgtggcgggccgaatgcgattaaggcggaattgtctcaatcgcaaatggcagg
aaggcaggacaattgtcaactgccatccacgtggcaggaccaaagaaaaaaaaaatgaaaatccaaaaaacaagc
agcgacgaggcacaacccttgcaacccctttcctttcgggcaaatgcagcagcaaatcatggtcataatcataat
tacattcgatcagataacggccaaaaacagttcgaacgggaggcgtcgcaattgcaatttgagctaacgatggca
ccacggaaaatcgcctcatttcggcgatcatatttcattttcatcagcggatggtggatagatagactggtgcta
taagggtccggcataaaacaatagccaaatccttttgcccggtttttgtgttttgccaaaattgcgcttatcgcg
attaaaacgcacgcacaaaagcgcatcgaaattctaaaccaaaaagagatggatagcgttcgaggggcagagagg
agagacggcagataactggcgattaatgcactgagataactgagttatagtggttgaaaggagcccaaaaatcca
ggcgaactactactaatgacaaagatccgtcaaccggttacccgatttatccaaccgctaacggttaaaacgaaa
atcggaaagcagcatcattaaataccctatagccattgcactcgcacacacacacacacacacactcgcacacat
gctgttgtctgcgcgtgctaattaagcgataaaattaatttgtatgtcaacacttaaaacgccaaaagagagaga
atcattttcgattattaacaaattgtacgagcacagctcgcttaatggcaaatggttattgttgttgtcgctggc
gccgccggccactcaataaattcaacagattgcgcgaaatctccacatttgcatgtgcgccgccctccgtccgtc
ccgctcgcaccagcccgatttatggccctccccacgctggctctccctttcgcacgcaatgcgattaaaaatgct
tttatttcacggcaagtgcatttctgcggtcgaaagagatggcgtaaagagcgagagagtgagtgagaaaacagg
cggaaaaatgatatcgtcctcattatgcgaaatgtgtaatgcatgcggcatgctcccctttgaaattcttttggc
tcccttggggggaacttcggttttataaaggtgtgccaaatgcactgccctatgtgggtgaaagcaatcttaactt
taaataaatgagcaacgtaggttacatactttcgatgcttacaaattgaaaatatttaaatcacaataatattta
cattttcaaaaaaaaaaaaaaaaacatagattggtttattgaaaggtcacaatgctctttagaattttttatcata
attagaaacaatcaataatttataactgccaaaaaaaggtatacacagctctgtatttcataaagtgcatatttc
aatacgcttgcttatgtaattttttcaacaagctggcattacattgttaacccgtgtccc

col-Tv-CRM mutant stock 7.2
Green: primers to amplify the enhancer region and the deleted fragment.
Light blue: gRNA location
Red: deleted sequence

cacgcactacttaagccaatctaccaccttaattccgtttttttttcttttgaacacatgaaaaa

attcaattccttagatttatattaatctaattgaaatccacaaatttgttgccatttgttgtgcagaagtatttt
tgaatttcgcacacgttcgttggcaaaaatatgtacaaatattgaatacgaaaaagtcaaggaaaaattccattc
tttttctcgcaaaaatggtgacagagcaaaatgctgaccgccgcctactaggaaaaataatccccagccgatttg
aacgatttattatttggcccagccaactcggagcctgtcccggaatacccggaaccattaatcatccgagtccag
atccaagtccgagtccgagtggtggacaggcggcattgcaattggtggacattttggagtggaagaaacttttgc
ggtacgtggcaacgtgttgaaattacaaaatgtgttaaaaaatgaaagagcagggcgggtaaaaagcaaaataat
atatttcattcacacacagacttttgtctctgctccgccgtccatataaaaaacataaatttcctttgagaatag
aacttctgggatggagagggggggggggggggtgcaaggtgattatgtttgacagccgacttgggacacgaagcac
cgccccctttttcatcgccaaacaggacatgggtattagatcctggccaaagtgccaaaactggcctcactttcc
gtctgatttacggccaactattttgtttaaatttttaccacttccctgctatcgacactcaacttaaaacatata
aaaccaactatttaaagagcaaataaagttcaatttgattttctttggccttcgttaataattttcctgcatctc
tctggggatcagcgaaaaatatgaattattgaaacagctgaaggaaataatataaccaaatcggaaggccaaatc
tgtttaatgtttcagatatgttggtgtgggttgaccaaatgacaatatgctttttagcttcaacctccctcaagc
gaaattgattttcatggtttttctgtttttttcgcctaaaaatacacaaaagagtcgaaaaaaatttgactgtgatt
gtcacttgaatgtgtgggttgggaaaaccactccaaatccacttcaccaaatatgtcagtatggaatcacgaacc
tcgcttgatttctccagtgatttttcgcaatcagcctgatgaggatgtgccgagaacgttgctcatggaggcagt
gctatgattagtatcctttcagccaggtcaaaagtgacgctgccgcatgccaagtggcctcggccaaatgaaact
ttaatgcgaaacaaggacaaaaggctgaaagaaggctgcctgatgaactacgtgatggtcagacaaattgaagtt
acactggaaaataattagctaatttcctagtttgcttaggttaagggattaagtaagaatgtgtttaaaggactc
ttttataggtaacttgttactctaaagaaaacttatccacagaatagatgtgctattttatcgcagtgtatgtgg
atagtcaacgacaaagtgaaatgcattgaggttggtggcgaacaaacgcgacctgaatcgcgaattggaatagcc
agcctactccgccccctcaggatatatagctacttcgactagcatatgcgccagatagtatagctggcgtatcaa
tttcgataatgtacttggaccgcgagtatctttattatcctgactgctgtgcaattatttccgactgctttactg
tcgtaccgcttttccgagtcctgggaaacgcaaacaaaggatatttcgcgcacacgcctcggacatgattgcatc
cgggcaggaagcattgcgtcgtgtcctttttgcatccttacatcgtcgcttccttcctgccgatcagaaccccctg
gctgcttttcatccgcaattttcacgcccactttgtttgatgatgacaaatctcgttgcgtgttatcagattggc
caactttccgttgctattccccctcgtattgcataattatgtttatctttggaagctgcgctcctgccacgtgtc
cttgcgttttggttagcttcctgcgattttgctcggaacgagtggcacgtgctaatcggcggtcgtctgcaccgc
ccatgcaaccagccaccccccctggtaagtgcaaccccgcctaacatgtccgataattgaaatcgctaagctgaa
caacggaatcgaaatggcttcattaatcaggtcacttcgatggcagcgcataatgttcttcaaaaatttgaattg
ggggcggttgaaaagaggaaattggtctatatagcacttagcgcgattgaatttgctcattttgtgttacctaat
tggggaaaagatgtttcggttattaatcatgagtgatacttactattgtattacaaaaatggtgcatatatcaga
agatatatattattaatagatcttaaggagcatttgtatccattcaccaaaatatggtaaaaataaagcttaaat
taaatttgctcatttacctttaaggacatcctttgcttaatgagtgcactcatgcccacacccactcgcaaccga
atgagtgtcgccctgcggacgcataaattataaataaaagcctcacacacgtggagaaagagaggtgtatataac
cttagagagagagagagagggagagctacagtctgaggccaagaagtgtgctaaatatttaagtacttaaaacca
taaacctgagtatttataaattggctttaacaagctcgtatgtgacggtggttgggtgtgtgtctgtgggctgac
cctttttttcgggcacaaggatctgctcgactcgattccattcattagaaatgctcactaatgtgcccgacttaat
aataatcaaatttatatgcatggttatgtcctttagagcaggaccacaaaactgaagtttcgagtggattgcgaa
tgggattggctgttaagtttacggagaaacctttgagaatgagacgaagataatatgtcaatatccttgttttcc
aaaatgtctgaccaaaagtgcagatgtccagtatcgaaacacgcccaagaccggtaccgttattctgctaccagt
tttcttgtttgtttttgtttgttagccagcttgtttgtggccgacactgagaccaaaaaggccaaaaaggtcgcc
ccctcgggatttccttagtcctgctgccagcttcttccttccacattctttttgggggtccgatgagatttgaaa
atttcacaaaaattaattaggaatgctggcatccatgttgtgataggctagttcttttctagcctagcaacattt
ttgatgtgactaaagagaatcatctttatggccacacccggaatactcattcttcaaacaacaagcgacaataaa
aacaaattacaattgcaacaatggcagcggcagcaaaacatttgagacattttccccacacacacacacagacac
acacatacttcagttaaatgaacatttgcaggacctagatatttatttattacaggggttgcactgcaggagcat
taacccccttactgcccacggcagtctcgagcaacggaggactgaaaaaattgaaataaattaccaaaactaaagg
caacaactgttcgcataatgaagtcaaattatatttgtgcctaagtgcactgagagaaatttaatataacctatg
atttacacgatcgattttcgatctttgcaacatttttttgagtgaaactaggaaaccctttacaagcgagatcttg

cagttctttagtttctaagcattattctagtattttaaaatatattttcctttgttctggtcttaatttattata
actaatgaatattttccgtctgtgtattcggaaaagggggagttggaacatataaagatggggtgcagaaaagat
gatcagggaaggtgtgagtggttcaaactagctgggcgtttggatagcgaagggtgcgtcctttgctggtccttc
gaattatgcaaatcgtctgcacttcagatactactacccctacaaaaaaaaaaaacgaagagaaaaccccctaca
aacaaacgtattagtgtcatcgagcatttgccaagccatttcattgacacgtagaacaccttttttcgtccacttc
gtatgatgttatcatttaattcttggggaattaacacggacattgtcaaccgtaaatgagctaattaatctagta
aatgtgtcgcagctcattccacaatttctctactacttgagagcgaggagtgggttcgcgtttttgtggttctcc
agaggatccccaatgctgacacgcttctccaggtggccgaggagtgggaaatggaaaatgggcagggtttgcccc
tcggggagctgcactaaaaccccaaatgagatgcatgaattcctcttcgccgacaagcagctcataaaagttaac
atcgaagtatttacagcatattttggggaaacttcaacttggaatttgattaaatgcaaactacttgaaaacata
caacttcaaatcaagtatgtacaattttaaatcaagtagatctctttgaatttattgatttgcttaaccaactta
agtttccttaacttctcttatttttgtgtattttggctaactatttttggccacaaatcgatctttggtcgcgcc
aaagttaatgaaccaaccgcacttttccaatctcgcatttgatgcgttggattccacatatttggccagactgaa
aaataatttcatgtttatggccacgttgttttcaccggccacaaaaatgcttatcgaacgccggagatcgccaag
cgagagacttgtaaatatttatgatttttttgcgatttttattcgactgataaacgagtttttcccatcgcacagt
tgcgatgtgcgaaaatgttttgtgggaagaaattgtgcacctagcccatacgattcgattcaccttctgtttgtt
ggaataataaatctgtagaaaggttgttgtatttttagaagactccatcccccttttgttattcttatgtaaacga
aaccttttttcctacacacatagctgcatagtaagttaccctcatgagaacgtttaggccgtgaatattgggtaaa
tatttaaactggttgcgattgttttggcaaaatcgttgcagttggtcaaacacgtgtttgagataatttaattaa
atttacttggctttttttaacccgtgccacgtgccactcgagtccagtttgctgccctcgaaatggagtggcaaac
acttgagcacttgctttaaaaatgcggcaagtgcatttgggttgggtgggtagtttagcttggttttggctggct
ggtgaaaattcctaggaatcgaaaactttggtggtcttcggacggcagacaatgcaacaatgtcatacgtacttg
gatgaatcgctggcatctgtcgcagtccgtggagaatagaccgaaattcccccgaactcacgagcatgatgtggc
gatgatgacttgtggaaatcaagcaatgatttccactccatctgtaactaaatatgatttattttcacggtgtgt
gttagccgctctcgcccctctcattagtcggggcaccggggtaatttgcataaggtcctggccccaggaaccaaa
ggacacactcgagagcgagctggaaactaattaaaatgttcgcgcctcaaatttccttcgctcgcccttatttat
gaccctgtgaatgtcttgcatatggacggagttgagatccttctcgttttcttcctttttttgtttacgagtgac
aaatgggacatttacatgcgagtcagtttgcatattggaattattcatggtttctttaaagatggcgcaggatgt
gatgtcgcgggaaaaggatgagggtttcctcaaggaagtcggaaatagaagtggtttcttttcatgctaccaata
tgggggcacataaaacttgcatttgatgagctaaatgtaaacatacactatttaaatacattttttatgataagt
gaacttaaagtcacactaacttttgaaaatttgattgacttctacaatgtgtgttgtttaatttcttattttata
tttttttaaatatcgaaaatctacaaatccgcttatgtttaaaagtcaagccgctggctaattgacaaaatgtgt
aattgtggcgatgagagtccttccgattgtcactctcccaaccctccgtaatccccctaagtcaccatagggggttg
ggtaaaaatcaaatgcggagaatgtaccaacaaattatttagcaattggctagtgcgctagtccggcgccatgta
aatccaattgtaaacaatttgaatcaaattccgggggcatcgccaccgaaagggggtggcatgggttaaagggtca
tggtgttccatgacagctgtcg

apS-CRM mutant stock 3.1
Green: primers to amplify the enhancer region and the deleted fragment.
Light blue: gRNA location
Red: deleted sequence

caccctaaagcggtcctagtataccggataccctagcataccggattttactgtataaaaggaagttgc
gatcgaagctatatgatattttatgggtatgctgcaaccacatgctgatacaaataatcaaagaatatgacatacttata
atgaattgtagacaaaatttgcagttcgcagcttatctcaggtcgtatttcgatgtgcgaattcacacctaattggtttt
taatccataaatccattgttttgggatgactgacgacgttttgtttgacgtacaaattaaaatgagcgccatccccgaga
tcagagggtaaaactcacaatctaaacatcaagacagaaggaaatcacaatcttcatgcttagtctccactttctagctt
ttatagttcctgagattaaggcggccatgtggacaggcgggtagacagccggacggagatggctaagtcgacgtgttcct
ccttccaaccgttacatacttttcaacgaatatacaatagtaaactgatataaatatgttccaacttaaggtctaagtgt
cagcggactcatacaaaccctttcagacgaatttttttcttctttcagagtcaagtcaaaaaaaatccaaacatgggta

ttgaacagaacaagcaggaacgtaaatgccattaaataaatttgtgaattagaactatggtctgtccagtttacttcgct
accgaatttggttttcttttcataatgtcgtcccttgggttctaatttgtgacaatcatcctatgttggcagagaagaat
atgatgcgactgtgagcattgcgcagattggctatttgttttaatggaatcagaaacgagtacgcggcaccggaatcaca
ggagaactgcttttatctgtccccaaggcttggcaatatttcttgccacactgtcatcaaaattattatatcggcgcgtg
tcggggtgatataaaggaatcgacttgtaggcctaacttttacatgtctcatgcggttatactacgcttaactgtgttgc
gcaagcgtctccacttttatgtgcaagggtcaaaagttgttttgtttacaacatatcattgcactaatgaagtggtcaat
acttgatttggttcattaagggaccggggatgtgttttggttccaggtccgttgggaacccagcttcaaatgaagttatc
acagcgattatattccttaaaacgttcagaaacctccgcccgtgcaatttgaacagctttctggggtgggagtttaattt
tgagggtttgtgtataaaaggccaaacaaccctaattgaaattatgcggtgtggctctgcagcgactgctcgtttaggct
gtggtattccgaagggagtactaaaggatttcatcgggcgggttgcgaaccttttgcggcggaactgattagcagacaat
atgtcaaagcttgccactgggagggtgaactctgtagcttcacccttcaagcggcgttgcgactactccagggacagcta
ctcgaggctcggctgataagggaaaaagtcatgacaaataaacggatacatgtgtcggcaatagttttgcttattgccgc
gataataatcgacgtgaacaacaatggtctttgtcctggtcatgctcctgctgcctgggtgcccacataaagaacaatcg
cgagcgcgcgcccaacgagaattacaggatcgtttatggcaaccgcctggtagcagtctcctggtagcacccgctgccaa
aacaaccctaaggcgattatctcaagtgctcgcccttccaggactatgtttgcctccgtgcaccctgctgtagatgctta
tcgattacacggtgagcgatgaaatcattactccgaattaaataaacgtcatactcgcttttcgtcaacttgtatttgat
gtctcaatgagcgagcgtgccattggcaagggaacccatttgcatcgcagatttagaccctggcactgtgatagtgtgta
aacacgtcgcaattagttgacctaaacgaatgccccggcgtaccgtcgtcttccggtcagagtcccggaatgacatcaag
ctccgattagtcactttctagttggttgggagccaagcagctgcctgccatccgcaactctcgctcttattgtttgtccc
catatctgtatttgtcgcacaactttgttgtgattacggcgataaagcggctcaagtaaatcacaataatacgacggtct
agtctggccgactcggttcgaaagaaagtgtgaatagagccgggctcaacaatcaacaacaatggattcgccggaccgga
ccagcaatgacaaatgtctctaggcttatgagtgggcatgtgcataaaagtcataagcaatagaaataataattgtgttc
acgtcatgggacacgtcaacaaaaactgccgagaagcaattgcgcgacactttcttgggtgtaaattaagatactttata
gctggggtggtgccgtggtcgcttccgctggctactggttttgaacccagggccacgaaaccagttccagctcaggccct
tggtgaagtccaagctggtggccagcttgggtttttgtgtatcactcgattgtatttgctctttgcatgcgtcagcgat
atatatatattgaaccgctagcatattcattgattttcgaacagtaattctgcaatatcattgcctgttcaacttcgtgg
gactagcacaacccatctcttgcctccccgcaccgtcccgcgaagaaatgccgtcacgtacgcgagccccatcgttcaac
aaataaagaatgaaccggatcctcggaaaggcgtggcatcgtaatgatactgatgcgttagtaaaattgcggtcaaatta
taggaggccagctctaaaatcgagcgaccttttgaaggtaggcaggaaatgcgaaattcggtagcctgtttattaaatac
gaaaatatatgaatccattgcgggctggcaggacctaagttgtggggcatttgtagccgagatcagaacggtcagctacc
tgtggatgggtcccgaaatcacacgaaatagtctaagctataaattagatttttatctcgttgccgcaggaataaaagga
ggcaagcctcctactgacgctcgatctctggagagctgtcgcatattggggcgatccaattaagtggtaatttttatcat
tttagcgtcgagggccgggttagttctgttgggtcgcttttactccgaaatatttgctgacaaacggaagcgatatgttg
aaattattcagcgtaaggattccatgaaccttcggcagagcctgggagtgagattcagagctggaatttgaatcgattcg
agggggctacacccgcttcgatgaaacgatttttttgtcgggccttgcttgattgactttactcaagattgacttcatgaa
ggcaatttagaattgggctgataatcagtccggtacataagtcgctttatttgccttgattatatgcacagtgtgcattg
cacactacggaagctgcactatgactgaggggggtcataaagcgtaggtgagagggtcttgttcgcggggtaacttgattt
gtttgtctatttgttcgcctccgactctttgcttcgactgcctttacagggaggacaagtattaatgtaaaatggtaac
tggtcatctgaggcgtcatttgggtctggctcggggt

eya-CRM mutant stock 3.5
Green: primers to amplify the enhancer region and the deleted fragment.
Light blue: gRNA location
Red: deleted sequence

ctcgaggatcacagaatccagcttgctgggtatgctgtagtcgacgcccttcgcacgttccgccaatttggctgcgttgtttcttgggttaa
ccgttgccaggcctatgaaatggtgcttgcaaccactcgccccgtccggaaaacaacaacattgaggcatcgctgacaaattgaatttgt
aaacttgtccgcgggcaacaacaaatcattggagacccccagggatgctgtttcggttgctgatccgtccgttgccataaccatccggat
tccggagtcagcaggaggaggaggaggaggaggacgacgccgcccgacacggaggtggatgcgaatgccatgggacgctggta
atccaatacccatgccctatccatgttacgctcctctggacggactttctaactcggtctcagttcgccggctcggttctgtcctcccaacc

agccgcccatcactcccccacaaaaacagtggcgaaagtgatgtgaaattatagacttgctttcgggcggttgggggcgtgtcaacgc
agttggaaaaaaaaacacacacacacgcttacgcacaattattaacacttattaatgttgaggtttccatatatatggcaccccagggctta
aattaaagtcatcccgagtgccaaacagctgcctcaagtgccgcacttgagccgatctggatcccggaacaggggggttgcaacccgg
gggttgagtggggttgggcgttggctttgtcgctgtttaagtagtcattaagatcgagtgtggataagatttttcacagctggctgtcctgc
cgttggtaccagccagtacattcgccaacccctcgccaaacgaaaaaaaaaaacaagaacaagaaccccgccttgcaattagccaac
aacctttgactccggcccaactcccttcatggtgagccggaatcctgactgtccggaataagctattgacagctattgtcttctaattaaac
aacaatgttgacctattctcaagatgtcacggatcgggttcaatgaaaacttcgttttgggttaggaggaggaggaggacgccgttaaaa
aaaaatcattgcaaattatttacactactttttcagaaaatcggttgcattgtttgttaattaaaaacttttgattgctaggcgaagaacaaccct
taaagaagttcgccttgctaaccctttgagtttatctgctgaagaagttgcttgttaaccctttgagttgcgcttcaacccttttatccttgtaat
aattaagttgaaggaaagacagtattgtaaattataaagttgagttgagattgggcgagtctaga

dimm-CRM mutant stock 2.5
Green: primers to amplify the enhancer region and the deleted fragment.
Light blue: gRNA location
Red: deleted sequence

gtgccagagcaacgcgaatttaagcattggagttgagttggtggtaaaattg
agtctggagcacaaaaacccttttttttgggatgatttatgctgctgccagtgctgctctaaaaagcgccgaccaactat
ctttgatattggtaacaatttttggccttaaaatatttgtccgtaaacaaaattcattagcagagaagtcagcaattaaa
catatgtattataaatggaatgatttcttgaattatggttatccgaaatataatttttgttttttaatttattgacccag
gactttttctgaaaaggaaaataaataaataaattttgttttggcgcgaatttcttttctgcatttatttccaattta
gaacacatttcttttcacactatttcgaaatcgaatccacatcgataatgtaaaataacaccgaccgtcgccgcaagtcc
ccatagaagtcgagaggctagtagaggaagaagtcaacgtcatcgttaaatttaattaggcatcaaatgacccaacagcc
gagctaaatcaaaaaccaatggcctgaaagtgacccgagtgcaggcgaaattcaaccgcctggctcaaagggcggtcgca
acactttaaaacacaattaaaagtcaaaacgtcattctaaacgaaagccagtggacaaccctccaaggaccgaagagccg
ccaatgaggcgccgtagagaaagtgctgatgtgggatggagacatggcgctcactttacataacatttaacaaaattata
atgagacatagaatggatgtatatcggtcggggggtgagtcttctactcccgtgtcgaatgttaatgggattatgtgagg
aatacattttaatatttatttgaagtattatcctagcgagataaccgcattaggtggtgtccattagaatgaattcaatt
accgccaaagctaagtggatcagaaggaaactataagagaaaatactaagagcaatttttttatataattaccgcaaaatg
cacatgtaaccaatgtggcctatctctgcgtggagcaacataattacagagcgagcagatgcgaccgaaatgtaaacagt
gtttcttccgaacctaaatctttggaaggcatcaaatcaaagacactagaataattctagaataattctagtgtctttga
tcaaatcctgttgtggaattttatgtaattttattatatcacgcatcgccttatttaagctttatatttacgaggtcttt
tatgggattccaccatgaacacctagacattctagagatgagatctgtcagttggcccaggtctccgaattgttcggaat
gtcttactatgttttcaaaatatgatttatcatatcatcacttttggagaaaatattacgtataccgtacgtattgttgt
taagcgctcagaattgttcttgatttaatgtactttttttcgaagtctatgtatacctccctgcagtaa

Nplp1-CRM mutant stock 3.4
Green: primers to amplify the enhancer region and the deleted fragment.
Light blue: gRNA location
Red: deleted sequence

ctgccccttttgcagcacccacttccaaaccgatttaaaccgtaacggaaacacggacgcgaagctttgccgtttgccagctgccgtaac
actaacgttagccgcaaagtcgtctgctcgactgctttctcttggccaggaccctcgactttatataccgagcggcaccccggataagc
tcactcgcgggagcgaaaccggagcgccgtcaactgttgctcttctatagcctctctgttgcgtgtgcagtgtcctgtttcgtgggatgct
attgtaggtaatgacgtcatggcggctgcaggacagagctctgttgcttcttggtaagctctgtgtattcaggccaaccagcagcacagt
gcacagctacaaagcttttcgatattaaattggtattcgcaggttacattattgattcattttgaaaatataatagaatgtttgcaggccaaaaa
aaaattaaaatgaatccaagtccaaaataaaaaaattaatagaaaacccttccttagctacgatttttcgggtttcggtataatttgaattact
cgaaaccgttttcttatcatttttctttcaactttgcaagttcaatccactgtgcgctggttgctttgggaccaatctaagagtttttggtttctctt
aattcgaacttgagctgtacgccatatgttggcgctctcagcggggccactgtccgtcgcagaacttaacacttaaataaaaaaccttga
atcaaacatatgcttcaaatattctttattggaatatttaaaatcacacttgaagttaatcagagtaattaaaacacaaataggctttgttgaaat

atttttctaacttttgtgtttgtatttactatgtatgattacatttaatctgattatcggatagcaatgggctggaaatacattatcaatcacatttac
ttttcgattattcaaaataaacatttttattattttcattgattacattcggtttttaacctcgaatcttttcatcgtataaaccaaattaaaagcattg
gaattcaatgaagtacaaaagaactcccgattagaatggcgttgcagtgacctgtctgtaagaattccgaaaatataaaataaccaaaaa
gccggaaagtaatgccaacagatagattgctgcacccctcccgcagaagctaagtttatgcaatttccgaataccatattatgaagcgta
aaatgttaaaaccagatccccggttatacccaaaaccctgagtgcggcaagagcaatgcacaaaatgcaggggcggtcacaatcatat
gcctacgtcagtaaagcaaatcgctgtgggggtttccccgcgtcactcctaaaagaaggatatatggcggatggcagatgaagcggc
ggatgtagcagattgcacgccggtacagacagctggcagggaaacctgtcagcgcactgcaacataggaggttggagccagcggta
ctgtcggctttgcttcaaccaatccatgc
