# Supplementary material for: Neuronal cell fate specification by the molecular convergence of different spatio-temporal cues on a common initiator terminal selector gene
Source: PLoS Genet. 2017 Apr 17;13(4):e1006729. doi: 10.1371/journal.pgen.1006729 (PMC5411104; doi:10.1371/journal.pgen.1006729)
Supplement: S3 Data — DNA sequences of wild type and mutated CRMs. (PDF) [file pgen.1006729.s009.pdf]

SUPPLEMENTAL DATA 3

Wild type and mutated CRMs

DNA sequence files of the wild type and mutated CRMs.

col-dAp-CRM

ggatccactagtaacggccgccagtgtgctggaattcgcccttcattttcgtgcgtctcatcgattcggtgacca
agcaggtgagttcaaaaatatttcaattagttatggatgaactcaacataaattataagaaatggtgctataact
agcaacggaacatacatgtttagaaaattagaaaatcataaatatatacattaataacaactcaagttttaatc
ctttacacaacttgaaagaagtaatgcccaaacatctaatttgaataggttgagtaatttcctgcacagaaaact
cgaaacaacttcaaaaccacttaaatatatttcgaaaattagcaattcatcttgcgattgccacttacataaatt
aagaacttcctgattgccacgctgcttcactgcaaattaaggccaaagttaa**CCA**gg**CTA**c**G**a**AAAA**cc**AATTCC**
TTt**CACT**t**GGCTG**cttccgtgcattttccctctttcccggacacga**TGTTTGTTTTGCTGATTCGAGCATAAGTT**
GCATTCGAAATTGATTGTCAt**GGCTAATTAAGTG**t**GTCCTGG**t**GTGTCCT**t**TGGCC**a**ATAAATTGAG**t**CGTC**a**AA**
CGAGCAgctcgttcggccaggacttttccgacttttccgcgtgaaaaa**AAAGGGGTGT**ga**CCG**ggctaacgaga
gaagcgagca**AATCGAGAGAA**tcgcacggggatcgaaca**T**t**GTTAATGCGCT**t**TGTTGTGGGTGCGTGC**ag**GTCC**
Tgggcattatactggtccttatagtcc**TG**c**TGCACCATATGT**cc**GCTTTTGT**c**G**g**C**ctaatgccctgcgagtcc
tttttcttgcct**CT**c**C**t**TTTT**cgg**CCTTTTCAAGGGCAATTAATTTCTCGAGTGACAAATTTTTATAGAC**attgg
acgactcctttcgccgtctcttccccattaacccccccccctctctctctctcactccaatcggtggcactact
t**TCTC**ga**TTT**ct**CT**tcgt**TTTTGTGGGCGTT**c**C**t**TGCCC**a**TTTAGCCC**g**TTCG**c**ATCAGAAA**a**CGAG**ag**CGCAAT**
AAATTTg**TCCACAAATTATCGACAGC**at**G**c**GACATC**tcgc**CCCC**t**TT**tctttactttatggtgacaaatccattt
gtgcgaatgca**ATTTAATCGCAAAA**ttatttgagcagccaccaattgaatttgctaaaaatatcacacacgaaaa
aaaaatggccgtaaaagaaaaggtctctgtgaagttttgtttaatttttttttttaggctttgccgataagaagat
tatttatgggcattgttttaggcaattaaagcttatcgacaaattgaatagcgccatagagagttactttaatat
agcaaatgagtaattacaagcagtt**TAATGGG**t**CTT**caaggaaacatacgaattcccgtgctaaatgcgacagat
tcccaaatggatactcattgtacaactcactcaagtttcaatttcacaataatcgaaatacgaatcgggaagtaa
aacatacatataagtatggatgttatattttctaagaataactattacatcccggttagttccttcctccgattt
ccc**T**a**AA**t**T**gc**CCA**tt**T**tcgcttgacttgtggcgtatttttgatagac**CTACCCCTAA**acctgggaaaatcacac
agctctgcgagttttgtgcccatttgaaaagtgtcgcaagc**TGGG**aaaagaa**CAATCA**tt**TGATC**g**GATTAGG**t**G**
GGTTcggctgcctctttgtagaccctttgttttcgactggatgtttttgaa**TTTT**ac**ATTT**cgct**TGTCGCGTG**t
cgcttttct**TTATCG**gatt**GCGTAACAGGTAAGGCGGGGATAAGCG**a**GTGTTATC**tttagcaa**GTGGG**attgcaa
ctcgaaactcgaa**ACT**ggg**AACTGT**g**GC**gggcc**GAAT**g**CGATTAA**ggc**GG**a**AT**t**GTCTCA**atcgcaaatggcagg
aag**GCAGGACAATTGT**caactgccatc**CACGTGGC**aggaccaaagaaaaaaaaaatgaaatccaaaaaacaagc
agcgacgaggcacaacccttgca**ACCCCTTT**cct**TT**c**GGGC**a**AATG**cagc**A**gc**AAATCATGG**t**CATAATCATAAT**
TACATTCGATCAGATAACg**G**ccaaa**AAC**a**GTTCGAAC**g**GGAGGCGTCGCAATTGCAATTTGAGCTAACGA**t**G**gc**A**
c**CACGGAA**aa**TCGC**c**TCATTT**cg**GCGATCATATTTCATTTTCATC**agcggatggtggatagatagactggtgcta
taagggtccg**GCAT**a**AAACAA**ta**G**c**CAAATCC**t**TT**tg**CCC**gg**TTTTT**g**TGTTTTGCCAAAATTG**cg**CTTATC**g**CG**
ATTaaa**ACGCA**cgcac**AAAAGCGCA**t**CGAAAT**t**CTAAACC**aa**AAAGAGA**tggatagcgttcgaggggcagagagg
agagacggcagataactggc**GATTAATGCA**ctga**GATAA**ctgagttatagtggttgaaaggagcccaaaaatcca
ggcgaactactactaatgacaaagatccgtcaaccggttacccgatttatccaaccgctaacggttaaaacgaaa
atcggaaagcagcatcattaaataccctatagccattgcactcgcacacacacacacacacacactcgcacacat
gctgt**TGTCTGCG**cg**T**gc**TAATTAAG**c**GATAAAATTAATTTGT**a**TGTCAACACTTAAAA**c**GC**caaaaga**GAGAG**a
ATCATTTTCGATTAt**TAACA**a**ATT**gtacgagcaca**GCTCG**cttaatg**GCAAAT**g**GTTATTGTTGTTG**tcgctggc
gcc**GCCGGCC**a**CTCAATAAATTCAACAGATTGCG**c**GAAATCTCC**a**CATTTGCAT**gtgcgccgccctccgtccgtc
ccgctcgcaccagcccgatttatggccctccccacgctgg**CTCTCCC**tttc**GCACGCAATGCGATTAAAAATGC**t
ttta**TTTCACGGCAAGTGCATTTCTGCGGT**cgaaagagatggcgtaaagagcgagagagtgagtgagaaaacagg
cggaaaaatg**ATATCG**t**CCTCATTATGCGAAATGTG**t**AATGCA**t**GCG**gcatgctc**CCCTTT**g**AAA**t**TCTT**ttggc
tcccttgggggaacttcggttttataaaggtgtgccaaatgcactgccctatgtgggtgaaagcaatcttaactt
taaataaatgagcaacgtaggttacatactttcgatgcttacaaattgaaaatatttaaatcacaataatattta
cattttcaaaaaaaaaaaaaaacatagattggtttattgaaaggtcacaatgctctttagaatttttttatcata
attagaaacaatcaataatttataactgccaaaaaaaggtatacacagctctgtatttcataaagtgcatatttc
aatacgcttgcttatgtaattttttcaacaagctggcattacattgttaacccgtgtccctaagggcgaattctgc
agatatccatcacactggcggccgc

col-dAp D Kr-CRM

ggatccactagtaacggccgccagtgtgctggaattcgcccttcattttcgtgcgtctcatcgattcggtgacca
agcaggtgagttcaaaaatatttcaattagttatggatgaactcaacataaattataagaaatggtgctataact
agcaacggaacatacatgtttagaaaattagaaaatcataaatatatacattaataacaactcaagttttaatc

ctttacacaacttgaaagaagtaatgcccaaacatctaatttgaataggttgagtaatttcctgcacagaaaact
cgaaacaacttcaaaaccacttaaatatatttcgaaaattagcaattcatcttgcgattgccacttacataaatt
aagaacttcctgattgccacgctgcttcactgcaaattaaggccaaagttaaCCAggCTAcGaAAAAccAATTCC
TTtCACTtGGCTGcttccgtgcattttccctctttcccggacacgaTGTTTGTTTTGCTGATTCGAGCATAAGTT
GCATTCGAAATTGATTGTCAtGGCTAATTAAGTGtGTCCTGGtGTGTCCTtTGGCCaATAAATTGAGtCGTCaAA
CGAGCAgctcgttcggccaggacttttccgacttttccgcgtgaaaaaAAAGGGGTGTgaCCGgggctaacgaga
gaagcgagcaAATCGAGAGAAtcgcacggggatcgaacaTtGTTAATGCGCTtTGTTGTGGGTGCGTGCagGTCC
TggggcattatactggtccttatagtccTGcTGCACCATATGTccGCTTTTGTcGgCctaatgccctgcgagtcc
tttttcttgcctCTcCtTTTTcggCCTTTTCAAGGGCAATTAATTTCTCGAGTGACAAATTTTTATAGACattgg
acgactcctttcgccgtctcttccccattaacccccccccctctctctctctcactccaatcggtggcactact
tTCTCgaTTTctCTtcgtTTTTGTGGGCGTTcCtTGCCCaTTTAGCCCgTTCGcATCAGAAAaCGAGagCGCAAT
AAATTTgTCCACAAATTATCGACAGCatGcGACATCtcgcCCCCtTTtctttactttatggtgacaaatccattt
gtgcgaatgcaATTTAATCGCAAAAttatttgagcagccaccaattgaatttgctaaaaatatcacacacgaaaa
aaaaatggccgtaaaagaaaaggtctctgtgaagttttgtttaattttttttttaggctttgccgataagaagat
tatttatgggcattgttttaggcaattaaagcttatcgacaaattgaatagcgccatagagagttacttaatat
agcaaatgagtaattacaagcagttTAATGGGtCTTcaaggaaacatacgaattcccgtgctaaatgcgacagat
tcccaaatggatactcattgtacaactcactcaagtttcaatttcacaataatcgaaatacgaatcgggaagtaa
aacatacatataagtatggatgttatatttctaagaataactattacatcccggttagttccttcctccgattt
cccTaAAtTgcCCAttTtcgcttgacttgtggcgtatttttgatagacCTACCCCTAAacctgggaaaatcacac
agctctgcgagttttgtgcccatttgaaaagtgtcgcaagcTGGGaaaagaaCAATCAttTGATCgGATTAGGtG
GGTTcggctgcctctttgtagaccctttgttttcgactggatgtttttgaaTTTTacATTTcgctTGTCGCGTGt
cgcttttctTTATCGgattGCGTAACAGGTAAGGCGGGGATAAGCGaGTGTTATCtttagcaaGTGGGattgcaa
ctcgaaactcgaaACTgggAACTGTgGCgggccGAATgCGATTAAggcGGaATtGTCTCAatcgcaaatggcagg
aagGCAGGACAATTGTcaactgccatcCACGTGGCaggaccaaagaaaaaaaaaatgaaaatccaaaaaacaagc
agcgacgaggcacaacccttgcaACCCCTTTcctTTcGGGCaAATGcagcAgcAAATCATGGtCATAATCATAAT
TACATTCGATCAGATAACgGccaaaAACaGTTCGAACgGGAGGCGTCGCAATTGCAATTTGAGCTAACGAtGgcA
cCACGGAAaaTCGCcTCATTTcgGCGATCATATTTCATTTTCATCagcggatggtggatagatagactggtgcta
taagggtccgGCATaAAACAAtaGcCAAATCCtTTtgCCCggTTTTTgTGTTTTGCCAAAATTGcgCTTATCgCG
ATTaaaACGCAcgcacAAAAGCGCAtCGAAATtCTAAACCaaAAAGAGAtggatagcgttcgaggggcagagagg
agagacggcagataactggcGATTAATGCActgaGATAActgagttatagtggttgaaaggagcccaaaaatcca
ggcgaactactactaatgacaaagatccgtcaaccggttacccgatttatccaaccgctaacggttaaaacgaaa
atcggaaagcagcatcattaaataccctatagccattgcactcgcacacacacacacacacactcgcacacat
gctgtTGTCTGCGcgTgcTAATTAAGcGATAAAATTAATTTGTaTGTCAACACTTAAAAcGCcaaaagaGAGAGa
ATCATTTTCGATTAtTAACAaATTgtacgagcacaGCTCGcttaatgGCAAATgGTTATTGTTGTTGtcgctggc
gccGCCGGCCaCTCAATAAATTCAACAGATTGCGcGAAATCTCCaCATTTGCATgtgcgccgccctccgtccgtc
ccgctcgcaccagcccgatttatggccctccccacgctggCTCTCCCtttcGCACGCAATGCGATTAAAAATGCt
tttaTTTCACGGCAAGTGCATTTCTGCGGTcgaaagagatggcgtaaagagcgagagagtgagtgagaaaacagg
cggaaaaatgATATCGtCCTCATTATGCGAAATGTGtAATGCAtGCGgcatgctcCCCTTTgAAAtTCTTttggc
tcccttggggaacttcggttttataaaggtgtgccaaatgcactgccctatgtgggtgaaagcaatcttaacttt
taaataaatgagcaacgtaggttacatactttcgatgcttacaaattgaaaatatttaaatcacaataatattta
cattttcaaaaaaaaaaaaaaaacatagattggtttattgaaaggtcacaatgctctttagaatttttttatcata
attagaaacaatcaataatttataactgccaaaaaaaggtatacacagctctgtatttcataaagtgcatatttc
aatacgcttgcttatgtaatttttcaacaagctggcattacattgttaacccgtgtccctaagggcgaattctgc
agatatccatcacactggcggccgc

TTAA > GGCC

col-dAp D POU-CRM

ggatccactagtaacggccgccagtgtgctggaattcgcccttcattttcgtgcgtctcatcgattcggtgacca
agcaggtgagttcaaaaatatttcaattagttatggatgaactcaacataaattataagaaatggtgctataact
agcaacggaacatacatgtttagaaaattagaaaatcataaataatatacattaataacaactcaagttttaatc
ctttacacaacttgaaagaagtaatgcccaaacatctaatttgaataggttgagtaatttcctgcacagaaaact
cgaaacaacttcaaaaccacttaaatatatttcgaaaattagcaattcatcttgcgattgccacttacataaatt
aagaacttcctgattgccacgctgcttcactgcaaattaaggccaaagttaaCCAggCTAcGaAAAAccAATTCC
TTtCACTtGGCTGcttccgtgcattttccctctttcccggacacgaTGTTTGTTTTGCTGATTCGAGCATAAGTT
GCATTCGAAATTGATTGTCAtGGCTAATTAAGTGtGTCCTGGtGTGTCCTtTGGCCaATAAATTGAGtCGTCaAA
CGAGCAgctcgttcggccaggacttttccgacttttccgcgtgaaaaaAAAGGGGTGTgaCCGgggctaacgaga
gaagcgagcaAATCGAGAGAAtcgcacggggatcgaacaTtGTTAATGCGCTtTGTTGTGGGTGCGTGCagGTCC
TggggcattatactggtccttatagtccTGcTGCACCATATGTccGCTTTTGTcGgCctaatgccctgcgagtcc
tttttcttgcctCTcCtTTTTcggCCTTTTCAAGGGCAATTAATTTCTCGAGTGACAAATTTTTATAGACattgg
acgactcctttcgccgtctcttccccattaacccccccccctctctctctctcactccaatcggtggcactact

tTCTCgaTTTctCTtcgtTTTTGTGGGCGTTcCtTGCCCaTTTAGCCCgTTCGcATCAGAAAaCGAGagCGCAAT
AAATTTgTCCACAAATTATCGACAGCatGcGACATCtcgcCCCCtTTtctttactttatggtgacaaatccattt
gtgcgaatgcaATTTAATCGCAAAAttatttgagcagccaccaattgaatttgctaaaaatatcacacacgaaaa
aaaaatggccgtaaaagaaaaggtctctgtgaagttttgtttaattttttttttaggctttgccgataagaagat
tatttatgggcattgttttaggcaattaaagcttatcgacaaattgaatagcgccatagagagttactttaatat
agcaaatgagtaattacaagcagttTAATGGGtCTTcaaggaaacatacgaattcccgtgctaaatgcgacagat
tcccaaatggatactcattgtacaactcactcaagtttcaatttcacaataatcgaaatacgaatcgggaagtaa
aacatacatataagtatggatgttatattttctaagaataactattacatcccggttagttccttcctccgattt
cccTaAAtTgcCCAttTtcgcttgacttgtggcgtatttttgatagacCTACCCCTAAacctgggaaaatcacac
agctctgcgagttttgtgcccatttgaaaagtgtcgcaagcTGGGaaaagaaCAATCAttTGATCgGATTAGGtG
GGTTcggctgcctctttgtagaccctttgttttcgactggatgtttttgaaTTTTacATTTcgctTGTCGCGTGt
cgcttttctTTATCGgattGCGTAACAGGTAAGGCGGGGATAAGCGaGTGTTATCtttagcaaGTGGGattgcaa
ctcgaaactcgaaACTgggAACTGTgGCgggccGAATgCGATTAAggcGGaATtGTCTCAatcgcaaatggcagg
aagGCAGGACAATTGTcaactgccatcCACGTGGCaggaccaaagaaaaaaaaaatgaaaatccaaaaaacaagc
agcgacgaggcacaacccttgcaACCCCTTTcctTTcGGGCaAATGcagcAgcAAATCATGGtCATAATCATAAT
TACATTCGATCAGATAACgGccaaaAACaGTTCGAACgGGAGGCGTCGCAATTGCAATTTGAGCTAACGAtGgcA
cCACGGAAaaTCGCcTCATTTcgGCGATCATATTTCATTTTCATCagcggatggtggatagatagactggtgcta
taagggtccgGCATaAAACAAtaGcCAAATCCtTTtgCCCggTTTTTgTGTTTTGCCAAAATTGcgCTTATCgCG
ATTaaaACGCAcgcacAAAAGCGCAtCGAAATtCTAAACCaaAAAGAGAtggatagcgttcgaggggcagagagg
agagacggcagataactggcGATTAATGCActgaGATAActgagttatagtggttgaaaggagcccaaaaatcca
ggcgaactactactaatgacaaagatccgtcaaccggttacccgatttatccaaccgctaacggttaaaacgaaa
atcggaaagcagcatcattaaataccctatagccattgcactcgcacacacacacacacacactcgcacacat
gctgtTGTCTGCGcgTgcTAATTAAGcGATAAAATTAATTTGTaTGTCAACACTTAAAAcGCcaaaagaGAGAGa
ATCATTTTCGATTAtTAACAaATTgtacgagcacaGCTCGcttaatgGCAAATgGTTATTGTTGTTGtcgctggc
gccGCCGGCCaCTCAATAAATTCAACAGATTGCGcGAAATCTCCaCATTTGCATgtgcgccgccctccgtccgtc
ccgctcgcaccagcccgatttatggccctccccacgctggCTCTCCCtttcGCACGCAATGCGATTAAAAATGCt
tttaTTTCACGGCAAGTGCATTTCTGCGGTcgaaagagatggcgtaaagagcgagagagtgagtgagaaaacagg
cggaaaaatgATATCGtCCTCATTATGCGAAATGTGtAATGCAtGCGgcatgctcCCCTTTgAAAtTCTTttggc
tcccttggggaacttcggttttataaaggtgtgccaaatgcactgccctatgtgggtgaaagcaatcttaactt
taaataaatgagcaacgtaggttacatactttcgatgcttacaaattgaaaatatttaaatcacaataatattta
cattttcaaaaaaaaaaaaaaaacatagattggtttattgaaaggtcacaatgctctttagaatttttttatcata
attagaaacaatcaataatttataactgccaaaaaaaggtatacacagctctgtatttcataaagtgcatatttc
aatacgcttgcttatgtaatttttcaacaagctggcattacattgttaacccgtgtccctaagggcgaattctgc
agatatccatcacactggcggccgc

gtaattac > TGCCGGCA on the plus and on the minus strand double site
ATAATCATAATTAC > CGCCGACGCCGGCA on the plus and minus strand double site

Atttgaata : initial attt is from the reverse octamer like sequence which can be bound by POU as well. ATTCAAAT att sites will be converted to CGGG

TATGC/GCATA > GCGTA
TATTC/GAATA > GCGGA
ATGCAAAT/ATTTGCAT > CGGGTACG
Ataatta > CGCCGGC

col-dAp D GATA-CRM

ggatccactagtaacggccgccagtgtgctggaattcgcccttcattttcgtgcgtctcatcgattcggtgacca
agcaggtgagttcaaaaatatttcaattagttatggatgaactcaacataaattataagaaatggtgctataact
agcaacggaacatacatgtttagaaaattagaaaatcataaataatatacattaataacaactcaagttttaatc
ctttacacaacttgaaagaagtaatgcccaaacatctaatttgaataggttgagtaatttcctgcacagaaaact
cgaaacaacttcaaaaccacttaaatatatttcgaaaattagcaattcatcttgcgattgccacttacataaatt
aagaacttcctgattgccacgctgcttcactgcaaattaaggccaaagttaaCCAggCTAcGaAAAAccAATTCC
TTtCACTtGGCTGcttccgtgcattttccctctttcccggacacgaTGTTTGTTTTGCTGATTCGAGCATAAGTT
GCATTCGAAATTGATTGTCAtGGCTAATTAAGTGtGTCCTGGtGTGTCCTtTGGCCaATAAATTGAGtCGTCaAA
CGAGCAgctcgttcggccaggacttttccgactttccgcgtgaaaaaAAAGGGGTGTgaCCGgggctaacgaga
gaagcgagcaAATCGAGAGAAtcgcacggggatcgaacaTtGTTAATGCGCTtTGTTGTGGGTGCGTGCagGTCC
TggggcattatactggtccttatagtccTGcTGCACCATATGTccGCTTTTGTcGgCctaatgccctgcgagtcc
tttttcttgcctCTcCtTTTTcggCCTTTTCAAGGGCAATTAATTTCTCGAGTGACAAATTTTTATAGACattgg
acgactcctttcgccgtctcttccccattaaccccccccccctctctctctctcactccaatcggtggcactact
tTCTCgaTTTctCTtcgtTTTTGTGGGCGTTcCtTGCCCaTTTAGCCCgTTCGcATCAGAAAaCGAGagCGCAAT

AAATTTgTCCACAAATTATCGACAGCatGcGACATCtcgcCCCCtTTtctttactttatggtgacaaatccattt
gtgcgaatgcaATTTAATCGCAAAAttatttgagcagccaccaattgaatttgctaaaaatatcacacacgaaaa
aaaaatggccgtaaaagaaaaggtctctgtgaagtttgtttaatttttttttttaggctttgccgataagaagat
tatttatgggcattgttttaggcaattaaagcttatcgacaaattgaatagcgccatagagagttactttaatat
agcaaatgagtaattacaagcagttTAATGGGtCTTcaaggaaacatacgaattcccgtgctaaatgcgacagat
tcccaaatggatactcattgtacaactcactcaagtttcaatttcacaataatcgaaatacgaatcgggaagtaa
aacatacatataagtatggatgttatatttctaagaataactattacatcccggttagttccttcctccgattt
cccTaAAtTgcCCAttTtcgcttgacttgtggcgtattttttgatagacCTACCCCTAAacctgggaaaatcacac
agctctgcgagttttgtgcccatttgaaaagtgtcgcaagcTGGGaaaagaaCAATCAttTGATCgGATTAGGtG
GGTTcggctgcctctttgtagaccctttgttttcgactggatgtttttgaaTTTTacATTTcgctTGTCGCGTGt
cgcttttctTTATCGgattGCGTAACAGGTAAGGCGGGGATAAGCGaGTGTTATCtttagcaaGTGGGattgcaa
ctcgaaactcgaaACTgggAACTGTgGCgggccGAATgCGATTAAggcGGaATtGTCTCAatcgcaaatggcagg
aagGCAGGACAATTGTcaactgccatcCACGTGGCaggaccaaagaaaaaaaaaatgaaaatccaaaaaacaagc
agcgacgaggcacaacccttgcaACCCCTTTcctTTcGGGCaAATGcagcAgcAAATCATGGtCATAATCATAAT
TACATTCGATCAGATAACgGccaaaAACaGTTCGAACgGGAGGCGTCGCAATTGCAATTTGAGCTAACGAtGgcA
cCACGGAAaaTCGCcTCATTTcgGCGATCATATTTCATTTTCATCagcggatggtggatagatagactggtgcta
taagggtccgGCATaAAACAAtaGcCAAATCCtTTtgCCCggTTTTTgTGTTTTGCCAAAATTGcgCTTATCgCG
ATTaaaACGCAcgcacAAAAGCGCAtCGAAATtCTAAACCaaAAAGAGAtggatagcgttcgaggggcagagagg
agagacggcagataactggcGATTAATGCActgaGATAActgagttatagtggttgaaaggagcccaaaaatcca
ggcgaactactactaatgacaaagatccgtcaaccggttacccgatttatccaaccgctaacggttaaaacgaaa
atcggaaagcagcatcattaaataccctatagccattgcactcgcacacacacacacacacacactcgcacacat
gctgtTGTCTGCGcgTgcTAATTAAGcGATAAAATTAATTTGTaTGTCAACACTTAAAAcGCcaaaagaGAGAGa
ATCATTTTCGATTAtTAACAaATTgtacgagcacaGCTCGcttaatgGCAAATgGTTATTGTTGTTGtcgctggc
gccGCCGGCCaCTCAATAAATTCAACAGATTGCGcGAAATCTCCaCATTTGCATgtgcgccgccctccgtccgtc
ccgctcgcaccagcccgatttatggccctccccacgctggCTCTCCCtttcGCACGCAATGCGATTAAAAATGCt
tttaTTTCACGGCAAGTGCATTTCTGCGGTcgaaagagatggcgtaaagagcgagagagtgagtgagaaaacagg
cggaaaaatgATATCGtCCTCATTATGCGAAATGTGtAATGCAtGCGgcatgctcCCCTTTgAAAtTCTTttggc
tcccttggggaacttcggttttataaaggtgtgccaaatgcactgccctatgtgggtgaaagcaatcttaactt
taaataaatgagcaacgtaggttacatactttcgatgcttacaaattgaaaatatttaaatcacaataatattta
catttcaaaaaaaaaaaaaaaaacatagattggtttattgaaaggtcacaatgctctttagaattttttatcata
attagaaacaatcaataatttataactgccaaaaaaaggtatacacagctctgtatttcataaagtgcatatttc
aatacgcttgcttatgtaatttttcaacaagctggcattacattgttaacccgtgtccctaagggcgaattctgc
agatatccatcacactggcggccgc

GATA-binding AGATAA and TGATAA
GATA > TCGC
TATC > GCGA

col-Tv-CRM

gcggccgctgatagaagtagggtatttctaacaagtacaattaaatagacatatgtacttttttcagtaaaatat
taccaattttcacgcactacttaagccaatctaccaccttaattccgtttttttttcttttgaacacatgaaaaa
ATTCAATTccttaGAtTTAtATTAAtCtaATtgAaAtCCacaAATTTGtTGCCATTTgTTgtgcagAAgTATTTT
TGAATTTcGCACACGTTcgtTGgCAAAAAtatgtacaaatattgAATacGAAaAAgtcaaGGAaAAAttccattc
tTTTtctCGCaaAAATgGTGACaGAGCaAAatgctgaccgcCGCCTACTaGGAAAAATAaTCCCCAGcCGATTtg
aACGATTTATTATTTGGccCAGCCaactcggagcctgtcccggaatacccGGAACCATTAATCATcCGAGTccaG
ATCCaAGTCCgagtccgagtggtggacaggcggcattgcaattggtggacatTTtGGagtgGAAgAAAcTTTTGC
GGtACGTGGCAACGTGTTGAAATTACAAAATGTGTTAAAAAaTGaaagagcagggcgggtaaaaagcAAAATAAt
ATATTTcattCACACACAGACttttgtctctgctccgccgtccatatAAAAAACATAAATTTCCTTTGAGAATAG
AacttctgggatggagagggggggggggggggtgCAAGGTGATTATgTTTGACAGCCGACttGGGAcacgaaGcac
CGCCCccTTtttcatcGCCAaACAGGACAtGGgtattAGaTCCTGgccaaagtgccaaaactggcctcactttcc
GtCTGATTTAcggccaactatttgtttaaattttaccacttccctgctatcgacactcaacttaaaacatata
aaaccaactatttaaagagcaaataaagttcaatttgatttctttggccttcgttaataattttcctgcatctc
tctgGGGAtcaGcgaAAAATATGAAttaTTgAAACAGCTGAAggaaatAATAtaaCCAAATcggaaggccaaatc
tgtttaatgtttcagatatgttggtgtgggttgaccaaatgacaatatgctttttagCtTcAACCTCCCTCAAgc
gAAATTGATTTTCAtggtttttctgttttttcgcctaaaaatACAcAAAAgagtcgAaAAAatttgactgtgatt
gtcacttgaatgtgtgggttgggaaAACCAcTCcAaATCCACttcaccaaatatgtcagtaTGGaATCACGAacc
tcGctTGATTTCTCCAGTGATTTTTCGCAATCAGccTGATGAGGATGTGCcgagaacgttgctCaTGgagGCagT
gCTATGATTAGTATCCTttcagccaggtcaaaagtgacgctgccGCATGCCAAGTGGCCtcggcCaaATGAAACT
TTAATGcgaaacaaggacaaaaggctgaaagaaggctgcctgatgaactacgtgatggtcagacaaattgaagtt
acactggaaaataattagctaatttcctagtttgcttaggttaagggattaagtaagaatgtgtttaaaggactc
ttttataggtaacttgttactctaaagaaaacttatccacagaatagatgtgctattttatcgcagtgtatgtgg

ataGtCAacGACAAAGTGaAATGcaTTGAGGttggtggcgaacaaacgcgacctgaatcgcgaattggaatagcc
agcctactccgccccctcaggatatatagctacttcgactagcatatgcgccagatagtatagctggcgtatcaa
tttcgataatgtacttggaccgcgaGtATCTTTATTaTCCTgacTGCTGtGCAATTATTtccgactgctttactg
tcgtaccgcttttccgagTCCTGGGAAAcGCAAACAAaGGatATTTCGcGCACACGcCtcggacATGATTGCATC
CGggcaGGAAGCatTgcgtCGTGTcctttttGCATCCtTACATcgtcgCTtCCTTCCtgccgatcagaacccctg
gctgcttTTCATCCGCAATTTTCACGCCCACTTTGTTTGATGATGACAAATCTCGTTGCGTGTTATCAGATTGGC
caactttccgttgctattccccctcgtATtGCATAATTATGTTTATCTTtGGAagctgcgctcctgccacgtgtc
cttgcgttttggttagcttcctgcgattttGCTcGGAacgAGtGgCACGTGCTAATCGGCGGTCGtctgcacCGC
CCatgcaaccagccaccccccctggtaagtgcaaCCCCGCctaACAtgtCCGATAATTGAAATCGCTAAGCtgaa
caacggaatcgaaatggcttcattaatcaggtcacttcgatggcagcgcataatgttcttcaaaaatttgaattg
ggggcggttgaaaagaggaaattggtctatatagcacttagcgcgattgaatttgctcattttgtgttacctaat
tggggaaaagatgtttcggttattaatcatgagtgatacttactattgtattacaaaaatggtgcatatatcaga
agatatatattattaatagatcttaaggagcatttgtatccattcaccaaaatatggtaaaaataaagcttaaat
taaatttgctcatttacctTTAAggacatcctttgctTAATGAGTGCaCTCAtgcccacacccactcgcaacCGA
ATGAGTGTCGCCCTGcGGACGCATAAATTATaAATAAAAgCCtCaCACACGTGGAGAaagagaggtgtatataac
cttagagagagagagagagagggagagctaCAGTCTGAGGCCaAGaAGTGTgCTAAATATTTAAgtACTtAAAACCA
TAAACCTGAGTATTTATAAATTggctttaacaagctcgtatgtgacggtggttgggtgtgtgtctgtgggCtGAC
CCTTTTttcGGgcacaaggatctgctcgactcgattccatTCATtAGAAATGCtcACTAATGTGCCCGaCTTAAT
AATAATCAAATTTATATGCATGGTTATGTCCTTtAgagcaggaccacaaaactgaagtttcgagtggattgcgaa
tgggattggctgttaagtttacggagaaacctttgagaatgagacgaagataataTGTCAATATCCTTGttttcc
aaaatgtctgaccaaaagtgcagatgtccagtatcgaaacacgcccaagaccggtaccgttattctgctaccagt
tttcttgtttgtttttgtttgttagccagcttgtttgtggccgacactgagaccaaaaaggccaaaaaggtcgcc
ccctcgggatttccttagtcctgctgccagcttcttccttccacattctttttgggggtccGATGAGatTTGaAA
aTttcACAAAAATTAATTAggaatgctggcatccatgttgtgataggctagttcttttctagcctagcaacattt
ttgatgtgactaaagagaatcaTCTTTaTGGCCAcacccggaatactcattcttcaaacaacaagcgacaataaa
aacaaattacaattgcaacaatggcagcggcagcaaaacatttgagacattttccccacacacacacacagacac
acacatacttcagttaaatgaacatttgcaggacctagatatttatttattacaggggttgcactgcaggagcat
taaccccttactgcccacggcagtctcgagcaacggaggactgaAAAAATTGAAATAAATTACCAAAACTAAAGG
CAACAACTGTTCGCATAATGAAGTCAAATTATATTTgTgCCTaagtgcactgagagaaatttaatataacctatg
atttacacgatcgattttcgatctttgcaacatttttgagtgaaactaggaaaccctttacaagcgagatcttg
cagttctttagtttctaagcattattctagtattttaaaatatattttcctttgttctggtcttaatttattata
actaatgaatattttccgtctgtgtattcggaaaaggggggagttggaacatataaagatggggtgcagaaaagat
gatcagggaaggtgtgagtggttcaaactagctgggcgtttggatagcgaAGGGTGcGTCCtttgcTGGTCCTTc
gAATTATGCAAATcgtcTGCACttcagatactactacccctacaaaaaaaaaaaaacgaagagaaaaccccctaca
aacaaacgtATTAGtGTCAtCgaGCATTtGCCaagCCATTtCaTtGACACgTagaacacctttttcgtccacttc
gtatGATGTTATCATTTAATTCtTGggGAATTAACACGGACATTGtCAaCcGTAAATGAGCTAATTAATCTAgTA
AATGTgTCGcagctcattccacaatttctctactacttgagagcgaggagtgggttcGCGTTTTTGTGGTTCTCC
AGAGGATCCCCAatGcTGACACGCTTCTCCAGgTGgccgaggagtgggaaatggaaaatGGGCaGGgtttgcCCC
tcggGGaGCTGCACtAaAACcCCAAATGAGATGCAtGAATTCctcttcgccgacaagcagctcataaaagttaac
atcgaagtatttacagcatattttggggaaacttcaacttggaatttgattaaatgcaaactacttgaaaacata
caacttcaaatcaagtatgtacaattttaaatcaagtagatctctttgaatttattgatttgcttaaccaactta
agtttccttaacttctcttattttttgtgtaTTTTGGCTAACTatTTTTGGCCAcAAatCgatctttggtcgcgcc
aaagttaatgaaccaaccgcacttttCCAATCTCGCAtttgatgcgttggattccacaTATTTGGcCAGactgaa
AAAtAATTTCaTGTTTATGGCcAcGTTGTTTTCACCgGcCACaAAAAtGCTTATCGAacgccgGAGATCgccaag
cgagagaCTTGTAAATATTTATGATTTTttgcgatttttattcgactgataaacgagtttttcccatcgcacagt
tgcgAtGTGcgaaaatgttttgtgggaagaaattgtgcacctagcccatacgattcgattcaccttctgtttgtt
ggAATAAtAAATCtGtAGAAaGGTtgttgtatTTTTagaagactccatccccctTTgTTaTTCTtAtGTAAACGA
AACCTTtttcctacacacatagctgcatagtaagttACCCTCAtGAGAACGTTTAGGccgtgaatatTGGGTAAA
TATTTAAACTGGTTgCGATTGTTTTgGCAAAATCGTTGCAGTTGGtCAAACACGTGTTTGAGATAATTtAATTAA
ATTTACTTGgCTTTTTTaacccCgTGCCACGTGCCACTcgagtccagtttgctgccctcgaaatggAGTGGCAAAC
ACTtGAGCACTTGCTttaaAAaTGcGGCAAGTGCAtttgggttgggtgggtagtttagcttggttttggctggct
ggtgaaaaTTCctagGAAtcGAAAACTTTGGTGGTCTtcggacgGCaGACAATGCAACaaTGTCatacgtactTG
GATgaatcgcTGGCATCtGTCgcagTCCGtgGAGAatAgaccgAaATTCCcCCGAActcacgagcATGAtgtggC
GATGATGACTtGtGgAAATCaaGCAatgatttccactccatCTGtAaCTAAATATGATTTATtTtCACGgtGTGt
gttagccgctctCgCCcCtCTCATTAGtcggggcaccggggTAATTTGCATAAGGTCCTGGCCCCAGGAaccAAA
ggACaCACtcgagagcgagctGGAaACTAATTAAAATGTTCGCGCcTCAAATTTCCttcgCtCGccCtTATTTAT
GaCCCTGTGAATGTCTtGCATATGGAcggagttgagatccTTCTcgttTTctTCCTTTTTTTGTTTACGAgTGAC
AAATGggACATTTACATGCgagtcagtTTGCATATtggAATTATTCATGGTTtCTttaaagatggcgcaggatgt
gatgtcgcgggaaaaggatgagggtttcctcaaggaagtcggaaatagaagtggtttcttttcatgctaccaata
tgggggcacataaaacttgcatttgatgagctaaatgtaaacatacactatttaaatacatttttatgataagt
gaacttaaagtcacactaactttgaaaatttgattgacttctacaatgtgtgttgtttaatttcttattttata

ttttttaaatatcgaaaatctacaaatccgcttatgtttaaaagtcaagccgctggctaattgacaaaatgtgt
aattgtggcgatgagagtccttccgattgtcactctcccaaccctccgtaatcccctaagtcaccataggggttg
ggtaaaaatctaga

col-Tv D Cas-CRM

gcggccgctgatagaagtagggtatttctaacaagtacaattaaatagacatatgtactttttttcagtaaaatat
taccaattttcacgcactacttaagccaatctaccaccttaattccgtttttttttctttgaacacatgaaaaa
ATTCAATTccttaGAtTTAtATTAAtCtaATtgAaAtCCacaAATTTGtTGCCATTTgTTgtgcagAAgTAggcc
TGAATTTcGCACACGTTcgtTGgCccggAtatgtacaaatattgAATacGAAaAAgtcaaGGAaAAAttccattc
tTTTtctCGCaaAAATgGTGACaGAGCaAAatgctgaccgcCGCCTACTaGGccggATAaTCCCCAGcCGATTtg
aACGATTTATTATTTGGccCAGCCaactcggagcctgtcccggaataccCGGAACCATTAATCATcCGAGTccaG
ATCCaAGTCCgagtccgagtggtggacaggcggcattgcaattggtggacatTTtGGagtgGAAgAAAcTTTTGC
GGtACGTGGCAACGTGTTGAAATTACAAAATGTGTTccggAaTGaaagagcagggcgggtaaaaagcAAAATAAt
ATATTTcattCACACACAGACttttgtctctgctccgccgtccatatccggAACATAAATTTCCTTTGAGAATAG
AacttctgggatggagaggggggggggggggtgCAAGGTGATTATgTTTGACAGCCGACttGGGAcacgaaGcac
CGCCCccTTtttcatcGCCAaACAGGACAtGGgtattAGaTCCTGgccaaagtgccaaaactggcctcactttcc
GtCTGATTTAcggccaactatttgtttaaatttttaccacttccctgctatcgacactcaacttaaaacatata
aaaccaactatttaaagagcaaataaagttcaatttgatttctttggccttcgttaataattttcctgcatctc
tctgGGGAtcaGcgaAAAATATGAAttaTTgAAACAGCTGAAggaaatAATAtaaCCAAATcggaaggccaaatc
tgtttaatgtttcagatatgttggtgtgggttgaccaaatgacaatatgcttttagCtTcAACCTCCCTCAAgc
gAAATTGATTTTCAtggtttttctgtttttcgcctaaaaatACAcAAAAgagtcgAaAAAatttgactgtgatt
gtcacttgaatgtgtgggttgggaaAACCAcTCcAaATCCACttcaccaaatatgtcagtaTGGaATCACGAacc
tcGctTGATTTCTCCAGTGATggccCGCAATCAGccTGATGAGGATGTGCcgagaacgttgctCaTGgagGCagT
gCTATGATTAGTATCCTttcagccaggtcaaaagtgacgctgccGCATGCCAAGTGGCCtcggcCaaATGAAACT
TTAATGcgaaacaaggacaaaaggctgaaagaaggctgcctgatgaactacgtgatggtcagacaaattgaagtt
acactggaaaataattagctaatttcctagtttgcttaggttaagggattaagtaagaatgtgtttaaaggactc
ttttataggtaacttgttactctaaagaaaacttatccacagaatagatgtgctattttatcgcagtgtatgtgg
ataGtCAacGACAAAGTGaAATGcaTTGAGGttggtggcgaacaaacgcgacctgaatcgcgaattggaatagcc
agcctactccgcccccctcaggatatatagctacttcgactagcatatgcgccagatagtatagctggcgtatcaa
tttcgataatgtacttggaccgcgaGtATCTTTATTaTCCTgacTGCTGtGCAATTATTtccgactgctttactg
tcgtaccgcttttccgagTCCTGGGAAAcGCAAACAAaGGatATTTCGcGCACACGcCtcggacATGATTGCATC
CGggcaGGAAGCatTgcgtCGTGTccttttttGCATCCtTACATcgtcgCTtCCTTCCtgccgatcagaacccctg
gctgcttTTCATCCGCAATTTTCACGCCCACTTTGTTTGATGATGACAAATCTCGTTGCGTGTTATCAGATTGGC
caactttccgttgctattcccctcgtATtGCATAATTATGTTTATCTTtGGAagctgcgctcctgccacgtgtc
cttgcgttttggttagcttcctgcgattttGCTcGGAacgAGtGgCACGTGCTAATCGGCGGTCGtctgcacCGC
CCatgcaaccagccaccccccctggtaagtgcaaCCCCGCctaACAtgtCCGATAATTGAAATCGCTAAGCtgaa
caacggaatcgaaatggcttcattaatcaggtcacttcgatggcagcgcataatgttcttcaaaaatttgaattg
ggggcggttgaaaagaggaaattggtctatatagcacttagcgcgattgaatttgctcattttgtgttacctaat
tggggaaaagatgtttcggttattaatcatgagtgatacttactattgtattacaaaaatggtgcatatatcaga
agatatatattattaatagatcttaaggagcatttgtatccattcaccaaaatatggtaaaaataaagcttaaat
taaatttgctcatttacctTTAAggacatcctttgctTAATGAGTGCaCTCAtgcccacacccactcgcaacCGA
ATGAGTGTCGCCCTGcGGACGCATAAATTATaAATAAAAgCCtCaCACACGTGGAGAaagagaggtgtatataac
cttagagagagagagagagggagagctaCAGTCTGAGGCCaAGaAGTGTgCTAAATATTTAAgtACTtAAAACCA
TAAACCTGAGTATTTATAAATTggctttaacaagctcgtatgtgacggtggttgggtgtgtgtctgtgggCtGAC
CCTTTTttcGGgcacaaggatctgctcgactcgattccatTCATtAGAAATGCtcACTAATGTGCCCGaCTTAAT
AATAATCAAATTTATATGCATGGTTATGTCCTTtAgagcaggaccacaaaactgaagtttcgagtggattgcgaa
tgggattggctgttaagtttacggagaaacctttgagaatgagacgaagataataTGTCAATATCCTTGttttcc
aaaatgtctgaccaaaagtgcagatgtccagtatcgaaacacgcccaagaccggtaccgttattctgctaccagt
tttcttgtttgtttttgtttgttagccagcttgtttgtggccgacactgagaccaaaaaggccaaaaaggtcgcc
ccctcgggatttccttagtcctgctgccagcttcttccttccacattctttttggggggtccGATGAGatTTGaAA
aTttcACccggATTAATTAggaatgctggcatccatgttgtgataggctagttcttttctagcctagcaacattt
ttgatgtgactaaagagaatcaTCTTTaTGGCCAcacccggaatactcattcttcaaacaacaagcgacaataaa
aacaaattacaattgcaacaatggcagcggcagcaaaacatttgagacattttccccacacacacacacagacac
acacatacttcagttaaatgaacatttgcaggacctagatatttatttattacaggggttgcactgcaggagcat
taaccccttactgcccacggcagtctcgagcaacggaggactgaAAAAATTGAAATAAATTACCAAAACTAAAGG
CAACAACTGTTCGCATAATGAAGTCAAATTATATTTgTgCCTaagtgcactgagagaaatttaatataacctatg
atttacacgatcgatttcgatctttgcaacatttttgagtgaaactaggaaaccctttacaagcgagatcttg
cagttctttagtttctaagcattattctagtattttaaaatatattttcctttgttctggtcttaatttattata
actaatgaatattttccgtctgtgtattcggaaaaggggagttggaacatataaagatggggtgcagaaaagat
gatcagggaaggtgtgagtggttcaaactagctgggcgtttggatagcgaAGGGTGcGTCCtttgcTGGTCCTTc
gAATTATGCAAATcgtcTGCACttcagatactactaccccctacaaaaaaaaaaaacgaagagaaaacccccctaca

aacaaacgtATTAGtGTCAtCgaGCATTtGCCaagCCATTtCaTtGACACgTagaacacctttttcgtccacttc
gtatGATGTTATCATTTAATTCtTGggGAATTAACACGGACATTGtCAaCcGTAAATGAGCTAATTAATCTAgTA
AATGTgTCGcagctcattccacaatttctctactacttgagagcgaggagtgggttcGCGggccTGTGGTTCTCC
AGAGGATCCCCAatGcTGACACGCTTCTCCAGgTGgccgaggagtgggaaatggaaaatGGGCaGGgtttgcCCC
tcggGGaGCTGCACtAaAACcCCAAATGAGATGCAtGAATTCctcttcgccgacaagcagctcataaaagttaac
atcgaagtatttacagcatattttggggaaacttcaacttggaatttgattaaatgcaaactacttgaaaacata
caacttcaaatcaagtatgtacaattttaaatcaagtagatctctttgaatttattgatttgcttaaccaactta
agtttccttaacttctcttattttgtgtaTTTTGGCTAACTatTTTTGGCCAcAAatCgatctttggtcgcgcc
aaagttaatgaaccaaccgcacttttCCAATCTCGCAtttgatgcgttggattccacaTATTTGGcCAGactgaa
AAAtAATTTCaTGTTTATGGCcAcGTTGTTTTCACCgGcCACaAAAAtGCTTATCGAacgccgGAGATCgccaag
cgagagaCTTGTAAATATTTATGATTTTttgcgatttttattcgactgataaacgagtttttcccatcgcacagt
tgcgAtGTGcgaaaatgttttgtgggaagaaattgtgcacctagcccatacgattcgattcaccttctgtttgtt
ggAATAAtAAATCtGtAGAAaGGTtgttgtatTTTTagaagactccatccccctTTgTTaTTCTtAtGTAAACGA
AACCTTtttcctacacacatagctgcatagtaagttACCCTCAtGAGAACGTTTAGGccgtgaatatTGGGTAAA
TATTTAAACTGGTTgCGATTGTTTTgGCAAAATCGTTGCAGTTGGtCAAACACGTGTTTGAGATAATTtAATTAA
ATTTACTTGgCTggccTaaccCgTGCCACGTGCCACTcgagtccagtttgctgccctcgaaatggAGTGGCAAAC
ACTtGAGCACTTGCTttaaAAaTGcGGCAAGTGCAtttgggttgggtgggtagtttagcttggttttggctggct
ggtgaaaaTTCctagGAAtcGAAAACTTTGGTGGTCTtcggacgGCaGACAATGCAACaaTGTCatacgtactTG
GATgaatcgcTGGCATCtGTCgcagTCCGtgGAGAatAgaccgAaATTCCcCCGAActcacgagcATGAtgtggC
GATGATGACTtGtGgAAATCaaGCAatgatttccactccatCTGtAaCTAAATATGATTTATtTtCACGgtGTGt
gttagccgctctCgCCcCtCTCATTAGtcggggcaccggggTAATTTGCATAAGGTCCTGGCCCCAGGAaccAAA
ggACaCACtcgagagcgagctGGAaACTAATTAAAATGTTCGCGCcTCAAATTTCCttcgCtCGccCtTATTTAT
GaCCCTGTGAATGTCTtGCATATGGAcggagttgagatccTTCTcgttTTctTCCTTTggccGTTTACGAgTGAC
AAATGggACATTTACATGCgagtcagtTTGCATATtggAATTATTCATGGTTtCTttaaagatggcgcaggatgt
gatgtcgcgggaaaaggatgagggtttcctcaaggaagtcggaaatagaagtggtttcttttcatgctaccaata
tgggggcacataaaacttgcatttgatgagctaaatgtaaacatacactatttaaatacatttttatgataagt
gaacttaaagtcacactaacttttgaaaatttgattgacttctacaatgtgtgttgtttaatttcttattttata
ttttttaaatatcgaaaatctacaaatccgcttatgtttaaaagtcaagccgctggctaattgacaaaatgtgt
aattgtggcgatgagagtccttccgattgtcactctcccaaccctccgtaatcccctaagtcaccataggggttg
ggtaaaaatctaga

Brown: Cas TTTT > GGCC   AAAA > CCGG   Sites converted in total: 10

col-Tv D Exd-CRM

gcggccgctgatagaagtagggtatttctaacaagtacaattaaatagacatatgtactttttcagtaaaatat
taccaattttcacgcactacttaagccaatctaccaccttaattccgtttttttcttttgaacacatgaaaaa
ATTCAATTccttaGAtTTAtATTAAtCtaATtgAaAtCCacaAATTTGtTGCCATTTgTTgtgcagAAgTATTTT
TGAATTTcGCACACGTTcgtTGgCAAAAAtatgtacaaatattgAATacGAAaAAgtcaaGGAaAAAttccattc
tTTTtctCGCaaAAATgGTGACaGAGCaAAatgctgaccgcCGCCTACTaGGAAAAATAaTCCCCAGcCGATTtg
aACGATTTATTATTTGGccCAGCCaactcggagcctgtcccggaatacccGGAACCATTAccggTcCGAGTccaG
ATCCaAGTCCgagtccgagtggtggacaggcggcattgcaattggtggacatTTtGGagtgGAAgAAAcTTTTGC
GGtACGTGGCAACGTGTTGAAATTACAAAATGTGTTAAAAAaTGaaagagcagggcgggtaaaaagcAAAATAAt
ATATTTcattCACACACAGACttttgtctctgctccgccgtccatatAAAAAACATAAATTTCCTTTGAGAATAG
AacttctgggatggagaggggggggggggtgCAAGGccggTATgTTTGACAGCCGACttGGGAcacgaaGcac
CGCCCccTTtttcatcGCCAaACAGGACAtGGgtattAGaTCCTGgccaaagtgccaaaactggcctcactttcc
GtCTGATTTAcggccaactattttgtttaaatttttaccacttccctgctatcgacactcaacttaaaacatata
aaaccaactatttaaagagcaaataaagttcaatttgattttctttggccttcgttaataattttcctgcatctc
tctgGGGAtcaGcgaAAAATATGAAttaTTgAAACAGCTGAAggaaatAATAtaaCCAAATcggaaggccaaatc
tgtttaatgtttcagatatgttggtgtgggttgaccaaatgacaatatgctttttagCtTcAACCTCCCTCAAgc
gAAATccggTTTCAtggttttctgtttttcgcctaaaaatACAcAAAAgagtcgAaAAAatttgactgtgatt
gtcacttgaatgtgtgggttgggaaAACCAcTCcAaATCCACttcaccaaatatgtcagtaTGGaccggCGAacc
tcGctccggTTCTCCAGccggTTTTCGCAccggGccTGATGAGGATGTGCcgagaacgttgctCaTGgagGCagT
gCTAccggTAGTATCCTttcagccaggtcaaaagtgacgctgccGCATGCCAAGTGGCCtcggcCaaATGAAACT
TTAATGcgaaacaaggacaaaaggctgaaagaaggctgcctgatgaactacgtgatggtcagacaaattgaagtt
acactggaaaataattagctaatttcctagtttgcttaggttaagggattaagtaagaatgtgtttaaaggactc
ttttataggtaacttgttactctaaagaaaacttatccacagaatagatgtgctattttatcgcagtgtatgtgg
ataGtCAacGACAAAGTGaAATGcaTTGAGGttggtggcgaacaaacgcgacctgaatcgcgaattggaatagcc
agcctactccgccccctcaggatatatagctacttcgactagcatatgcgccagatagtatagctggcgtatcaa
tttcgataatgtacttggaccgcgaGtATCTTTATTaTCCTgacTGCTGtGCAATTATTtccgactgctttactg
tcgtaccgcttttccgagTCCTGGAAAcGCAAACAAaGGatATTTCGcGCACACGcCtcggacAccggTGCATC

CGggcaGGAAGCatTgcgtCGTGTcctttttGCATCCtTACATcgtcgCTtCCTTCCtgccgatcagaacccctg
gctgcttTTCATCCGCAATTTTCACGCCCACTTTGTTccggGATGACAAATCTCGTTGCGTGTTccggGATTGGC
caactttccgttgctattcccctcgtATtGCATAATTATGTTTATCTTtGGAagctgcgctcctgccacgtgtc
cttgcgttttggttagcttcctgcgattttGCTcGGAacgAGtGgCACGTGCTAATCGGCGGTCGtctgcacCGC
CCatgcaaccagccacccccctggtaagtgcaaCCCCGCctaACAtgtCCGATAATTGAAATCGCTAAGCtgaa
caacggaatcgaaatggcttcattaatcaggtcacttcgatggcagcgcataatgttcttcaaaaatttgaattg
ggggcggttgaaaagaggaaattggtctatatagcacttagcgcgattgaatttgctcattttgtgttacctaat
tggggaaaagatgtttcggttattaatcatgagtgatacttactattgtattacaaaaatggtgcatatatcaga
agatatatattattaatagatcttaaggagcatttgtatccattcaccaaaatatggtaaaaataaagcttaaat
taaatttgctcatttacctTTAAggacatcctttgctTAATGAGTGCaCTCAtgcccacacccactcgcaacCGA
ATGAGTGTCGCCCTGcGGACGCATAAATTATaAATAAAAgCCtCaCACACGTGGAGAaagagaggtgtatataac
cttagagagagagagagagggagagctaCAGTCTGAGGCCaAGaAGTGTgCTAAATATTTAAgtACTtAAAACCA
TAAACCTGAGTATTTATAAATTggctttaacaagctcgtatgtgacggtggttgggtgtgtgtctgtgggCtGAC
CCTTTTttcGGgcacaaggatctgctcgactcgattccatTCATtAGAAATGCtcACTAATGTGCCCGaCTTAAT
AATAccggAATTTATATGCATGGTTATGTCCTTtAgagcaggaccacaaaactgaagtttcgagtggattgcgaa
tgggattggctgttaagtttacggagaaacctttgagaatgagacgaagataataTGTCAATATCCTTGttttcc
aaaatgtctgaccaaaagtgcagatgtccagtatcgaaacacgcccaagaccggtaccgttattctgctaccagt
tttcttgtttgtttttgtttgttagccagcttgtttgtggccgacactgagaccaaaaaggccaaaaaggtcgcc
ccctcgggatttccttagtcctgctgccagcttcttccttccacattctttttggggtccGATGAGatTTGaAA
aTttcACAAAAATTAATTAggaatgctggcatccatgttgtgataggctagttcttttctagcctagcaacattt
ttgatgtgactaaagagaatcaTCTTTaTGGCCAcacccggaatactcattcttcaaacaacaagcgacaataaa
aacaaattacaattgcaacaatggcagcggcagcaaaacatttgagacattttccccacacacacacacagacac
acacatacttcagttaaatgaacatttgcaggacctagatatttatttattacaggggttgcactgcaggagcat
taaccccttactgcccacggcagtctcgagcaacggaggactgaAAAAATTGAAATAAATTACCAAAACTAAAGG
CAACAACTGTTCGCATAATGAAGTCAAATTATATTTgTgCCTaagtgcactgagagaaatttaatataacctatg
atttacacgatcgattttcgatctttgcaacatttttgagtgaaactaggaaaccctttacaagcgagatcttg
cagttctttagtttctaagcattattctagtattttaaaatatattttcctttgttctggtcttaatttattata
actaatgaatattttccgtctgtgtattcggaaaaggggagttggaacatataaagatggggtgcagaaaagat
gatcagggaaggtgtgagtggttcaaactagctgggcgtttggatagcgaAGGGTGcGTCCtttgcTGGTCCTTc
gAATTATGCAAATcgtcTGCACttcagatactactacccctacaaaaaaaaaaaacgaagagaaaacccccctaca
aacaaacgtATTAGtGTCAtCgaGCATTtGCCaagCCATTtCaTtGACACgTagaacacctttttcgtccacttc
gtatGATGTTccggTTTAATTCtTGggGAATTAACACGGACATTGtCAaCcGTAAATGAGCTAATTAATCTAgTA
AATGTgTCGcagctcattccacaatttctctactacttgagagcgaggagtgggttcGCGTTTTTGTGGTTCTCC
AGAGGATCCCCAatGcTGACACGCTTCTCCAGgTGgccgaggagtgggaaatggaaaatGGGCaGGgtttgcCCC
tcggGGaGCTGCACtAaAACcCCAAATGAGATGCAtGAATTCctcttcgccgacaagcagctcataaaagttaac
atcgaagtatttacagcatattttggggaaacttcaacttggaatttgattaaatgcaaactacttgaaaacata
caacttcaaatcaagtatgtacaattttaaatcaagtagatctctttgaatttattgatttgcttaaccaactta
agtttccttaacttctcttattttgtgtaTTTTGGCTAACTatTTTTGGCCAcAAatCgatctttggtcgcgcc
aaagttaatgaaccaaccgcacttttCCAATCTCGCAtttgatgcgttggattccacaTATTTGGcCAGactgaa
AAAtAATTTCaTGTTTATGGCcAcGTTGTTTTCACCgGcCACaAAAAtGCTTATCGAacgccgGAGATCgccaag
cgagagaCTTGTAAATATTTAccggTTTttgcgatttttattcgactgataaacgagtttttcccatcgcacagt
tgcgAtGTGcgaaaatgttttgtgggaagaaattgtgcacctagcccatacgattcgattcaccttctgtttgtt
ggAATAAtAAATCtGtAGAAaGGTtgttgtatTTTTagaagactccatccccctTTgTTaTTCTtAtGTAAACGA
AACCTTtttcctacacacatagctgcatagtaagttACCCTCAtGAGAACGTTTAGGccgtgaatatTGGGTAAA
TATTTAAACTGGTTgCGATTGTTTTgGCAAAATCGTTGCAGTTGGtCAAACACGTGTTTGAGATAATTtAATTAA
ATTTACTTGgCTTTTTTaaccCgTGCCACGTGCCACTcgagtccagtttgctgccctcgaaatggAGTGGCAAAC
ACTtGAGCACTTGCTttaaAAaTGcGGCAAGTGCAtttgggttgggtgggtagtttagcttggttttggctggct
ggtgaaaaTTCctagGAAtcGAAAACTTTGGTGGTCTtcggacgGCaGACAATGCAACaaTGTCatacgtactTG
GATgaatcgcTGGCATCtGTCgcagTCCGtgGAGAatAgaccgAaATTCCcCCGAActcacgagcATGAtgtggC
GATGATGACTtGtGgAAATCaaGCAatgatttccactccatCTGtAaCTAAATAccggTTATtTtCACGgtGTGt
gttagccgctctCgCCcCtCTCATTAGtcggggcaccggggTAATTTGCATAAGGTCCTGGCCCCAGGAaccAAA
ggACaCACtcgagagcgagctGGAaACTAATTAAAATGTTCGCGCcTCAAATTTCCttcgCtCGccCtTATTTAT
GaCCCTGTGAATGTCTtGCATATGGAcggagttgagatccTTCTcgttTTctTCCTTTTTTTGTTTACGAgTGAC
AAATGggACATTTACATGCgagtcagtTTGCATATtggAATTATTCATGGTTtCTttaaagatggcgcaggatgt
gatgtcgcgggaaaaggatgagggtttcctcaaggaagtcggaaatagaagtggtttcttttcatgctaccaata
tgggggcacataaaacttgcatttgatgagctaaatgtaaacatacactatttaaatacatttttatgataagt
gaacttaaagtcacactaacttttgaaaatttgattgacttctacaatgtgtgttgtttaatttcttattttata
ttttttaaatatcgaaaatctacaaatccgcttatgtttaaaagtcaagccgctggctaattgacaaaatgtgt
aattgtggcgatgagagtccttccgattgtcactctcccaaccctccgtaatcccctaagtcaccataggggttg
ggtaaaaatctaga

Green: Exd: TGAT or ATCA > CCGG Sites converted in total: 15

col-Tv D Hox-CRM

gcggccgctgatagaagtagggtatttctaacaagtacaattaaatagacatatgtactttttttcagtaaaatat
taccaattttcacgcactacttaagccaatctaccaccttaattccgtttttttcttttgaacacatgaaaaa
ATTCAATTccttaGAtTTAtcggcAtCtaATtgAaAtCCacaAATTTGtTGCCATTTgTTgtgcagAAgTATTTT
TGAATTTcGCACACGTTcgtTGgCAAAAAtatgtacaaatattgAATacGAAaAAgtcaaGGAaAAAttccattc
tTTTtctCGCaaAAATgGTGACaGAGCaAAatgctgaccgcCGCCTACTaGGAAAAATAaTCCCCAGcCGATTtg
aACGATTTATTATTTGGccCAGCCaactcggagcctgtcccggaataccCGGAACCATgccgCATcCGAGTccaG
ATCCaAGTCCgagtccgagtggtggacaggcggcattgcaattggtggacatTTtGGagtgGAAgAAAcTTTTGC
GGtACGTGGCAACGTGTTGAAATTACAAAATGTGTTAAAAAaTGaaagagcagggcgggtaaaaagcAAAATAAt
ATATTTcattCACACACAGACttttgtctctgctccgccgtccatatAAAAAACATAAATTTCCTTTGAGAATAG
AacttctgggatggagaggggggggggggggtgCAAGGTGATTATgTTTGACAGCCGACttGGGAcacgaaGcac
CGCCCccTTtttcatcGCCAaACAGGACAtGGgtattAGaTCCTGgccaaagtgccaaaactggcctcactttcc
GtCTGATTTAcggccaactatttgtttaaattttaccacttccctgctatcgacactcaacttaaaacatata
aaaccaactatttaaagagcaaataaagttcaatttgattttctttggccttcgttaataatttcctgcatctc
tctgGGGAtcaGcgaAAAATATGAAttaTTgAAACAGCTGAAggaaatAATAtaaCCAAATcggaaggccaaatc
tgtttaatgtttcagatatgttggtgtgggttgaccaaatgacaatatgctttttagCtTcAACCTCCCTCAAgc
gAAATTGATTTCAtggtttttctgtttttcgcctaaaaatACAcAAAAgagtcgAaAAAatttgactgtgatt
gtcacttgaatgtgtgggttgggaaAACCAcTCcAaATCCACttcaccaaatatgtcagtaTGGaATCACGAacc
tcGctTGATTTCTCCAGTGATTTTTCGCAATCAGccTGATGAGGATGTGCcgagaacgttgctCaTGgagGCagT
gCTATGATTAGTATCCTttcagccaggtcaaaagtgacgctgccGCATGCCAAGTGGCCtcggcCaaATGAAACT
TgccgGcgaaacaaggacaaaaggctgaaagaaggctgcctgatgaactacgtgatggtcagacaaattgaagtt
acactggaaaataattagctaatttcctagtttgcttaggttaagggattaagtaagaatgtgtttaaaggactc
ttttataggtaacttgttactctaaagaaaacttatccacagaatagatgtgctattttatcgcagtgtatgtgg
ataGtCAacGACAAAGTGaAATGcaTTGAGGttggtggcgaacaaacgcgacctgaatcgcgaattggaatagcc
agcctactccgccccctcaggatatatagctacttcgactagcatatgcgccagatagtatagctggcgtatcaa
tttcgataatgtacttggaccgcgaGtATCTTTATTaTCCTgacTGCTGtGCAATTATTtccgactgctttactg
tcgtaccgcttttccgagTCCTGGGAAAcGCAAACAAaGGatATTTCGcGCACACGcCtcggacATGATTGCATC
CGggcaGGAAGCatTgcgtCGTGTccttttttGCATCCtTACATcgtcgCTtCCTTCCtgccgatcagaacccctg
gctgcttTTCATCCGCAATTTTCACGCCCACTTTGTTTGATGATGACAAATCTCGTTGCGTGTTATCAGATTGGC
caactttccgttgctattcccctcgtATtGCAgccgTATGTTTATCTTtGGAagctgcgctcctgccacgtgtc
cttgcgttttggttagcttcctgcgattttGCTcGGAacgAGtGgCACGTGCTAATCGGCGGTCGtctgcacCGC
CCatgcaaccagccacccccccctggtaagtgcaaCCCCGCctaACAtgtCCGAgccgTGAAATCGCTAAGCtgaa
caacggaatcgaaatggcttcattaatcaggtcacttcgatggcagcgcataatgttcttcaaaaatttgaattg
ggggcggttgaaaagaggaaattggtctatatagcacttagcgcgattgaatttgctcattttgtgttacctaat
tggggaaaagatgtttcggttattaatcatgagtgatacttactattgtattacaaaaatggtgcatatatcaga
agatatatattattaatagatcttaaggagcatttgtatccattcaccaaaatatggtaaaaataaagcttaaat
taaatttgctcatttacctTTAAggacatcctttgctgccgGAGTGCaCTCAtgcccacacccactcgcaacCGA
ATGAGTGTCGCCCTGcGGACGCATAAcggcTaAATAAAAgCCtCaCACACGTGGAGAaagagaggtgtatataac
cttagagagagagagagagagggagagctaCAGTCTGAGGCCaAGaAGTGTgCTAAATATTTAAgtACTtAAAACCA
TAAACCTGAGTATTTATAAATTggctttaacaagctcgtatgtgacggtggttgggtgtgtgtctgtgggCtGAC
CCTTTTttcGGgcacaaggatctgctcgactcgattccatTCATtAGAAATGCtcACTAATGTGCCCGaCTgccg
AATAATCAAATTTATATGCATGGTTATGTCCTTtAgagcaggaccacaaaactgaagtttcgagtggattgcgaa
tgggattggctgttaagtttacggagaaacctttgagaatgagacgaagataataTGTCAATATCCTTGttttcc
aaaatgtctgaccaaaagtgcagatgtccagtatcgaaacacgcccaagaccggtaccgttattctgctaccagt
tttcttgtttgtttttgtttgttagccagcttgtttgtggccgacactgagaccaaaaaggccaaaaaggtcgcc
ccctcgggatttccttagtcctgctgccagcttcttccttccacattcttttgggggtccGATGAGatTTGaAA
aTttcACAAAAcggcATTAggaatgctggcatccatgttgtgataggctagttcttttctagcctagcaacattt
ttgatgtgactaaagagaatcaTCTTTaTGGCCAcacccggaatactcattcttcaaacaacaagcgacaataaa
aacaaattacaattgcaacaatggcagcggcagcaaaacatttgagacattttccccacacacacacacagacac
acacatacttcagttaaatgaacatttgcaggacctagatatttatttattacaggggttgcactgcaggagcat
taacccccttactgcccacggcagtctcgagcaacggaggactgaAAAAATTGAAATAAcggcCCAAAACTAAAGG
CAACAACTGTTCGCAgccgGAAGTCAAATTATATTTgTgCCTaagtgcactgagagaaatttaatataacctatg
atttacacgatcgatttcgatctttgcaacatttttgagtgaaactaggaaaccctttacaagcgagatcttg
cagttctttagtttctaagcattattctagtattttaaaatatatttccttgttctggtcttaatttattata
actaatgaatattttccgtctgtgtattcggaaaaggggagttggaacatataaagatggggtgcagaaaagat
gatcagggaaggtgtgagtggttcaaactagctgggcgtttggatagcgaAGGGTGcGTCCtttgcTGGTCCTTc
gAATTATGCAAATcgtcTGCACttcagatactactacccctacaaaaaaaaaaaacgaagagaaaacccccctaca
aacaaacgtATTAGtGTCAtCgaGCATTtGCCaagCCATTtCaTtGACACgTagaacacctttttcgtccacttc
gtatGATGTTATCATTgccgTCtTGggGAcggcACACGGACATTGtCAaCcGTAAATGAGCTAATgccgCTAgTA

AATGTgTCGcagctcattccacaatttctctactacttgagagcgaggagtgggttcGCGTTTTTGTGGTTCTCC
AGAGGATCCCCAatGcTGACACGCTTCTCCAGgTGgccgaggagtgggaaatggaaaatGGGCaGGgtttgcCCC
tcggGGaGCTGCACtAaAACcCCAAATGAGATGCAtGAATTCctcttcgccgacaagcagctcataaaagttaac
atcgaagtatttacagcatatttggggaaacttcaacttggaatttgattaaatgcaaactacttgaaaacata
caacttcaaatcaagtatgtacaattttaaatcaagtagatctctttgaatttattgatttgcttaaccaactta
agtttccttaacttctcttatttttgtgtaTTTTGGCTAACTatTTTTGGCCAcAAatCgatctttggtcgcgcc
aaagttaatgaaccaaccgcacttttCCAATCTCGCAtttgatgcgttggattccacaTATTTGGcCAGactgaa
AAAtAATTTCaTGTTTATGGCcAcGTTGTTTTCACCgGcCACaAAAAtGCTTATCGAacgccgGAGATCgccaag
cgagagaCTTGTAAATATTTATGATTTTttgcgattttattcgactgataaacgagtttttcccatcgcacagt
tgcgAtGTGcgaaaatgttttgtgggaagaaattgtgcacctagcccatacgattcgattcaccttctgtttgtt
ggAATAAtAAATCtGtAGAAaGGTtgttgtatTTTTagaagactccatccccctTTgTTaTTCTtAtGTAAACGA
AACCTTtttcctacacacatagctgcatagtaagttACCCTCAtGAGAACGTTTAGGccgtgaatatTGGGTAAA
TATTTAAACTGGTTgCGATTGTTTTgGCAAAATCGTTGCAGTTGGtCAAACACGTGTTTGAGAgccgTtAcggcA
ATTTACTTGgCTTTTTTaaccCgTGCCACGTGCCACTcgagtccagtttgctgccctcgaaatggAGTGGCAAAC
ACTtGAGCACTTGCTttaaAAaTGcGGCAAGTGCAtttgggttgggtgggtagtttagcttggttttggctggct
ggtgaaaaTTCctagGAAtcGAAAACTTTGGTGGTCTtcggacgGCaGACAATGCAACaaTGTCatacgtactTG
GATgaatcgcTGGCATCtGTCgcagTCCGtgGAGAatAgaccgAaATTCCcCCGAActcacgagcATGAtgtggC
GATGATGACTtGtGgAAATCaaGCAatgatttccactccatCTGtAaCTAAATATGATTTATtTtCACGgtGTGt
gttagccgctctCgCCcCtCTCATTAGtcggggcaccggggTAATTTGCATAAGGTCCTGGCCCCAGGAaccAAA
ggACaCACtcgagagcgagctGGAaACTAcggcAAATGTTCGCGCcTCAAATTTCCttcgCtCGccCtTATTTAT
GaCCCTGTGAATGTCTtGCATATGGAcggagttgagatccTTCTcgttTTctTCCTTTTTTTGTTTACGAgTGAC
AAATGggACATTTACATGCgagtcagtTTGCATATtggAcggcTTCATGGTTtCTttaaagatggcgcaggatgt
gatgtcgcgggaaaaggatgagggtttcctcaaggaagtcggaaatagaagtggtttcttttcatgctaccaata
tgggggcacataaaacttgcatttgatgagctaaatgtaaacatacactatttaaatacatttttttatgataagt
gaacttaaagtcacactaactttttgaaaatttgattgacttctacaatgtgtgttgtttaatttcttattttata
ttttttaaatatcgaaaatctacaaatccgcttatgtttaaaagtcaagccgctggctaattgacaaaatgtgt
aattgtggcgatgagagtccttccgattgtcactctcccaaccctccgtaatcccctaagtcaccatagggggttg
ggtaaaaatctaga

Red: HOX: ATTA > CGGC, TAAT > GCCG
Sites converted in total: 18

col-Tv D Hth-CRM

gcggccgctgatagaagtagggtatttctaacaagtacaattaaatagacatatgtactttttttcagtaaaatat
taccaattttcacgcactacttaagccaatctaccaccttaattccgtttttttttcttttgaacacatgaaaaa
ATTCAATTccttaGAtTTAtATTAAtCtaATtgAaAtCCacaAATTTGtTGCCATTTgTTgtgcagAAgTATTTT
TGAATTTcGCACACGTTcgtTGgCAAAAAtatgtacaaatattgAATacGAAaAAgtcaaGGAaAAAttccattc
tTTTtctCGCaaAAATgGTGACaGAGCaAAatgctgaccgcCGCCTACTaGGAAAAATAaTCCCCAGcCGATTtg
aACGATTTATTATTTGGccCAGCCaactcggagcctgtcccggaataccCGGAACCATTAATCATcCGAGTccaG
ATCCaAGTCCgagtccgagtggtggacaggcggcattgcaattggtggacatTTtGGagtgGAAgAAAcTTTTGC
GGtACGgataAACGTGTTGAAATTACAAAATGTGTTAAAAAaTGaaagagcagggcgggtaaaaagcAAAATAAt
ATATTTcattCACACACAGACttttgtctctgctccgccgtccatatAAAAAACATAAATTTCCTTTGAGAATAG
AacttctgggatggagaggggggggggggggtgCAAGGTGATTATgTTcaggAGCCGACttGGGAcacgaaGcac
CGCCCccTTtttcatcGCCAaACAGGACAtGGgtattAGaTCCTGgccaaagtgccaaaactggcctcactttcc
GtCTGATTTAcggccaactatttgtttaaattttaccacttccctgctatcgacactcaacttaaaacatata
aaaccaactatttaaagagcaaataaagttcaatttgattttctttggccttcgttaataattttcctgcatctc
tctgGGGAtcaGcgaAAAATATGAAttaTTgAAACAGCTGAAggaaatAATAtaaCCAAATcggaaggccaaatc
tgtttaatgtttcagatatgttggtgtgggttgaccaaatgacaatatgctttttagCtTcAACCTCCCTCAAgc
gAAATTGATTTTCAtggtttttctgtttttcgcctaaaaatACAcAAAAgagtcgAaAAAatttgactgtgatt
gtcacttgaatgtgtgggttgggaaAACCAcTCcAaATCCACttcaccaaatatgtcagtaTGGaATCACGAacc
tcGctTGATTTCTCCAGTGATTTTTCGCAATCAGccTGATGAGGATGTGCcgagaacgttgctCaTGgagGCagT
gCTATGATTAGTATCCTttcagccaggtcaaaagtgacgctgccGCATGCCAAGTGGCCtcggcCaaATGAAACT
TTAATGcgaaacaaggacaaaaggctgaaagaaggctgcctgatgaactacgtgatggtcagacaaattgaagtt
acactggaaaataattagctaatttcctagtttgcttaggttaagggattaagtaagaatgtgtttaaaggactc
ttttataggtaacttgttactctaaagaaaacttatccacagaatagatgtgctattttatcgcagtgtatgtgg
ataGtCAacGACAAAGTGaAATGcaTTGAGGttggtggcgaacaaacgcgacctgaatcgcgaattggaatagcc
agcctactccgccccctcaggatatatagctacttcgactagcatatgcgccagatagtatagctggcgtatcaa
tttcgataatgtacttggaccgcgaGtATCTTTATTaTCCTgacTGCTGtGCAATTATTtccgactgctttactg
tcgtaccgcttttccgagTCCTGGGAAAcGCAAACAAaGGatATTTCGcGCACACGcCtcggacATGATTGCATC
CGggcaGGAAGCatTgcgtCGTGTcctttttGCATCCtTACATcgtcgCTtCCTTCCtgccgatcagaacccctg
gctgcttTTCATCCGCAATTTTCACGCCCACTTTGTTTGATGAcaggAAATCTCGTTGCGTGTTATCAGATTGGC

caactttccgttgctattccccctcgtATtGCATAATTATGTTTATCTTtGGAagctgcgctcctgccacgtgtc
cttgcgttttggttagcttcctgcgattttGCTcGGAacgAGtGgCACGTGCTAATCGGCGGTCGtctgcacCGC
CCatgcaaccagccaccccccctggtaagtgcaaCCCCGCctaACAtgtCCGATAATTGAAATCGCTAAGCtgaa
caacggaatcgaaatggcttcattaatcaggtcacttcgatggcagcgcataatgttcttcaaaaatttgaattg
gggcggttgaaaagaggaaattggtctatatagcacttagcgcgattgaatttgctcattttgtgttacctaat
tggggaaaagatgtttcggttattaatcatgagtgatacttactattgtattacaaaaatggtgcatatatcaga
agatatatattattaatagatcttaaggagcatttgtatccattcaccaaaatatggtaaaaataaagcttaaat
taaatttgctcatttacctTTAAggacatcctttgctTAATGAGTGCaCTCAtgcccacacccactcgcaacCGA
ATGAGTGTCGCCCTGcGGACGCATAAATTATaAATAAAAgCCtCaCACACGTGGAGAaagagaggtgtatataac
cttagagagagagagagagggagagctaCAGTCTGAGGCCaAGaAGTGTgCTAAATATTTAAgtACTtAAAACCA
TAAACCTGAGTATTTATAAATTggctttaacaagctcgtatgtgacggtggttgggtgtgtgtctgtgggCtGAC
CCTTTTttcGGgcacaaggatctgctcgactcgattccatTCATtAGAAATGCtcACTAATGTGCCCGaCTTAAT
AATAATCAAATTTATATGCATGGTTATGTCCTTtAgagcaggaccacaaaactgaagtttcgagtggattgcgaa
tgggattggctgttaagtttacggagaaacctttgagaatgagacgaagataataTccggATATCCTTGttttcc
aaaatgtctgaccaaaagtgcagatgtccagtatcgaaacacgcccaagaccggtaccgttattctgctaccagt
tttcttgtttgtttttgtttgttagccagcttgtttgtggccgacactgagaccaaaaaggccaaaaaggtcgcc
ccctcgggatttccttagtcctgctgccagcttcttccttccacattctttttgggggtccGATGAGatTTGaAA
aTttcACAAAAATTAATTAggaatgctggcatccatgttgtgataggctagttcttttctagcctagcaacattt
ttgatgtgactaaagagaatcaTCTTTaTGGCCAcacccggaatactcattcttcaaacaacaagcgacaataaa
aacaaattacaattgcaacaatggcagcggcagcaaaacatttgagacattttccccacacacacacacagacac
acacatacttcagttaaatgaacatttgcaggacctagatatttatttattacaggggttgcactgcaggagcat
taaccccttactgcccacggcagtctcgagcaacggaggactgaAAAAATTGAAATAAATTACCAAAACTAAAGG
CAACAACTGTTCGCATAATGAAGTCAAATTATATTTgTgCCTaagtgcactgagagaaatttaatataacctatg
atttacacgatcgatttcgatctttgcaacatttttgagtgaaactaggaaaccctttacaagcgagatcttg
cagttctttagtttctaagcattattctagtattttaaaatatattttcctttgttctggtcttaatttattata
actaatgaatattttccgtctgtgtattcggaaaaggggagttggaacatataaagatggggtgcagaaaagat
gatcagggaaggtgtgagtggttcaaactagctgggcgtttggatagcgaAGGGTGcGTCCtttgcTGGTCCTTc
gAATTATGCAAATcgtcTGCACttcagatactactacccctacaaaaaaaaaaaacgaagagaaaacccccctaca
aacaaacgtATTAGtGTCAtCgaGCATTtGCCaagCCATTtCaTtGACACgTagaacaccttttcgtccacttc
gtatGATGTTATCATTTAATTCtTGggGAATTAACACGGACATTGtCAaCcGTAAATGAGCTAATTAATCTAgTA
AATGTgTCGcagctcattccacaatttctctactacttgagagcgaggagtgggttcGCGTTTTTGTGGTTCTCC
AGAGGATCCCCAatGccaggACGCTTCTCCAGgTGgccgaggagtgggaaatggaaaatGGGCaGGgtttgcCCC
tcggGGaGCTGCACtAaAACcCCAAATGAGATGCAtGAATTCctcttcgccgacaagcagctcataaaagttaac
atcgaagtatttacagcatattttggggaaacttcaacttggaatttgattaaatgcaaactacttgaaaacata
caacttcaaatcaagtatgtacaattttaaatcaagtagatctctttgaatttattgatttgcttaaccaactta
agtttccttaacttctcttatttttgtgtaTTTTGGCTAACTatTTTTGGCCAcAAatCgatctttggtcgcgcc
aaagttaatgaaccaaccgcacttttCCAATCTCGCAtttgatgcgttggattccacaTATTTGGcCAGactgaa
AAAtAATTTCaTGTTTATGGCcAcGTTGTTTTCACCgGcCACaAAAAtGCTTATCGAacgccgGAGATCgccaag
cgagagaCTTGTAAATATTTATGATTTTttgcgatttttattcgactgataaacgagtttttcccatcgcacagt
tgcgAtGTGcgaaaatgttttgtgggaagaaattgtgcacctagcccatacgattcgattcaccttctgtttgtt
ggAATAAtAAATCtGtAGAAaGGTtgttgtatTTTTagaagactccatccccctTTgTTaTTCTtAtGTAAACGA
AACCTTtttcctacacacatagctgcatagtaagttACCCTCAtGAGAACGTTTAGGccgtgaatatTGGGTAAA
TATTTAAACTGGTTgCGATTGTTTTgGCAAAATCGTTGCAGTTGGtCAAACACGTGTTTGAGATAATTtAATTAA
ATTTACTTGgCTTTTTTaaccCgTGCCACGTGCCACTcgagtccagtttgctgccctcgaaatggAGgataAAAC
ACTtGAGCACTTGCTttaaAAaTGcGGCAAGTGCAtttgggttgggtgggtagtttagcttggttttggctggct
ggtgaaaaTTCctagGAAtcGAAAACTTTGGTGGTCTtcggacgGCaGACAATGCAACaaTGTCatacgtactTG
GATgaatcgcTGGCATCtGTCgcagTCCGtgGAGAatAgaccgAaATTCCcCCGAActcacgagcATGAtgtggC
GATGATGACTtGtGgAAATCaaGCAatgatttccactccatCTGtAaCTAAATATGATTTATtTtCACGgtGTGt
gttagccgctctCgCCcCtCTCATTAGtcggggcaccggggTAATTTGCATAAGGTCCTGGCCCCAGGAaccAAA
ggACaCACtcgagagcgagctGGAaACTAATTAAAATGTTCGCGCcTCAAATTTCCttcgCtCGccCtTATTTAT
GaCCCTGTGAATGTCTtGCATATGGAcggagttgagatccTTCTcgttTTctTCCTTTTTTTGTTTACGAgcagg
AAATGggACATTTACATGCgagtcagtTTGCATATtggAATTATTCATGGTTtCTttaaagatggcgcaggatgt
gatgtcgcgggaaaaggatgagggtttcctcaaggaagtcggaaatagaagtggtttcttttcatgctaccaata
tgggggcacataaaacttgcatttgatgagctaaatgtaaacatacactatttaaatacatttttatgataagt
gaacttaaagtcacactaacttttgaaaatttgattgacttctacaatgtgtgttgtttaatttcttattttata
ttttttaaatatcgaaaatctacaaatccgcttatgtttaaaagtcaagccgctggctaattgacaaaatgtgt
aattgtggcgatgagagtccttccgattgtcactctcccaaccctccgtaatcccctaagtcaccataggggttg
ggtaaaaatctaga

Purple: Hth: TGGC -> GATA; TGAC -> CAGG; GTCA -> CCGG
Sites converted in total: 7

apS2-CRM fragment

CCCGctgccaAAACAACCCTaAGGCGATTATCTCAAGTGCTcGCCcttccaggactatGtTTGCCTccgtgcacc
ctgCTGtAGATGCTTATCGATTACACGGtGaGCGATGAAATCATTACTCCGAATTAAATAAACGTCATACTCGCT
TtTCGTCAActtGtaTTTGAtgTCTCAATgagcgagcgtgccattggcaagggaaccCATTTGCATcgcaGATTT
agaccctggcactgtgatagtgtgtaaacacgtcgcAATTAGTTGaCctaAaCGAaTgCCCCggcgtaccgtcgt
cttccggtcagagtcccggaATGAcATCAagCTCCGAttagTcACTTTCTAGTtGgTTGGGAGcCaAGCAGCTGc
CtgccatCCgCaACTCtCGCTcttattgtttgtccccatATcTGTATTTGTcGcaCaACTTTGTTGTGATTACGG
CGATAAAGCGgcTCaAGTAAATCACAATAAtacgacggtctaGTCtGgCCGActcGgTtCgAAAGAAAGTGTGAA
TAgagccgggctcaacaatcaacaacaatggattcgccggaccggaccagcaatgacaaatgtctctaggcttat
gagtgggCATGtgCATAAAAGTcATAAGCaATAGAAAtAATAATtGTGttCaCGTCATGGGaCACGTCAacaaaa
actgccgAGaAgCAATTGcgcGACACTTTCTTGgGTgTAAATTAAGaTACTTTaTAGCTGGGgtggtgccgtggt
cgCTTCCGcTgGCTACTGGTTTtgaacccagGGCCacgAAACCAGTTCCAGCTcAggcccttggtgaagtccaag
ctggtggccagcttgggtttttgtgtatcactcgattgtaTTTtGcTCTTTGCATGCGTCAGCGatatatatata
ttgaaccgctagcatattcattgattttcgaacagtaattctgcaatATCAtTGcctgtTCAAcTTCGTGGGACt
AGCACAacccatctcttgcctccccgcaccgtcccgcgaagaaatgccgtcacgtacgcgagccccatcgttcaa
caaataaagaatgaaccggatcCTCGgAAAggcgtggCATCGTAATGAtAcTGATGCGTTAGTaAAATTGCggtC
AAATTATAGGAGgccagctctaaAATCGAgcgACCTtttgaaggtaGGCAGGAAATGCGAAATTCGGTAGCCtGT
TTATTAAATACgAAAaTATatgaatccattgcgggcTgGCAGGACctaagttgtggggcatttgtagccgagatc
agaacGGtCaGCTACCTGtGGATGGGTCCCgAaATCACAcGAAATAGtCtaAGCtATAAATTAGATttTTATCtc
gttgccgcaggaataaaaggaggcaagcctcctactgacgctcgatctctggagagcTGTCgCATaTtGGGGCGA
TCCAATTAAGTGGTAATTTTTAtCATTTTaGCGTCGagGGCCGggttagttctgttgggtcgcttttactccgaa
atATTTGCTGACAAACGGAAGCGATATgTTGAaATTaTTCaGCGTAAggAtTCCaTGaaccTTCggcagagcCTG
GGAGTGaGAttcagagctggaatttgaatcgattcgagggggctacacccgcttcgatgaaacgatttttttgtcg
ggccttgcttgattgactttactcaagattgacttcatgaaggcaatttagaattgggctgataatcagtccggt
acataagtcgcTtTaTTTGCcTTGATTATATGCAcagtgtgcattgcacACTACGgAaGCtgcactatgactgag
ggggtcataAAGCGTAGGTGAGagggTcTTGTTCgcggggtaacttgatTTGTTTGTCTaTTTgTTCGcctccga
ctctttgctttcgactgcctttacagggaggacaagtattaatgtaaaatggtaactggtcatctgaggcgtcat
ttgggtctggctcggggt

apSJ2 WT fragment

GGATCCCTAGGTTAGTAGAATTCGCCCTTatgtgttttggttccaggtccgttgggaacccagcttcaaatgaag
ttatcacagcgattatattccttaaaacgttcagaaacctccgcccgtgcaatttgaacagctttctggggtggg
agtttaattttgagggtttgtgtataaaaggccaAACAACCCTAATTGAAATTATGCGGTGTGgCTCtGCAGCGA
cTGCTCGTTTaGGCtgTGGTATTCCGAAGGGAGTACTaaAGGATTTCatCGGGCgGgttGCGAACCTTTTGCGGc
GGAACTGATTAGCAGACAATATGTCAAAGCTtgccaCtGGGAGGGTGaActCTGtAGCTTCACCCtTcaagcggC
gtTGcGACtactccAGGGACagCtacTCGAGGcTCGGctGATAAGGGaAAAAGTCATGACAAATAAACGGATACA
TGTGTCGGCAATAGTTTTGCTtATTGCCGCGATAATAATCGACgtGaACAACAATGGtCTTTGTCCtGGTcaTGC
TCCTGCTGCcTGGGTGCCCAcaTaAAGaACAaTCGCGAGCGCGCGCCCAACgAgAATTACAGGATCGTTTatggc
aaccgcctggtagCAGTCTCCTGGTAGCACCCGctgccaAAACAACCCTaAGGCGATTATCTCAAGTGCTcGCCc
ttccaggactatGtTTGCCTccgtgcaccctgCTGtAGATGCTTATCGATTACACGGtGaGCGATGAAATCATTA
CTCCGAATTAAATAAACGTCATACTCGCTTtTCGTCAActtGtaTTTGAtgTCTCAATgagcgagcgtgccattg
gcaagggaaccCATTTGCATcgcaGATTTagaccctggcactgtgatagtgtgtaaacacgtcgcAATTAGTTGa
CctaAaCGAaTgCCCCggcgtaccgtcgtcttccggtcagagtcccggaATGAcATCAagCTCCGAttagTcACT
TTCTAGTtGgTTGGGAGcCaAGCAGCTGcCtgccatCCgCaACTCtCGCTcttattgtttgtccccatATcTGTA
TTTGTcGcaCaACTTTGTTGTGATTACGGCGATAAAGCGgcTCaAGTAAATCACAATAAtacgacggtctaGTCt
GgCCGActcGgTtCgAAAGAAAGTGTGAATAgagccgggctcaacaatcaacaacaatggattcgccggaccgga
ccagcaatgacaaatgtctctaggcttatgagtgggAAGGGCGAATTCTAACCTATTCTAGA

apJ2-D-Col

GGATCCCTAGGTTAGTAGAATTCGCCCTTatgtgttttggttccaggtccgttgggaacccagcttcaaatgaag
ttatcacagcgattatattccttaaaacgttcagaaacctccgcccgtgcaatttgaacagctttctggggtggg
agtttaattttgagggtttgtgtataaaaggccaAACAACCCTAATTGAAATTATGCGGTGTGgCTCtGCAGCGA
cTGCTCGTTTaGGCtgTGGTGCCTTACCTATCAGACTaaAGGATTTCatCGGGCgGgttGCGAACCTTTTGCGGc
GGAACTGATTAGCAGACAATATGTCAAAGCTtgccaCtGGGAGGGTGaActCTGtAGCTTCACCCtTcaagcggC
gtTGcGACtactccAGGGACagCtacTCGAGGcTCGGctGATAAGGGaAAAAGTCATGACAAATAAACGGATACA
TGTGTCGGCAATAGTTTTGCTtATTGCCGCGATAATAATCGACgtGaACAACAATGGtCTTTGTCCtGGTcaTGC
TCCTGCTGCcTGGGTGCCCAcaTaAAGaACAaTCGCGAGCGCGCGCCCAACgAgAATTACAGGATCGTTTatggc
aaccgcctggtagCAGTCTCCTGGTAGCACCCGctgccaAAACAACCCTaAGGCGATTATCTCAAGTGCTcGCCc
ttccaggactatGtTTGCCTccgtgcaccctgCTGtAGATGCTTATCGATTACACGGtGaGCGATGAAATCATTA

CTCCGAATTAAATAAACGTCATACTCGCTTtTCGTCAActtGtaTTTGAtgTCTCAATgagcgagcgtgccattg
gcaagggaaccCATTTGCATcgcaGATTTagaccctggcactgtgatagtgtgtaaacacgtcgcAATTAGTTGa
CctaAaCGAaTgCCCCggcgtaccgtcgtcttccggtcagagtcccggaATGAcATCAagCTCCGAttagTcACT
TTCTAGTtGgTTGGGAGcCaAGCAGCTGcCtgccatCCgCaACTCtCGCTcttattgtttgtccccatATcTGTA
TTTGTcGcaCaACTTTGTTGTGATTACGGCGATAAAGCGgcTCaAGTAAATCACAATAAtacgacggtctaGTCt
GgCCGActcGgTtCgAAAGAAAGTGTGAATAgagccgggctcaacaatcaacaacaatggattcgccggaccgga
ccagcaatgacaaatgtctctaggcttatgagtgggAAGGGCGAATTcTAACCTATTCTAGA

apJ2-D-Antp

GGATCCCTAGGTTAGTAGAATTCGCCCTTatgtgttttggttccaggtccgttgggaacccagcttcaaatgaag
ttatcacagcgattatattccttaaaacgttcagaaacctccgcccgtgcaatttgaacagctttctggggtggg
agtttaattttgagggtttgtgtataaaaggccaAACAACCCgccgTGAAcggcTGCGGTGTGgCTCtGCAGCGA
cTGCTCGTTTaGGCtgTGGTATTCCGAAGGGAGTACTaaAGGATTTCatCGGGCgGgttGCGAACCTTTTGCGGc
GGAACTGATTAGCAGACAATATGTCAAAGCTtgccaCtGGGAGGGTGaActCTGtAGCTTCACCCtTcaagcggC
gtTGcGACtactccAGGGACagCtacTCGAGGcTCGGctGATAAGGGaAAAAGTCATGACAAATAAACGGATACA
TGTGTCGGCAATAGTTTTGCTtATTGCCGCGATAATAATCGACgtGaACAACAATGGtCTTTGTCCtGGTcaTGC
TCCTGCTGCcTGGGTGCCCAcaTaAAGaACAaTCGCGAGCGCGCGCCCAACgAgAcggcCAGGATCGTTTatggc
aaccgcctggtagCAGTCTCCTGGTAGCACCCGctgccaAAACAACCCTaAGGCGATTATCTCAAGTGCTcGCCc
ttccaggactatGtTTGCCTccgtgcaccctgCTGtAGATGCTTATCGATTACACGGtGaGCGATGAAATCATTA
CTCCGAcggcAATAAACGTCATACTCGCTTtTCGTCAActtGtaTTTGAtgTCTCAATgagcgagcgtgccattg
gcaagggaaccCATTTGCATcgcaGATTTagaccctggcactgtgatagtgtgtaaacacgtcgcAcggcGTTGa
CctaAaCGAaTgCCCCggcgtaccgtcgtcttccggtcagagtcccggaATGAcATCAagCTCCGAttagTcACT
TTCTAGTtGgTTGGGAGcCaAGCAGCTGcCtgccatCCgCaACTCtCGCTcttattgtttgtccccatATcTGTA
TTTGTcGcaCaACTTTGTTGTGATTACGGCGATAAAGCGgcTCaAGTAAATCACAATAAtacgacggtctaGTCt
GgCCGActcGgTtCgAAAGAAAGTGTGAATAgagccgggctcaacaatcaacaacaatggattcgccggaccgga
ccagcaatgacaaatgtctctaggcttatgagtgggAAGGGCGAATTcTAACCTATTCTAGA

apJ2-D-Exd

GGATCCCTAGGTTAGTAGAATTCGCCCTTatgtgttttggttccaggtccgttgggaacccagcttcaaatgaag
ttatcacagcgattatattccttaaaacgttcagaaacctccgcccgtgcaatttgaacagctttctggggtggg
agtttaattttgagggtttgtgtataaaaggccaAACAACCCTAATTGAAATTATGCGGTGTGgCTCtGCAGCGA
cTGCTCGTTTaGGCtgTGGTATTCCGAAGGGAGTACTaaAGGATTTCatCGGGCgGgttGCGAACCTTTTGCGGc
GGAACccggTAGCAGACAATATGTCAAAGCTtgccaCtGGGAGGGTGaActCTGtAGCTTCACCCtTcaagcggC
gtTGcGACtactccAGGGACagCtacTCGAGGcTCGGctGATAAGGGaAAAAGTCATGACAAATAAACGGATACA
TGTGTCGGCAATAGTTTTGCTtATTGCCGCGATAATAATCGACgtGaACAACAATGGtCTTTGTCCtGGTcaTGC
TCCTGCTGCcTGGGTGCCCAcaTaAAGaACAaTCGCGAGCGCGCGCCCAACgAgAATTACAGGATCGTTTatggc
aaccgcctggtagCAGTCTCCTGGTAGCACCCGctgccaAAACAACCCTaAGGCGATTATCTCAAGTGCTcGCCc
ttccaggactatGtTTGCCTccgtgcaccctgCTGtAGATGCTTATCGATTACACGGtGaGCGATGAAccggTTA
CTCCGAATTAAATAAACGTCATACTCGCTTtTCGTCAActtGtaTTTGAtgTCTCAATgagcgagcgtgccattg
gcaagggaaccCATTTGCATcgcaGATTTagaccctggcactgtgatagtgtgtaaacacgtcgcAATTAGTTGa
CctaAaCGAaTgCCCCggcgtaccgtcgtcttccggtcagagtcccggaATGAcATCAagCTCCGAttagTcACT
TTCTAGTtGgTTGGGAGcCaAGCAGCTGcCtgccatCCgCaACTCtCGCTcttattgtttgtccccatATcTGTA
TTTGTcGcaCaACTTTGTTGccggTACGGCGATAAAGCGgcTCaAGTAAccggCAATAAtacgacggtctaGTCt
GgCCGActcGgTtCgAAAGAAAGTGTGAATAgagccgggctcaacaatcaacaacaatggattcgccggaccgga
ccagcaatgacaaatgtctctaggcttatgagtgggAAGGGCGAATTcTAACCTATTCTAGA

eya-CRM WT

CTCGAggatcacagaatccagcttgctgggtatgctgtagtcgacgcccttcgcacGtTCCGcCAATTTggctgc
gttgtttcttgggTTAAcCGTTgCCAgGCCTATGAAATggTGcttgcaaccactcgccccgtccggaaaaCaACA
ACattgAGGCATCGCTGACAAATTGAATTTGTAAACTTGtCCgcgggCAACAACAAATCATtGGAGACCCCCaGG
GATGCTGtttcggttgctgatccgtccgttgccataaccatccggattccggagtcagcaggaggAggaggagga
GGAGGAcgacgccgcccgacacggaggtggatgcgaatgccatgggacgcTGGTAATCCaaTACCCAtgCCCTaT
CcATGTTACGCTCCTCTGGACGGACTTTCTAACTCGGTcTCagttcgccggctcgGTTCTgtcctcccaaccagc
CgCCCAtcactCCCCCAcaaaaacagtggCGAAAGTGATGTGAAATTaTAGActtgctttcgggcggttgGGGGC
GTGTCaAcgCAGTTGGaAAAAAaAaCAcacacacacgcttacgCACAATTATTAACACTTATTAATGTTGAGGTT
TccataTAtATGGCACCCCAGGGCTTAAATTAAAGTCATCCCgAGTGCCAAACAGCTGcCTCAAGTGccGCACTT
GAGccgatctggatcccggaacAGGGGGTTGCaacccggggggttgagtggggttgggcgttGGCTTTGTCGcTGT
TTAAGTAGTCATTAAGATcGAGTGtGGATAAGATTTTTTCACAGCTGGCtgTCCtgccgttggtaccagccagta
cattcgccaacccctcgccaaacgaaaaaaaaaaacaagaacaagaaccccgcCTtGCAATTAGCcaacAACCTT
TGACTccggcccaactcccttcatggtgagccggaatcctgactgtccggaataagCTATTGACAGCTATTGTCt
tCTAATTAAACAACAATGTTGACCTATTCtCAAgAtGTCACGGaTCgGGTTCAatGaAAAcTTcGTTTTgGgtta

AAAAATCaTTGCaAaTTAtttacactactttTTcAGaAAATCGgtTgCaTTGTTTGTTAATTAAAAaCTTTtgat
tgctaggcgaagaacaacccttaaagaagttcgccttgctaaccctttgagtttatctgctgaagaagttgcttg
ttaaccctttgagttgcgcttcaacccttttatccttgtaataattaagttgaaggaaagacagtattgtaaatt
ataaagttgagttgagattgggcgagTCTAGA

eya-D Exd CRM

CTCGAggCCGGcagaatccagcttgctgggtatgctgtagtcgacgcccttcgcacGtTCCGcCAATTTggctgc
gttgtttcttgggTTAAcCGTTgCCAgGCCTATGAAATggTGcttgcaaccactcgccccgtccggaaaaCaACA
ACattgAGGCATCGCTGACAAATTGAATTTGTAAACTTGtCCgcgggCAACAACAACCGGTtGGAGACCCCCaGG
GATGCTGtttcggttgcCCGGccgtccgttgccataaccatccggattccggagtcagcaggaggAggaggagga
GGAGGAcgacgccgcccgacacggaggtggatgcgaatgccatgggacgcTGGTAATCCaaTACCCAtgCCCTaT
CcATGTTACGCTCCTCTGGACGGACTTTCTAACTCGGTcTCagttcgccggctcgGTTCTgtcctcccaaccagc
CgCCCCCGGctCCCCCAcaaaaacagtggCGAAAGCCGGGTGAAATTaTAGActtgctttcgggcggttgGGGGC
GTGTCaAcgCAGTTGGaAAAAAaAaCAcacacacacgcttacgCACAATTATTAACACTTATTAATGTTGAGGTT
TccataTAtATGGCACCCCAGGGCTTAAATTAAAGTCATCCCgAGTGCCAAACAGCTGcCTCAAGTGccGCACTT
GAGccgatctggatcccggaacAGGGGGTTGCaacccgggggttgagtggggttgggcgttGGCTTTGTCGcTGT
TTAAGTAGTCATTAAGATcGAGTGtGGATAAGATTTTTTCACAGCTGGCtgTCCtgccgttggtaccagccagta
cattcgccaacccctcgccaaacgaaaaaaaaaaacaagaacaagaaccccgcCTtGCAATTAGCcaacAACCTT
TGACTccggcccaactcccttcatggtgagccggaatcctgactgtccggaataagCTATTGACAGCTATTGTCt
tCTAATTAAACAACAATGTTGACCTATTCtCAAgAtGTCACGGaTCgGGTTCAatGaAAAcTTcGTTTTgGgtta
AAAACCGGTTGCaAaTTAtttacactactttTTcAGaAAATCGgtTgCaTTGTTTGTTAATTAAAAaCTTTCCGG
tgctaggcgaagaacaacccttaaagaagttcgccttgctaaccctttgagtttatctgctgaagaagttgcttg
ttaaccctttgagttgcgcttcaacccttttatccttgtaataattaagttgaaggaaagacagtattgtaaatt
ataaagttgagttgagattgggcgagTCTAGA

TGAT or ATCA > CCGG

eya-D Hox CRM

CTCGAggatcacagaatccagcttgctgggtatgctgtagtcgacgcccttcgcacGtTCCGcCAATTTggctgc
gttgtttcttgggTTAAcCGTTgCCAgGCCTATGAAATggTGcttgcaaccactcgccccgtccggaaaaCaACA
ACattgAGGCATCGCTGACAAATTGAATTTGTAAACTTGtCCgcgggCAACAACAAATCATtGGAGACCCCCaGG
GATGCTGtttcggttgctgatccgtccgttgccataaccatccggattccggagtcagcaggaggAggaggagga
GGAGGAcgacgccgcccgacacggaggtggatgcgaatgccatgggacgcTGGGCCGCCaaTACCCAtgCCCTaT
CcATGTTACGCTCCTCTGGACGGACTTTCTAACTCGGTcTCagttcgccggctcgGTTCTgtcctcccaaccagc
CgCCCAtcactCCCCCAcaaaaacagtggCGAAAGTGATGTGAACGGCTAGActtgctttcgggcggttgGGGGC
GTGTCaAcgCAGTTGGaAAAAAaAaCAcacacacacgcttacgCACACGGCGGCACACTTCGGCCGGTTGAGGTT
TccataTAtATGGCACCCCAGGGCTTAACGGCAAGTCATCCCgAGTGCCAAACAGCTGcCTCAAGTGccGCACTT
GAGccgatctggatcccggaacAGGGGGTTGCaacccgggggttgagtggggttgggcgttGGCTTTGTCGcTGT
TTAAGTAGTCCGGCAGATcGAGTGtGGATAAGATTTTTTCACAGCTGGCtgTCCtgccgttggtaccagccagta
cattcgccaacccctcgccaaacgaaaaaaaaaaacaagaacaagaaccccgcCTtGCACGGCGCcaacAACCTT
TGACTccggcccaactcccttcatggtgagccggaatcctgactgtccggaataagCTATTGACAGCTATTGTCt
tCGCCGGCAACAACAATGTTGACCTATTCtCAAgAtGTCACGGaTCgGGTTCAatGaAAAcTTcGTTTTgGgtta
AAAAATCaTTGCaACGGCtttacactactttTTcAGaAAATCGgtTgCaTTGTTTGTGCCGGCAAAaCTTTtgat
tgctaggcgaagaacaacccttaaagaagttcgccttgctaaccctttgagtttatctgctgaagaagttgcttg
ttaaccctttgagttgcgcttcaacccttttatccttgGCCGCCGGCagttgaaggaaagacagtattgtaaCGG
CtaaagttgagttgagattgggcgagTCTAGA

Yellow: Hox/Antp/Ap binding sites:
ATTA > CGGC
TAAT > GCCG
ATTAAT > CGGCCG
TAATTA > GCCGGC
ATTATTA CGGCGGC

eya-D Col CRM

CTCGAggatcacagaatccagcttgctgggtatgctgtagtcgacgcccttcgcacGtTCCGcCAATTTggctgc
gttgtttcttgggTTAAcCGTTgCCAgGCCTATGAAATggTGcttgcaaccactcgccccgtccggaaaaCaACA
ACattgAGGCATCGCTGACAAATTGAATTTGTAAACTTGtCCgcgggCAACAACAAATCATtGGAGACCCCCaGG
GATGCTGtttcggttgctgatccgtccgttgccataaccatccggattccggagtcagcaggaggAggaggagga
GGAGGAcgacgccgcccgacacggaggtggatgcgaatgccatgggacgcTGGTAATCCaaTACCCAtgCCCTaT

CcATGTTACGCTCCTCTGGACGGACTTTCTAACTCGGTcTCagttcgccggctcgGTTCTgtcctcccaaccagc
CgCCCAtcactCCCCCAcaaaaacagtggCGAAAGTGATGTGAAATTaTAGActtgctttcgggcggttgGGGGC
GTGTCaAcgCAGTTGGaAAAAAaAaCAcacacacacgcttacgCACAATTATTAACACTTATTAATGTTGAGGTT
TccataTAtATGGCACCCCAGGGCTTAAATTAAAGTCATCCCgAGTGCCAAACAGCTGcCTCAAGTGccGCACTT
GAGccgatctggatcccggaacAGGGGGTTGCaacccggggttgagtggggttgggcgttGGCTTTGTCGcTGT
TTAAGTAGTCATTAAGATcGAGTGtGGATAAGATTTTTTCACAGCTGGCtgTCCtgccgttggtaccagccagta
cattcgccaacccctcgccaaacgaaaaaaaaaaacaagaacaagaaccccgcCTtGCAATTAGCcaacAACCTT
TGACTccggcccaactcccttcatggtgagccggaatcctgactgtccggaataagCTATTGACAGCTATTGTCt
tCTAATTAAACAACAATGTTGACCTATTCtCAAgAtGTCACGGaTCgGGTTCAatGaAAAcTTcGTTTTgGgtta
AAAAATCaTTGCaAaTTAtttacactactttTTcAGaAAATCGgtTgCaTTGTTTGTTAATTAAAAaCTTTtgat
tgctaggcgaagaacaacccttaaagaagttcgccttgctaaccctttgagtttatctgctgaagaagttgcttg
ttaaccctttgagttgcgcttcaacccttttatccttgtaataattaagttgaaggaaagacagtattgtaaatt
ataaagttgagttgagattgggcgagTCTAGA

Site 1: **CCCCaGGGA** > **AAAAcTTTC**
Site 2: **CCCCAGGG** > **AAAACTTT**

dimm-CRM

ggatccACTAGTAACGGCCGCCAGTGTGCTGGaattcgcccttgtgccagagcaacgcgaatttaagcattggag
ttgagttggtggtaaaattgagtctggagcacaaaaacccttttttttgggatgatttatgctgctgccagtgct
gctctaaaaagcgccgaccaactatctttgatattggtaacaatttttggccttaaaatatttgtccgtaaacaa
aattcattagcagagaagtcagcaattaaacatatgtattataaatggaatgatttcttgaattatggttatccg
aaatataattttgtttttaatttattgacccaggactttttctgaaaaggaaaataaataaataaattttgt
tttggcgcgaatttcttttctgcatttatttccaattttagaacacatttcttttcacactatttcgaaatcgaa
tccacatcgataatgtaaaataacaccgaccgtcgccgcaagtccccatagaagtcgagaggctagtaGAGGAAG
AAgTCAAcgTCATCGTTAAATTTAATTAGGCATCAAATgacccaacagccgaGCTAAATCAaAAaCcAATGGCCT
GAAAgtGACCCgAGTGCaggcgaaattcaaccgcctggctcaaagggcggtcgcAACACTTTAAAAcACAATTAA
AAGTCAAAACGTCAtTcTAAAcGaAAGCCAGTGGaCAaCCCtCcaaGGACcgaAgAGcCgcCaatgaggcgccgt
agagaaagtgctgatgtgggatggagacatggcgctcacTTTACATAACATTTAACAAAATTATAATGAGacata
gaatggatgtatatcggtcgggggtgagtcttctactcccgtgtcgaatgttaatgggattatgtgaggaatac
attttaatatttatttgaagtattatcctagcgagataaccgcattaggtggtgtccattagaatgaattcaatt
accgccaaagctaagtggatcagaaggaaactataagagaaaatactaagagcaatttttatataattaccgca
aaatgcacatgtaaccaatgtggcctatctctgcgtggagcaacataattacagagcgagcagatgcgaccgaaa
tgtaaacagtgtttcttccgaacctaaatctttggaaggcatcaaatcaaagacactaGaagggcgaattCTGCA
GATATCCATCACACTGGCGGCCGC

dimm-D Col-CRM

ggatccACTAGTAACGGCCGCCAGTGTGCTGGaattcgcccttgtgccagagcaacgcgaatttaagcattggag
ttgagttggtggtaaaattgagtctggagcacaaaaacccttttttttgggatgatttatgctgctgccagtgct
gctctaaaaagcgccgaccaactatctttgatattggtaacaatttttggccttaaaatatttgtccgtaaacaa
aattcattagcagagaagtcagcaattaaacatatgtattataaatggaatgatttcttgaattatggttatccg
aaatataattttgtttttaatttattgacccaggactttttctgaaaaggaaaataaataaataaattttgt
tttggcgcgaatttcttttctgcatttatttccaattttagaacacatttcttttcacactatttcgaaatcgaa
GAACACGATataatgtaaaataacaccgaccgtcgAcgcaaTTCAAACGCTaagtcgagaggctagtaGAGGAAG
AAgTCAAcgTCATCGTTAAATTTAATTAGGCATCAAATgacccaacagccgaGCTAAATCAaAAaCcAATGGCCT
GAAAgtGACCCgAGTGCaggcgaaattcaaccgcctggctcaaagggcggtcgcAACACTTTAAAAcACAATTAA
AAGTCAAAACGTCAtTcTAAAcGaAAGCCAGTGGaCAaCCAGAACCTTCCcgaAgAGcCgcCaatgaggcgccgt
agagaaagtgctgatgtgggatggagacatggcgctcacTTTACATAACATTTAACAAAATTATAATGAGacata
gaatggatgtatatcggtcgggggtgagtcttctactcccgtgtcgaatgttaatgggattatgtgaggaatac
attttaatatttatttgaagtattatcctagcgagataaccgcattaggtggtgtccattagaatgaattcaatt
accgccaaagAGCCTGTTCtcagaaggaaactataagagaaaatactaagagcaatttttatataattaccgca
aaatgcacatgtaaccaatgtggcctatctAGTATGTTCgcaacataattacagagcgagcagatgcgaccgaaa
tgtaaacagtgtttcttccgaacctaaatctttggaaggcatcaaatcaaagacactaGaagggcgaattCTGCA
GATATCCATCACACTGGCGGCCGC

Conversion Pattern
A>C; T>G; C>A; G>T
tccacatcg: GAACACGAT
tccccatag: GAAAACGCT converting that site would create the following binding site with the upstream region ccgcaagGA. Conversion to AcgcaaTTC; after that conversion no possible col sites detectable
CtCcaaGGA: AGAACCTTC

Ctaagtgga: AGCCTGTTC
Ctgcgtgga: AGTATGTTC

dimm-D Hox-CRM

ggatccACTAGTAACGGCCGCCAGTGTGCTGGaattcgcccttgtgccagagcaacgcgaatttaagcattggag
ttgagttggtggtaaaattgagtctggagcacaaaaacccttttttttgggatgatttatgctgctgccagtgct
gctctaaaaagcgccgaccaactatctttgatattggtaacaattttggccttaaaatatttgtccgtaaacaa
aattccggcgcagagaagtcagcacggcaacatatgtcggctaaatggaatgatttcttgacggctggttatccg
aaatagccgttttgtttttgccgttattgacccaggactttttctgaaaaggaaaataaataaataaattttgt
tttggcgcgaatttcttttctgcatttatttccaattttagaacacatttcttttcacactatttcgaaatcgaa
tccacatcgagccggtaaaataacaccgaccgtcgccgcaagtccccatagaagtcgagaggctagtaGAGGAAG
AAgTCAAcgTCATCGTTAAATTgccggcGGCATCAAATgacccaacagccgaGCTAAATCAaAAaCcAATGGCCT
GAAAgtGACCCgAGTGCaggcgaaattcaaccgcctggctcaaagggcggtcgcAACACTTTAAAAcACAcggcA
AAGTCAAAACGTCAtTcTAAAcGaAAGCCAGTGGaCAaCCCtCcaaGGACcGaAgAGCCgcCaatgaGGCGCCgt
agagAAAGtgctgatgtgggatggagacatggcgctcacTTTACATAACATTTAACAAAcggcgccgGAGacata
gaatggatgtatatcggtcgggggtgagtcttctactcccgtgtcgaATGTgccgGgGcggctgtgaggaatac
atttgccgatttatttgaagtcggctcctagcgagataaccgccggcggtggtgtcccggcgaatgaattcacgg
cccgccaaagctaagtggatcagaaggaaactataagagaaaatactaagagcaatttttatagccggcccgca
aaatgcacatgtaaccaatgtggcctatctctgcgtggagcaacagccggccagagcgagcagatgcgaccgaaa
tgtaaacagtgtttcttccgaacctaaatctttggaaggcatcaaatcaaagacactagaagggcgaattCTGCA
GATATCCATCACACTGGCGGCCGC

Conversion Pattern
A>C; T>G; C>A; G>T
TAAT= gccg
ATTA= cggc

dimm-D Exd-CRM

ggatccACTAGTAACGGCCGCCAGTGTGCTGGaattcgcccttgtgccagagcaacgcgaatttaagcattggag
ttgagttggtggtaaaattgagtctggagcacaaaaacccttttttttgggaccggttatgctgctgccagtgct
gctctaaaaagcgccgaccaactatcttccggattggtaacaattttggccttaaaatatttgtccgtaaacaa
aattcattagcagagaagtcagcaattaaacatatgtattataaatggaaccggttcttgaattatggttatccg
aaatataattttgtttttaatttattgacccaggactttttctgaaaaggaaaataaataaataaattttgt
tttggcgcgaatttcttttctgcatttatttccaattttagaacacatttcttttcacactatttcgaaatcgaa
tccacatcgataatgtaaaataacaccgaccgtcgccgcaagtccccatagaagtcgagaggctagtaGAGGAAG
AAgTCAAcgTCATCGTTAAATTTAATTAGGCccggAATgacccaacagccgaGCTAAccggaAAaCcAATGGCCT
GAAAgtGACCCgAGTGCaggcgaaattcaaccgcctggctcaaagggcggtcgcAACACTTTAAAAcACAATTAA
AAGTCAAAACGTCAtTcTAAAcGaAAGCCAGTGGaCAaCCCtCcaaGGACcgaAgAGcCgcCaatgaggcgccgt
agagaaagtgcccgggtgggatggagacatggcgctcacTTTACATAACATTTAACAAAATTATAATGAGacata
gaatggatgtatatcggtcgggggtgagtcttctactcccgtgtcgaatgttaatgggattatgtgaggaatac
attttaatatttatttgaagtattatcctagcgagataaccgcattaggtggtgtccattagaatgaattcaatt
accgccaaagctaagtggccgggaaggaaactataagagaaaatactaagagcaatttttatataattaccgca
aaatgcacatgtaaccaatgtggcctatctctgcgtggagcaacataattacagagcgagcagatgcgaccgaaa
tgtaaacagtgtttcttccgaacctaaatctttggaaggcccggaccggaagacactaGaagggcgaattCTGCA
GATATCCccggCACTGGCGGCCGC

Nplp1-CRM WT

ggatccactagtaacggccgccagtgtgctggaattcgcccttctgccctttTGCAGCACcCACTTcCAAACCGA
TTtAAACCGTAACGGAAACACGGACGCGAagcTTTGccgTTtGCCAGCTGCCGTAacacTAaCGTTAGCCgCaAA
GTCGTCTGCTCGACTGCTTTctCTTGGCCAGGACCCTCGACTTTaTATAcCCGAGCGGCACCCCGGATAAGCTCa
CTCGCGGGAGCGAAACCGGAGCGCCGTCAACTGTTGCTCTTCtAtAGcCTCTCTGTTGCGTGTGCAGTGTCCTGT
TTCGTGGGatGCTatTGTAGGTAATGACGTCATGGcGGCTGCAGGACAGAGCTCTGTTgCTTcTtGGtaAGCTCT
GTGTATTCAGGCCAACCaGCAGCACAGTGcacagctacaAAGCTTTTCGATaTtaAATtggtattcgcaggttac
attattgattcattttgaaaatataatagaatgtttgcaggccaaaaaaaaattaaaatgaatccaagtccaaaa
taaaaaaattaatagaaaacccttccttagctacgatttttcgggtttcggtataatttgaattactcgaaaccg
ttttcttatcatttttctttcaacTtTGCaAGTTcaaTCCACTGtgCgCTgGttGCTTtGGGACCAATCTAAGAG
TTTTtgGTTTCTCTTAATTcGAACtTGAGCTgTacGCCATATGTtGGCGCTCtCAGCGGgGcCacTGTCCgTCGC
AGAACTTAACACTTaAATAaAAAaCcttgaatcaAACATATGCTTCAAatATTcTTTATTGGaAtatttaaaatc
acacTTgAAGTtAATcAgAgTAaTTAAAACACaaaTAGGcTTtgTTGAAATaTTTTTCTAaCTtttgtgTTtGta
tTtACTaTGTATgattacATTTAATCTGATTATCGgATAGCAATgGGCTGGAAATACATTATcAATCACATTTAC
TTTTcGaTTATTCAaAATAAacATTtttATtATttTCAttgattacattcggtttttaacctcgaatcttttcat

cgtataaacCAAATTAAaagcattggaattcaatgaagtacaaaagaactcccgattagaatggcgttgcagtga
cctgtctgtaagaattccgaaaatataaaataaccaaaaagccggaaagtaatgccAAcAGAtAgATTGCtgCAC
ccCtCccGCaGAAGcTAagTTTATGCAATTTcCGAATaCcATATTATGAAGCGTAAAatgttaaaaCCAGATCCc
CGGTTATaCCCaaAaCCCtgAGTGCgGCaAGaGCAaTGCACAAAaTGCAGGGGCGGTCACAatCATaTGCCTACG
TCAGTAaAGCAAAtCGCTGTGGGGGTTTCcCCGCGTCACtCCtAAAAGaAGGATATATgGcgGATGGcagatgaA
GCGgCGGATGTAGCAGATTGCACGccggtacagacagctggcagggAAACcTGtCAGCGCActgcaacataggaG
GTTGGaGCCaGCGGTACTGtCGGCtTTTGcTtCaAcCAATccatgcaagggcgaattCTGCAGATATCCATCACA
CTGGCGGCCGC

Nplp1-D Col CRM

ggatccactagtaacggccgccagtgtgctggaattcgcccttctgccctttTGCAGCACcCACTGaACCCAATA
TTtAAACATGCCATTAAACACTGACGCGAaTcTTTGccgTTtGCCAGCTGCCGTAacacTAaCGTTAGCCgCaAA
GTCGTCTGCTCGACTGCTTTctCTTGGCCAGGACCCTCGACTTTaTATAcACGAGCTTCCAAAATTCTAAGCTCa
AGATATTTCGCGAAACCGGAGCGCCGTCAACTGTTGCTCTTCtAtAGcCTCTCTGTTGCGTGTGCAGTGTCCTGT
TTCGTGGGatGCTatTGTAGGTAATGACGTCATGGcGGCTGCAGGACAGAGCTCTGTTgCTTcTtGGtaAGCTCT
GTGTATTCAGGCCAACCaGCAGCACAGTGcacagctacaAAGCTTTTCGATaTtaAATtggtattcgcaggttac
attattgattcattttgaaaatataatagaatgtttgcaggccaaaaaaaaattaaaatgaatccaagtccaaaa
taaaaaaattaatagaaacccttccttagctacgattttcgggtttcggtataatttgaattactcgaaaccg
ttttcttatcatttttctttcaacTtTGCaAGTTcaaGAACAGTgtCgCTgGttGCTTtGGGACCAATCTAAGAG
TTTTtgGTTTCTCTTAATTcGAACtTGAGCTgTacGCCATATGTtGGCGCTCtCAGCGGgGcCacTGTCCgTCGC
AGAACTTAACACTTaAATAaAAAaCcttgaatcaAACATATGCTTCAAatATTcTTTATTGGaAtatttaaaatc
acacTTgAAGTtAATcAgAgTAaTTAAAACACaaaTAGGcTTtgTTGAAATaTTTTTCTAaCTtttgtgTTtGta
tTtACTaTGTATgattacATTTAATCTGATTATCGgATAGCAATgGGCTGGAAATACATTATcAATCACATTTAC
TTTTcGaTTATTCAaAATAAacATTtttATtATttTCAttgattacattcggttttaacctcgaatcttttcat
cgtataaacCAAATTAAaagcattggaattcaatgaagtacaaaagaactcccgattagaatggcgttgcagtga
cctgtctgtaagaattccgaaaatataaaataaccaaaaagccggaaagtaatgccAAcAGAtAgATTGCtgCAC
ccCtCccGCaGAAGcTAagTTTATGCAATTTcCGAATaCcATATTATGAAGCGTAAAatgttaaaaCCAGATCCc
CGGTTATaCCCaaAaCCCtgAGTGCgGCaAGaGCAaTGCACAAAaTGCAGGGGCGGTCACAatCATaTGCCTACG
TCAGTAaAGCAAAtCGCTGTGGGGGTTTCcCCGCGTCACgAAgCACCTaATTCTATATgGcgGATGGcagatgaA
GCGgCGGATGTAGCAGATTGCACGccggtacagacagctggcagggAAACcTGtCAGCGCActgcaacataggaG
GTTGGaGCCaGCGGTACTGtCGGCtTTTGcTtCaAcCAATccatgcaagggcgaattCTGCAGATATCCATCACA
CTGGCGGCCGC

TcCAAACCG > GaACCCAAT
CGTAACGGA > ATGCCATTC
CACCCCGGA > ACAAAATTC

CTCGCGGGA > AGATATTTC

TCCACTGtg > GAACAGTgt

tCCtAAAAG > gAAgCCCCT

Special Cases of new possible Col site creation after basepair conversion:
ATGCCATTAaacacTTC: Converting the first Col site would place a C in the end. This creates a new Col binding site with the downstream base pairs. So the last GGA basepairs were changed to TTC to take out the newly created possible Col binding site.
After conversion the sequence looks like following: TTCCGCGAag: This sequence resembles a reverse Col site again. So a final conversion of the Initial TTC into a GA and the final G into a T is applied.

acgagcTTCCAAAATTC: same strategy applied here
gAAgCACCTaaTTC: same strategy applied here

Nplp1-D Hox CRM

ggatccactagtaacggccgccagtgtgctggaattcgcccttctgccctttTGCAGCACcCACTTcCAAACCGA
TTtAAACCGTAACGGAAACACGGACGCGAagcTTTGccgTTtGCCAGCTGCCGTAacacTAaCGTTAGCCgCaAA
GTCGTCTGCTCGACTGCTTTctCTTGGCCAGGACCCTCGACTTTaTATAcCCGAGCGGCACCCCGGATAAGCTCa
CTCGCGGGAGCGAAACCGGAGCGCCGTCAACTGTTGCTCTTCtAtAGcCTCTCTGTTGCGTGTGCAGTGTCCTGT
TTCGTGGGatGCTatTGTAGGGCCGGACGTCATGGcGGCTGCAGGACAGAGCTCTGTTgCTTcTtGGtaAGCTCT

GTGTATTCAGGCCAACCaGCAGCACAGTGcacagctacaAAGCTTTTCGATcggcAATtggtattcgcaggttac
cggcttgattcatttgaaaatagccgagaatgtttgcaggccaaaaaaaacggcaaatgaatccaagtccaaaa
taaaaaacggccgagaaaacccttccttagctacgattttcgggtttcggtagccgttgacggcctcgaaaccg
tttcttatcattttctttcaacTtTGCaAGTTcaaTCCACTGtgCgCTgGttGCTTtGGGACCAATCTAAGAG
TTTTtgGTTTCTCT**GCCG**TcGAACtTGAGCTgTacGCCATATGTtGGCGCTCtCAGCGGgGcCacTGTCCgTCGC
AGAACTTAACACTTaAATAaAAAaCcttgaatcaAACATATGCTTCAAatATTcTTTATTGGaAtatttaaaatc
acacTTgAAGTgccgcAgAggccggcAAACACaaaTAGGcTTtgTTGAAATaTTTTTCTAaCTtttgtgTTtGta
tTtACTaTGTATgcggccATT**GCCG**CTG**CGGC**TCGgATAGCAATgGGCTGGAAATAC**CGGC**TcAATCACATTTAC
TTTTcGcggcTTCAaAATAAacATTtttcggcTttTCAttgcggccattcggttttaacctcgaatcttttcat
cgtataaacCAA**CGGC**Aaagcattggaattcaatgaagtacaaaagaactcccgcggcgaatggcgttgcagtga
cctgtctgtaagaattccgaaaatataaaataaccaaaaagccggaaaggccgccAAcAGAtAgATTGCtgCAC
ccCtCccGCaGAAGcTAagTTTATGCAATTTcCGAATaCcAT**CGGC**TGAAGCGTAAAatgttaaaaCCAGATCCc
CGGTTATaCCCaaAaCCCtgAGTGCgGCaAGaGCAaTGCACAAAaTGCAGGGGCGGTCACAatCATaTGCCTACG
TCAGTAaAGCAAAtCGCTGTGGGGGTTTCcCCGCGTCACtCCtAAAAGaAGGATATATgGcgGATGGcagatgaA
GCGgCGGATGTAGCAGATTGCACGccggtacagacagctggcagggAAACcTGtCAGCGCActgcaacataggaG
GTTGGaGCCaGCGGTACTGtCGGCtTTTGcTtCaAcCAAtccatgcaagggcgaattCTGCAGATATCCATCACA
CTGGCGGCCGC

Hox binding sites: TAAT, ATTA convert into
ATTA > cggc, TAAT > gccg

Nplp1-D E-box CRM

ggatccactagtaacggccgccagtgtgctggaattcgcccttctgccctttTGCAGCACcCACTTcCAAACCGA
TTtAAACCGTAACGGAAACACGGACGCGAagcTTTGccgTTtGC**ACTAGT**CCGTAacacTAaCGTTAGCCgCaAA
GTCGTCTGCTCGACTGCTTTctCTTGGCCAGGACCCTCGACTTTaTATAcCCGAGCGGCACCCCGGATAAGCTCa
CTCGCGGGAGCGAAACCGGAGCGCCGT**ACCAGT**TTGCTCTTCtAtAGcCTCTCTGTTGCGTGTGCAGTGTCCTGT
TTCGTGGGatGCTatTGTAGGTAATGACGTCATGGcGGCTGCAGGACAGAGCTCTGTTgCTTcTtGGtaAGCTCT
GTGTATTCAGGCCAACCaGCAGCACAGTGcacagctacaAAGCTTTTCGATaTtaAATtggtattcgcaggttac
attattgattcatttgaaaatataatagaatgtttgcaggccaaaaaaaaattaaaatgaatccaagtccaaaa
taaaaaaattaatagaaaacccttccttagctacgattttcgggtttcggtataatttgaattactcgaaaccg
tttcttatcattttctttcaacTtTGCaAGTTcaaTCCACTGtgCgCTgGttGCTTtGGGACCAATCTAAGAG
TTTTtgGTTTCTCTTAATTcGAACtTGAGCTgTacGC**ACGCGT**TtGGCGCTCtCAGCGGgGcCacTGTCCgTCGC
AGAACTTAACACTTaAATAaAAAaCcttgaatcaAA**ACGCGT**CTTCAAatATTcTTTATTGGaAtatttaaaatc
acacTTgAAGTtAATcAgAgTAaTTAAAACACaaaTAGGcTTtgTTGAAATaTTTTTCTAaCTtttgtgTTtGta
tTtACTaTGTATgattacATTTAATCTGATTATCGgATAGCAATgGGCTGGAAATACATTATcAATCACATTTAC
TTTTcGaTTATTCAaAATAAacATTtttATtATttTCAttgattacattcggttttaacctcgaatcttttcat
cgtataaacCAAATTAAaagcattggaattcaatgaagtacaaaagaactcccgattagaatgcgttgcagtgac
ctgtctgtaagaattccgaaaatataaaataaccaaaaagccggaaagtaatgccAAcAGAtAgATTGCtgCACc
cCtCccGCaGAAGcTAagTTTATGCAATTTcCGAATaCcATATTATGAAGCGTAAAatgttaaaaCCAGATCCcC
GGTTATaCCCaaAaCCCtgAGTGCgGCaAGaGCAaTGCACAAAaTGCAGGGGCGGTCACAat**ACgCGT**CCTACGT
CAGTAaAGCAAAtCGCTGTGGGGGTTTCcCCGCGTCACtCCtAAAAGaAGGATATATgGcgGATGGcagatgaAG
CGgCGGATGTAGCAGATTGCACGccggtacagacagctggcagggAAACcTGtCAGCGCActgcaacataggaGG
TTGGaGCCaGCGGTACTGtCGGCtTTTGcTtCaAcCAATCCATGCaagggcgaattCTGCAGATATCCATCACAC
TGGCGGCCGC

possible E-boxes, CANNTG; base exchange A > C ; T > G ; C > A ; G > T
cATATG > aCGCGT
CAcGTG > ACaTGT

CAtATG > ACgCGT
CATATG > ACGCGT
CAGCTG > ACTAGT
CAACTG > ACCAGT
CaGTTg > AcTGGt
CaATTG > AcCGGT
