# Supplementary material for: Neuronal cell fate specification by the molecular convergence of different spatio-temporal cues on a common initiator terminal selector gene
Source: PLoS Genet. 2017 Apr 17;13(4):e1006729. doi: 10.1371/journal.pgen.1006729 (PMC5411104; doi:10.1371/journal.pgen.1006729)
Supplement: S4 Data — Description of the identification of TFBSin the CRMS. (PDF) [file pgen.1006729.s010.pdf]

SUPPLEMENTAL DATA 4

Bioinformatics of Transcription Factor Binding Sites (TFBS)

Generation of EvoPrinter profiles

In order to mutate TFBS in cis-regulatory elements of the factors in the Nplp1 terminal selector specification cascade, we first analyzed the identified cis-regulatory elements for evolutionary conserved sequence blocks. The default setting of the online tool EvoprinterHD (https://evoprinter.ninds.nih.gov/evoprintprogramHD/evphd.html) was used to identify conserved sequences in the *Dmel* cis-regulatory DNA, by comparison the *D. melanogaster* sequence to the genomes of *D. simulans*, *D. sechellia*, *D. erecta*, *D. yakuba*, *D. ananassae* and *D. pseudoobscura.*

Possible TF core Binding sites identified and mutation process

Based on previous studies and database entries, (TRANSFAC, JASPAR) possible TFBS for the upstream factors which are assumed to interact with the identified cis-regulatory motifs, were identified. Possible core motifs were obtained by simple alignment of different potential binding sites for the same factor, if different studies found varieties of binding sites. Potential Kr binding sites were numerous but alignment led to a potential shared motif of TTAA. In the case of Pdm/POU binding, all possible POU interaction sites from the different protein domains (POU_S, POU_{HD} and POU octamer recognition site) were considered potential POU binding sites. Mutation of sequences was done by base pair conversion rather than deletion, in order not to alter structural characteristics of the enhancer elements. Changes of base pairs in potential binding sites was done according to the following procedure; A changed to G or C, T was changed to G or C, and vice versa. Mutated enhancer sequences were synthesized at Genscript (Piscataway, NJ, USA) cloned into the placZ.attB or pEGFP.attB vectors, via the same cloning sites as the wild type enhancer elements, and integrated in the same chromosomal positions, in order to minimize positional effects on the enhancer activity and to be able to compare wild type and TFBS mutant enhancer elements.

Sites mutated:

Hth: TGAC, TGGC, GTCA [1]
Exd: TGAT [2]
Cas: TTTT [3]
Homeobox-Q50 factors (Antp, Lbe, Ap): TAAT [4]
Dimm (E-box binding): CANNTG [5]
Col/Ebf1/Olf1: variations of the palindromic sequence ATTCCCNNGGGAAT [6]
Pdm/POU [7, 8]:

POU_S = POU specific domain binds gAATAT(G/T)CA, gAATATGCA or gAATATTCA. Not found in full length, but potential core motif TATGC/TATTC

POU_{HD}= POU homeodomain binds to (G/A)TAATNA, ATAATNA or GTAATNA

POU Domain recognition site is a(a/t)TATGC(A/T)AAT(t/a)t]ATGCAAAT

GATA: AGATAA (TRANSFAC® at www.biobase-international.com)
Kr: TTAACCCGTT [9]; AAAGGGTTAA [10]; AAAAGGGGTTAA [11]; AACCCTTT (JASPAR Database http://jaspar.genereg.net/); AAAGGGTT [12]; possible core sequences TTAA
