# Supplementary material for: Neuronal cell fate specification by the molecular convergence of different spatio-temporal cues on a common initiator terminal selector gene
Source: PLoS Genet. 2017 Apr 17;13(4):e1006729. doi: 10.1371/journal.pgen.1006729 (PMC5411104; doi:10.1371/journal.pgen.1006729)
Supplement: S1 Supplemental References — (PDF) [file pgen.1006729.s011.pdf]

SUPPLEMENTAL REFERENCES

1. Gebelein B, McKay DJ, Mann RS. Direct integration of Hox and segmentation gene inputs during Drosophila development. Nature. 2004;431(7009):653-9. Epub 2004/10/08. doi: 10.1038/nature02946. PubMed PMID: 15470419.
2. Ryoo HD, Marty T, Casares F, Affolter M, Mann RS. Regulation of Hox target genes by a DNA bound Homothorax/Hox/Extradenticle complex. Development (Cambridge, England). 1999;126(22):5137-48. Epub 1999/10/26. PubMed PMID: 10529430.
3. Kambadur R, Koizumi K, Stivers C, Nagle J, Poole SJ, Odenwald WF. Regulation of POU genes by castor and hunchback establishes layered compartments in the Drosophila CNS. Genes & development. 1998;12(2):246-60. PubMed PMID: 9436984.
4. Merabet S, Mann RS. To Be Specific or Not: The Critical Relationship Between Hox And TALE Proteins. Trends Genet. 2016;32(6):334-47. Epub 2016/04/14. doi: 10.1016/j.tig.2016.03.004. PubMed PMID: 27066866; PubMed Central PMCID: PMC4875764.
5. Kuzin A, Kundu M, Brody T, Odenwald WF. Functional analysis of conserved sequences within a temporally restricted neural precursor cell enhancer. Mechanisms of development. 2011;128(3-4):165-77. Epub 2011/02/15. doi: 10.1016/j.mod.2011.02.001. PubMed PMID: 21315151; PubMed Central PMCID: PMC3095431.
6. Hagman J, Belanger C, Travis A, Turck CW, Grosschedl R. Cloning and functional characterization of early B-cell factor, a regulator of lymphocyte-specific gene expression. Genes & development. 1993;7(5):760-73. Epub 1993/05/01. PubMed PMID: 8491377.
7. Kitamoto T, Salvaterra PM. A POU homeo domain protein related to dPOU-19/pdm-1 binds to the regulatory DNA necessary for vital expression of the Drosophila choline acetyltransferase gene. J Neurosci. 1995;15(5 Pt 1):3509-18. Epub 1995/05/01. PubMed PMID: 7751926.
8. Verrijzer CP, Alkema MJ, van Weperen WW, Van Leeuwen HC, Strating MJ, van der Vliet PC. The DNA binding specificity of the bipartite POU domain and its subdomains. The EMBO journal. 1992;11(13):4993-5003. Epub 1992/12/01. PubMed PMID: 1361172; PubMed Central PMCID: PMC556977.
9. Nibu Y, Senger K, Levine M. CtBP-independent repression in the Drosophila embryo. Molecular and cellular biology. 2003;23(11):3990-9. Epub 2003/05/16. PubMed PMID: 12748300; PubMed Central PMCID: PMC155210.
10. Pankratz MJ, Hoch M, Seifert E, Jackle H. Kruppel requirement for knirps enhancement reflects overlapping gap gene activities in the Drosophila embryo. Nature. 1989;341(6240):337-40. Epub 1989/09/28. doi: 10.1038/341337a0. PubMed PMID: 2797151.
11. Matyash A, Chung HR, Jackle H. Genome-wide mapping of in vivo targets of the Drosophila transcription factor Kruppel. The Journal of biological chemistry. 2004;279(29):30689-96. Epub 2004/05/08. doi: 10.1074/jbc.M403345200. PubMed PMID: 15131112.
12. Schroeder MD, Pearce M, Fak J, Fan H, Unnerstall U, Emberly E, et al. Transcriptional control in the segmentation gene network of Drosophila. PLoS biology. 2004;2(9):E271. Epub 2004/09/02. doi: 10.1371/journal.pbio.0020271. PubMed PMID: 15340490; PubMed Central PMCID: PMC514885.
